# The Krüppel-like factor 9 cistrome in mouse hippocampal neurons reveals predominant transcriptional repression via proximal promoter binding

**DOI:** 10.1186/s12864-017-3640-7

**Published:** 2017-04-13

**Authors:** Joseph R. Knoedler, Arasakumar Subramani, Robert J. Denver

**Affiliations:** 1 Neuroscience Graduate Program, The University of Michigan, Ann Arbor, MI 48109 USA; 2 Department of Molecular, Cellular and Developmental Biology, The University of Michigan, 3065C Kraus Natural Science Building, Ann Arbor, MI 48109 USA; 3 Current address: Department of Psychiatry and Behavioral Sciences, Stanford University, Stanford, CA 94305 USA

**Keywords:** Klf9, Chromatin, Transcription, Hippocampal neurons

## Abstract

**Background:**

Krüppel-like factor 9 (Klf9) is a zinc finger transcription factor that functions in neural cell differentiation, but little is known about its genomic targets or mechanism of action in neurons.

**Results:**

We used the mouse hippocampus-derived neuronal cell line HT22 to identify genes regulated by Klf9, and we validated our findings in mouse hippocampus. We engineered HT22 cells to express a Klf9 transgene under control of the tetracycline repressor, and used RNA sequencing to identify genes modulated by Klf9. We found 217 genes repressed and 21 induced by Klf9. We also engineered HT22 cells to co-express biotin ligase and a Klf9 fusion protein containing an N-terminal biotin ligase recognition peptide. Using chromatin-streptavidin precipitation (ChSP) sequencing we identified 3,514 genomic regions where Klf9 associated. Seventy-five percent of these were within 1 kb of transcription start sites, and Klf9 associated in chromatin with 60% of the repressed genes. We analyzed the promoters of several repressed genes containing Klf9 ChSP peaks using transient transfection reporter assays and found that Klf9 repressed promoter activity, which was abolished after mutation of Sp/Klf-like motifs. Knockdown or knockout of Klf9 in HT22 cells caused dysregulation of Klf9 target genes. Chromatin immunoprecipitation assays showed that Klf9 associated in chromatin from mouse hippocampus with genes identified by ChSP sequencing on HT22 cells, and expression of Klf9 target genes was dysregulated in the hippocampus of neonatal *Klf9*-null mice. Gene ontology analysis revealed that Klf9 genomic targets include genes involved in cystokeletal remodeling, Wnt signaling and inflammation.

**Conclusions:**

We have identified genomic targets of Klf9 in hippocampal neurons and created a foundation for future studies on how it functions in chromatin, and regulates neuronal morphology and survival across the lifespan.

**Electronic supplementary material:**

The online version of this article (doi:10.1186/s12864-017-3640-7) contains supplementary material, which is available to authorized users.

## Background

Krüppel-like factors (Klfs) comprise a family of zinc-finger transcription factors (TFs) that function in metabolism, development and oncogenesis [1]. They have a highly conserved DNA binding domain comprised of three zinc fingers that binds GC/GT rich regions in the genome. Members of this family are distinguished by their highly divergent N-terminal domains which recruit different chromatin modifying factors that in part govern whether the Klf functions as a transcriptional activator or repressor [1]. Krüppel-like factor 9 (Klf9; formerly basic transcription element binding protein 1 – BTEB1) was identified in a screen of a rat liver cDNA library for proteins that bind the basic transcription element (BTE), a GC-rich motif in the promoter of the rat *Cyp1a1* (Cytochrome P450) gene [2]. The zinc fingers of Klf9 have high sequence identity with those of Specificity protein 1 (Sp1), which binds to similar motifs and typically activates transcription [2]. Transient transfection assays showed that Klf9 repressed transcription from a reporter construct containing the BTE sequence. However, Klf9 activated transcription from a reporter containing six tandem repeats of the BTE, suggesting that its activity may be governed by the number of binding sites at a locus [2]. The N-terminal region of Klf9 contains two separable transactivation domains required for full activation of the six-repeat BTE promoter, and an α-helical motif that interacts with the repressor protein Swi-independent 3a (Sin3a) [3, 4].

In mouse central nervous system (CNS) *Klf9* expression is low at birth, rises postnatally, and peaks at approximately postnatal day (PND) 30 with highest expression in the hippocampus and cerebellum [5, 6]. This postnatal increase in *Klf9* expression depends on thyroid hormone (TH), which acts via its nuclear receptors to directly regulate transcription of the *Klf9* gene [7–10]. In immature and developing neurons, Klf9 mediates actions of TH on neurite extension [8, 11, 12]. However, in mature cortical neurons Klf9 inhibits neurite outgrowth [13], while in the cerebellum it mediates TH-dependent inhibition of axonal regeneration [14]. Thus, in neuronal cells Klf9 first promotes, then maintains the differentiated state. It is also required for survival of adult-born dentate granule neurons and Purkinje neurons of the cerebellum [15, 16], and it promotes oligodendrocyte differentiation and the expression of myelinating genes [17]. Consistent with these findings, Klf9-null mice show neurological defects, including deficits in fear conditioning and late-stage neurogenesis [6, 16]. In addition to its developmental role, *Klf9* is also an immediate-early gene that is upregulated in neurons by many extracellular stimuli, including TH [8], glucocorticoids [18], and electrical activity [16].

Despite evidence for a diversity of developmental and physiological roles for Klf9, very little is known about Klf9 genomic targets in any cell type, and nothing is known about the genes that Klf9 regulates in neurons. In the present study, we identified direct genomic targets of Klf9 in the mouse hippocampus-derived neuronal cell line HT22, which is a model for mature hippocampal neurons. This cell line expresses neuronal markers such as enolase and neurofilament proteins, but does not express the glial marker glial fibrillary acidic protein [19, 20]. We engineered several HT22 cell lines to allow for control of *Klf9* expression to study Klf9-dependent transcriptional responses by RNA sequencing (RNA-seq); to identify genomic regions where Klf9 associates in chromatin by chromatin-streptavidin precipitation followed by deep sequencing (ChSP-seq); and to investigate the consequences of *Klf9* deletion for gene transcription and cell proliferation (using CRISPR/Cas9 genome editing). We validated our findings in HT22 cells by analyzing Klf9 association in chromatin in mouse hippocampus by chromatin immunoprecipitation (ChIP) assay, and mRNA levels for putative Klf9 target genes in wild type and Klf9-null mouse hippocampus.

## Results

### Validation of engineered HT22 cell lines for the identification of Klf9 genomic targets

**Fig. 1:**
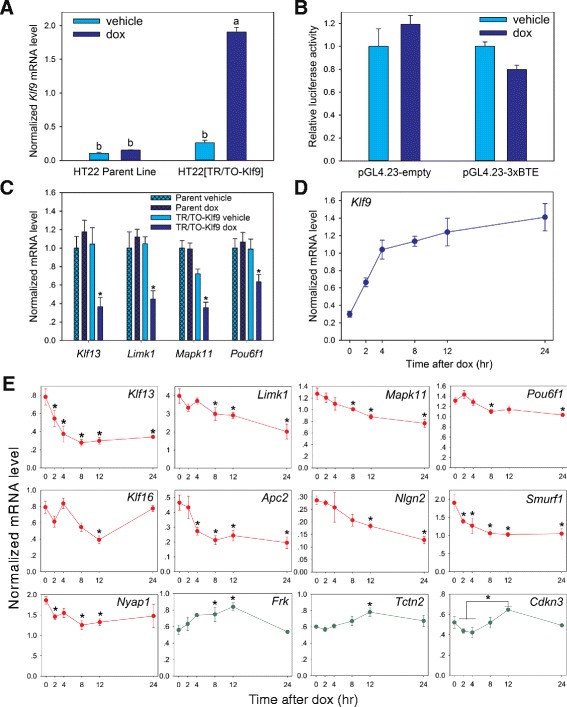
Identification of Klf9-regulated genes in HT22 cells by RNA-sequencing. **a** Treatment of HT22 [TR/TO-Klf9] cells with doxycycline (dox; 1 μg/ml) for 8 h increased *Klf9* mRNA ~10 fold compared to vehicle treated cells, but had no effect in parent HT22 cells. The baseline *Klf9* mRNA level did not differ between parent and [TR/TO-Klf9] line. Means with the same letter are not significantly different (*p* < .05; ANOVA followed by Tukey’s post-hoc test). **b** Dox-induced expression of Klf9 reduces luciferase activity from the pGL4.23-3xBTE plasmid, but not from pGL4.23-empty. The asterisk indicates a statistically significant difference by Student’s two-sample *t*-test (*p* < .05). **c** Validation by RTqPCR of four genes found to be repressed by Klf9 by RNA-seq. Treatment with dox for 8 h reduced mRNA levels for the four genes in HT22 [TR/TO-Klf9] but not in parent HT22 cells. The asterisks indicate statistically significant differences from parent cells treated with vehicle or dox, and from HT22 [TR/TO-Klf9] cells treated with vehicle (*p* < .05; ANOVA followed by Tukey’s post-hoc test). **d** Time-course showing induction of *Klf9* mRNA following treatment of HT22 [TR/TO-Klf9] cells with dox. **e** Validation of repression (9 genes) or induction (3 genes) of Klf9 target genes after treatment of HT22 [TR/TO-Klf9] cells with dox for different times. The asterisks indicate statistically significant differences from the zero time point (*p* < .05; ANOVA followed by Tukey’s post-hoc test)

We stably transfected HT22 cells with pCDNA4:TO-Klf9 and pCDNA6:TR vectors (see Methods), then isolated and screened seven clonal lines for baseline and doxycycline (dox)-inducible *Klf9* mRNA. One clonal cell line (2–1) was selected for further analysis. The mean baseline *Klf9* mRNA level of this cell line (hereafter referred to as HT22 [TR/TO-Klf9]) was slightly higher than that of the parent line, but this was not statistically significant (Fig. 1a). After treatment with dox for 8 h *Klf9* mRNA increased by ~10-fold (Fig. 1a; *F*_(3,8)_ = 480.974, *p* < .001; *n* = 3/treatment; ANOVA), which is within the physiological range seen following hormone treatment in HT22 cells, in neonatal mouse brain following hormone treatment [7], and in mouse brain during development [5].

We were unable to detect the endogenous (native) or recombinant (dox-induced) Klf9 protein by Western blotting on nuclear extracts of HT22 cells (data not shown), or with extracts from different cells/tissues of mouse or *Xenopus* using different antiserums (J.R. Knoedler, P. Bagamasbad and R.J. Denver, unpublished data). We therefore developed a bioassay that served as a proxy for the level of functional Klf9 protein in the cell. This assay comprised transient transfection of HT22 [TR/TO-Klf9] cells with a luciferase reporter vector containing three tandem repeats of the BTE sequence (pGL4.23-3xBTE), which supports Klf9-dependent transactivation or transrepression depending on the cell type [2, 10]; a promoter-less luciferase vector (pGL4.23) served as control. Treatment with dox for 8 h reduced luciferase activity by 20% in cells transfected with pGL4.23-3xBTE, but did not alter luciferase activity in empty pGL4.23 vector-transfected cells (Fig. 1b; t _(5)_ = 3.752, *p* < .05; Student’s two-sample *t*-test). To independently confirm that Klf9 represses activity from this promoter we co-transfected the parent HT22 cell line with pGL4.23-3×BTE and the pCS2-Klf9 expression vector. This produced a statistically significant reduction (39%) in luciferase activity compared with cells transfected with empty pCS2 vector (Additional file 1: Figure S1; t _(6)_ = 3.292*p* < .05; Student’s two-sample *t*-test). Taken together, our results show that *Klf9* mRNA can be induced within the physiological range by dox treatment of HT22 [TR/TO-Klf9] cells, and that this leads to the production of functional Klf9 protein.

### Identification of Klf9-regulated genes in HT22 [TR/TO-Klf9] cells by RNA sequencing

We conducted RNA sequencing (RNA-seq) on parent HT22 cells and the HT22 [TR/TO-Klf9] cell line treated with vehicle or dox for 8 h (*n* = 3/treatment for the parent line, 3 for HT22 [TR/TO-Klf9] vehicle treated and 2 for HT22 [TR/TO-Klf9] dox treated; a third replicate had to be discarded due to a technical error). We aligned sequencing reads to the mm8 build of the mouse genome using Bowtie [21], and differences in transcript abundance were quantified using DESeq [21, 22]. The parent HT22 cell line treated with dox showed no gene expression differences compared to parent cells treated with vehicle (false discovery rate [FDR]-adjusted *p* < .05), supporting that dox treatment does not have a significant impact on the HT22 transcriptome. We next compared the vehicle and dox-treated HT22 [TR/TO-Klf9] cells and found 238 differentially expressed genes, 217 downregulated and 21 upregulated (FDR-adjusted *p* < .005). The changes in mRNA level after 8 h of dox treatment ranged from − 1.2 to 1.52 (log_2_ fold change). The top 10 most strongly induced and repressed genes are listed in Table 1, and a list of all differentially regulated genes is given in Additional file 2: Table S1. We validated repression by Klf9 of 4 genes by RTqPCR on RNA isolated from parent and HT22 [TO/TR-Klf9] cells treated with vehicle or dox for 8 h (Fig. 1c; *Klf13*: *F*_(3,19)_ = 7.708, *p* < .005; *Limk1*: *F*_(3,17)_ = 6.417, *p* < .005; *Mapk11*: F _(3,19)_ = 22.107, *p* < .001; *Pou6f1*: *F*_(3,19)_ = 4.286, *p* < .05; *n* = 4–6/treatment; ANOVA).

**Table 1:** The top ten most up- or down-regulated genes by eight hr of forced Klf9 expression in HT22 [TR/TO-Klf9] cells (FDR-adjusted *p* value cutoff of .005)

Gene Symbol	Gene name	Log_2_ fold change
Klf13	Krüppel-like factor 13	−1.21
Rptoros	Regulatory associated protein of MTOR, complex 1, opposite strand	−1.12
Gpr161	G Protein-coupled receptor 161	−1.11
Apc2	Adenomatosis polyposis coli 2	−1.04
Zfp704	Zinc finger protein 704	−1.02
Arhgap39	Rho GTPase Activating Protein 39	−0.96
Armc7	Armadillo Repeat Containing 7	−0.95
Mex3a	Mex-3 RNA Binding Family Member A	−0.92
Wdfy2	WD Repeat And FYVE Domain Containing 2	−0.89
Nlgn2	Neuroligin2	−0.88
Frk	Fyn-Related Src Family Tyrosine Kinase	0.43
1810043G02Rik	RIKEN cDNA 1810043G02	0.46
4930430 F08Rik	RIKEN cDNA 4930430 F08	0.47
2610301B20Rik	RIKEN cDNA 2610301B20	0.48
Tctn2	Tectonic Family Member 2	0.49
Arxes2	adipocyte-related X-chromosome expressed sequence 2	0.51
Mitd1	Microtubule Interacting and Transport Domain Containing 1	0.51
Rilpl2	Rab Interacting Lysosomal Protein-Like 2	0.51
Zfp930	Zinc-finger protein 930	0.52
Cdkn3	Cyclin-Dependent Kinase Inhibitor 3	0.56

The mRNA levels (counts) were evaluated by DESeq. Genes are ordered by log_2_ fold change (lowest to highest). A complete list of differentially regulated genes is given in The mRNA levels (counts) were evaluated by DESeq. Genes are ordered by log_2_ fold change (lowest to highest). A complete list of differentially regulated genes is given in Additional file 2: Table S1

To further validate our RNA-seq data set, and to investigate the kinetics of Klf9-dependent gene repression, we conducted RTqPCR on HT22 [TO/TR-Klf9] cells treated with dox for different times. We observed a statistically significant increase in *Klf9* mRNA by 2 h, which peaked at 4 h and remained elevated through 24 h of dox treatment (Fig. 1d; *F*_(5,16)_ = 14.97, *p* < .001; *n* = 4/time point; ANOVA). We then conducted RTqPCR on 10 Klf9-repressed and 6 Klf9-induced genes with log_2_ fold changes ranging from − 0.44 to − 1.12 for repressed, or 0.38 to 0.56 for induced genes. We validated 9 repressed genes (Fig. 1e; *Klf13*: *F*_(5,16)_ = 6.642, *p* < .001; *Limk1*: *F*_(5,15)_ = 4.048, *p* < .05; *Mapk11*: *F*_(5,16)_ = 4.787, *p* < .01; *Pou6f1*: *F*_(5,15)_ = 4.527, *p* < .05; *Klf16*: *F*_(5,14)_ = 6.914, p < 0.05; *Apc2*: *F*_(5,16)_ = 5.548, *p* < .005; *Nlgn2*: *F*_(5,16)_ = 3.185, *p* < .05; *Smurf1*: *F*_(5,16)_ = 4.689, *p* < .01; *Nyap1*: *F*_(5,16)_ = 3.474, *p* < .05; *n* = 4/time point; ANOVA). Messenger RNA for *Hhipl* was unaffected (data not shown). However, of the 6 Klf9-induced genes tested, we could validate only 3 (*Frk*, *Tctn2* and *Cdkn3*; *Mitd1*, *Rilpl2* and *Ostm1* mRNAs were unaffected; data not shown). The mRNAs of the 3 induced genes that we validated were increased following 8–12 h of dox treatment, but returned to baseline by 24 h (Fig. 1e; *Frk*: *F*_(5,12_ = 4.721, *p* < .05; *Tctn2*: *F*_(5,16)_ = 3.055, *p* < .05; *Cdkn3*: *F*_(5,14)_ = 3.736, *p* < .05; *n* = 4/time point; ANOVA).

### Identification of sites across the HT22 genome where Klf9 associates in chromatin

**Fig. 2:**
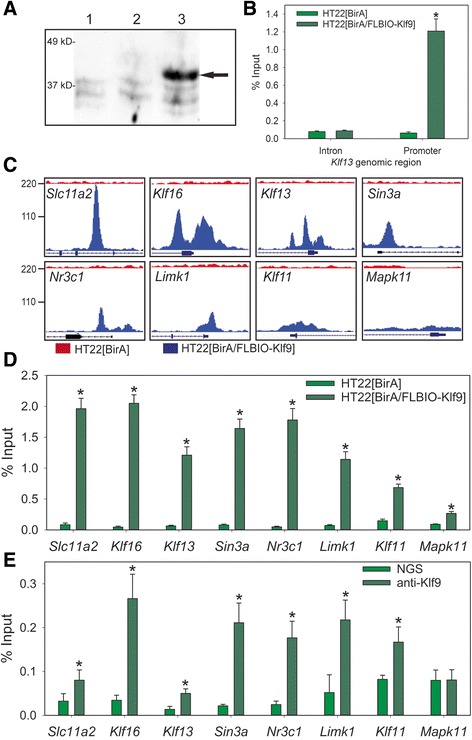
Identification of genome-wide association of Klf9 in HT22 cell chromatin using chromatin-streptavidin precipitation sequencing. **a** HT22 [BirA/FLBIO-Klf9] cells express biotinylated Klf9. Whole cell extracts from the HT22 parent cell line (lane 1), HT22 [BirA] cells (lane 2) or HT22 [BirA/FLBIO-Klf9] (lane 3) were fractionated on 10% SDS-PAGE and analyzed by Western blotting using streptavidin-HRP. **b** Chromatin-streptavidin precipitation gives ~25-fold enrichment at the *Klf13* promoter in HT22 [BirA/FLBIO-Klf9] cells compared with HT22 [BirA] cells. Precipitated DNA was analyzed by qPCR at the Klf13 promoter and intron (negative control region). The asterisk indicates a statistically significant difference by Student’s two-sample *t*-test (*p* < .0005). **c** Genome Browser (University of California, Santa Cruz) views showing the location of Klf9 peaks at eight Klf9-repressed genes. Top track = reads from cells expressing BirA alone; bottom track = cells expressing BirA + FLBIO-Klf9. The 5′ flanking region of each locus is shown; bars below the peaks represent exons, lines represent introns. **d** Validation of ChSP-seq peaks (shown in C) by targeted ChSP with quantitative PCR. Chromatin isolated from HT22 [BirA] and HT22 [BirA/FLBIO-Klf9] cells is compared. The asterisks indicate statistically significant differences analyzed by Student’s two-sample *t*-test (*p* < .005). **e** Klf9 associates with the same genomic loci in mouse hippocampus in vivo as in HT22 cells. Targeted chromatin immunoprecipitation (ChIP) assays for Klf9 were conducted on chromatin isolated from adult mouse hippocampus. The asterisks indicate statistically significant differences from the normal goat serum (NGS) IgG control analyzed by Student’s two-sample *t*-test (*p* < .05)

We engineered HT22 cells to express the *E. coli* biotin ligase BirA (HT22 [BirA]) or BirA plus a Klf9 fusion protein with an N-terminal FLAG tag and biotin ligase recognition peptide (HT22 [BirA/FLBIO-Klf9]) [23]. This allowed for high-affinity purification of Klf9 in chromatin by streptavidin precipitation (ChSP). We used Western blotting to detect the biotinylated fusion protein with streptavidin-HRP in HT22 nuclear extract (Fig. 2a). Previous work showed that Klf9 associates in chromatin with the *Klf13* 5′ upstream region in NIH 3 T3 cells (M. Nikiforov, unpublished results). We therefore investigated if Klf9 associated in chromatin with this genomic region in HT22 cells as proof-of-principle for the ChSP technique. First, we conducted ChIP assay for Klf9 on chromatin from the parent HT22 cell line. This resulted in ~5 fold enrichment above background (determined by ChIP with normal goat IgG) at the *Klf13* promoter but not at a *Klf13* intronic region which lacks Sp/Klf motifs (Additional file 3: Figure S2; intronic region, t _(6)_ = .850, *p* = .428; promoter region, t _(6)_ = 3.607, *p* < .05; *n* = 4/treatment; Student’s two-sample *t*-test). Next, we conducted ChSP on chromatin from HT22 [BirA] and HT22 [BirA/FLBIO-Klf9] cells. This resulted in ~25 fold enrichment above background (ChSP on chromatin from HT22 [BirA]) at the *Klf13* promoter but not at the same *Klf13* intronic region (Fig. 2b; t _(6)_ = −8.315, *p* < .0005; *n* = 4/cell line; Student’s two-sample *t*-test). These findings support that both native Klf9 and the FLBIO-Klf9 fusion protein associate in chromatin at the *Klf13* locus, and that the ChSP technique results in a greater signal/noise ratio than ChIP assay.

We conducted ChSP sequencing (ChSP-seq) on chromatin isolated from HT22 [BirA] and HT22 [BirA/FLBIO-Klf9] cells to identify sites of Klf9 association in chromatin. We used two independent peak calling programs (MACS and PePr) to identify genomic regions with higher densities of mapped reads [24, 25]. MACS analysis identified 8,841 peaks, while PePr analysis identified 3,382 peaks present in HT22 [BirA/FLBIO-Klf9] but not in HT22 [BirA] cells. All except four of the peaks called by PePr were also called by MACS; the peaks called by both programs were more enriched (larger difference in the number of mapped reads in HT22 [BirA/FLBIO-Klf9] compared to HT22 [BirA] cells) than those called only by MACS (not shown). We restricted further analysis to only those peaks called by both programs (total = 3,378), and then applied the program PeakSplitter to identify and subdivide regions with multiple closely spaced peaks [26]. Peaksplitter is only compatible with MACS; we therefore analyzed the MACS dataset with Peaksplitter, then used the program BedTools to extract the overlap of these split peaks with the peaks called by PePr [27]. Examples of how this approach classifies peaks are shown in Additional file 4: Figure S3. This approach gave a final count of 3,514 Klf9 peaks, which ranged from 8 to 2,429 bp in length (average length 881 bp). All peak coordinates, nearest gene and average sequencing read density across the peak (based on build mm10 of the mouse genome) are given in Additional file 5: Table S2. Sequencing and analysis of ChSP DNA from HT22 [BirA] cells showed very few regions (20 by MACS, 52 by PePr) with higher mapped sequencing read density compared with HT22 [BirA/FLBIO-Klf9] cells. This demonstrates that the BirA-FLBIO platform allowed for identification of Klf9-associated genomic regions with very low background.

### Validation of Klf9 peaks identified by ChSP in HT22 cells

To validate the ChSP-seq dataset we analyzed 8 Klf9 peaks using targeted ChSP- and ChIP-qPCR assays. The aligned sequencing read densities from the 8 genomic regions are shown in Fig. 2c, with peaks arranged from largest (220 overlapping reads at maximum height for *Slc11a2*; upper left panel) to smallest (20 overlapping reads for *Mapk11*; lower right panel). The small peak at the *Mapk11* 5′ upstream region was detected by MACS but not by PePr (see also Additional file 4: Figure S3); we analyzed this region to investigate the lower limit of detection of the ChSP-seq data set. All genomic regions tested showed significantly higher signal with ChSP DNA from HT22 [BirA/FLBIO-Klf9] cells compared with HT22 [BirA] cells (Fig. 2d; *Slc11a2*: t _(6)_ = −10.981, *p* < .001; *Klf16*: *t*_(6)_ = −14.417, *p* < .001; *Klf13*: *t*_(6)_ = −10.981, *p* < .001; *Sin3a*: t _(6)_ = −10.135, *p* < .001; *Nr3c1*: t _(6)_ = −9.402, *p* < .001; *Limk1*: *t*_(6)_ = −8.561, *p* < .001; *Klf11*: t _(6)_ = −8.653, *p* < .001; *Mapk11*: *t*_(6)_ = −5.448, *p* < .005; *n* = 4/cell line; Student’s two-sample *t*-test). We also conducted ChIP assay for Klf9 on chromatin extracted from the parent HT22 cell line, which showed statistically significant Klf9 ChIP signal (compared to IgG from normal goat serum) at four genes tested (Additional file 6: Figure S4; *Klf16*: t _(4)_ = 6.285, *p* < .005; *Limk1*: t _(4)_ = 4.093, *p* < .05; *Nr3c1*: t _(4)_ = 3.778, *p* < .05; *Sin3a*: t _(5)_ = 3.159, *p* < .05; *n* = 4/treatment; Student’s two-sample *t*-test). The Klf9 ChSP signal was at the background level at intronic regions located 10 kb or more downstream from the identified Klf9 peaks at the *Klf16*, *Limk1* and *Nr3c1* genes (Additional file 7: Figure S5A).

### Klf9 associates in chromatin from mouse hippocampus with genomic regions identified by ChSP-seq in HT22 cells

To determine if Klf9 associates in chromatin in mouse hippocampus at genomic sites identified in HT22 [BirA/FLBIO-Klf9] cells, we conducted targeted ChIP assays using chromatin isolated from the hippocampal region of the brain of adult wild type mice (five male and five female). We analyzed the same 8 genomic regions described above for HT22 cells and found statistically significant Klf9 ChIP signal at 7 of the 8 regions in both males and females (Fig. 2e; *Slc11a2*: t _(13)_ = 2.260, *p* < .05; *Klf16*: *t*_(12)_ = .4.458, *p* < .001; *Klf13*: *t*_(13)_ = 3.752, *p* < .005; *Sin3a*: *t*_(12)_ = 5.659, *p* < .0005; *Nr3c1*: *t*_(10)_ = −2.684, *p* < .05; *Limk1*: *t*_(11)_ = 3.887, *p* < .005; *Klf11*: *t*_(16)_ = 2.246, *p* < .05; *Mapk11*: *t*_(16)_ = −.0265, *p* = .979; *n* = 10; Student’s two-sample *t*-test). Since there were no statistically significant differences between the sexes we pooled the data for analysis. The lack of Klf9 ChIP signal at the 5′ upstream region of *Mapk11* is consistent with this region having the lowest ChSP signal in HT22 [BirA/FLBIO-Klf9] cells (Fig. 2d). We did not detect Klf9 ChIP signal in chromatin from mouse hippocampus at intronic regions located 10 kb or more downstream from the identified Klf9 peaks in the *Klf13*, *Klf16*, *Limk1* or *Nr3c1* genes (Additional file 7: Figure S5B). Taken together, our findings support that the BirA/FLBIO platform applied to the HT22 cell line is a useful model for identifying Klf9 genomic targets in mouse hippocampus.

### Klf9 associates in chromatin primarily with proximal promoter regions

**Fig. 3:**
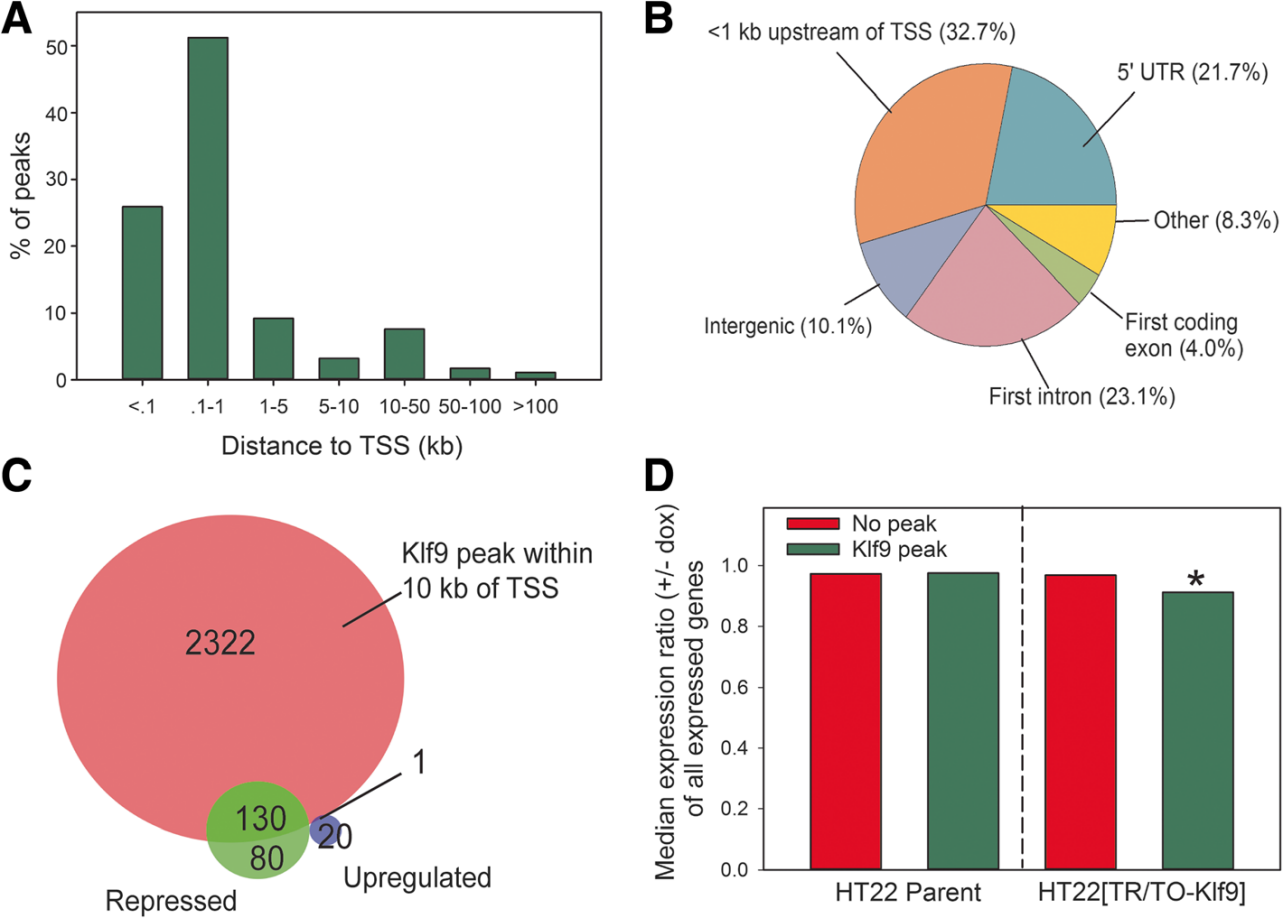
Klf9 associates near transcription sites and is more likely to be associated with repressed than induced genes. **a** Distribution of Klf9 peaks with respect to transcription start sites (TSS). **b** Distribution of Klf9 peaks with respect to gene features. **c** Overlap of Klf9-regulated genes with genes containing Klf9 peaks within 10 kb of their TSS. **d** The median expression ratio of all genes expressed in HT22 cells, with or without associated Klf9 peaks, in parent HT22 and HT22 [TR/TO-Klf9] cells treated with vehicle or doxycycline (1 μg/ml) for 8 h. In the parent cell line there was no expression difference after dox treatment whether or not the genes have Klf9 peaks associated (within 10 kb of their TSS; *p* = .057; Mann–Whitney *U*-test). In the HT22 [TR/TO-Klf9] cell line treatment with dox caused a statistically significant decrease in median expression ratio, but only for genes with associated Klf9 peaks (*p* < .001; Mann–Whitney *U*-test)

We used the ChIP-enrich web tool [28] to assign Klf9 peaks to genes based on the nearest transcription start site (TSS), and to identify where the peaks were distributed with respect to the TSSs of annotated genes. Based on this analysis, 89.5% of the peaks fell within 10 kb of a TSS, and of these, 86% (or 77.1% of total peaks) were 1 kb or less from a TSS (Fig. 3a). The ChIP-enrich software assigns peaks to genes based on the closest TSS, which results in some genes having multiple peaks associated with them. After accounting for such double-counted targets, this left 2,847 genes with at least one associated Klf9 peak (defined as being closer to that peak than any other annotated gene). The majority of these genes (2,749) had at least one Klf9 peak within 10 kb of their TSS.

We next used the HOMER peak annotation program to analyze the distribution of Klf9 peaks relative to the TSS. Of the peaks centered within 1 kb of a TSS, 32.7% were centered upstream, 21.7% were centered within the 5′ untranslated region, and 27.1% were centered either in the first exon or the first intron (Fig. 3b). We then used the Cis-regulatory Element Annotation System (CEAS) to map how sequencing reads were distributed with respect to genomic features [29]. Analysis of the distribution of mapped sequencing reads around TSSs revealed a moderate bias towards regions immediately upstream of the TSSs (Additional file 8: Figure S6). Thus, the larger peaks tend to be centered in 5′ flanking regions rather than in 5′ UTRs, exons or introns.

Of the 217 genes that we found to be repressed by Klf9 by RNA-seq, 130 (60%) have a Klf9 peak within 10 kb of their TSS. By contrast, of the 21 genes found to be induced by Klf9 only 1 (5%) had a peak within 10 kb of its TSS (Fig. 3c). This is consistent with Klf9 acting primarily as a transcriptional repressor. Differentially regulated genes without associated Klf9 peaks may be indirect target genes (regulated by other Klf9-responsive genes). If this is correct, it suggests that Klf9-induced genes are mostly indirect targets, while the majority of repressed genes are directly regulated by Klf9.

An additional 2,322 genes had at least one Klf9 peak located within 10 kb of their TSS, but their mRNA levels analyzed by RNA-seq were not significantly affected by forced Klf9 expression. However, when we looked at the mean mRNA level for all polyadenylated transcripts detected by RNA-seq in the HT22 [TR/TO-Klf9] cell line treated with vehicle or dox, and compared genes that did or did not have Klf9 peaks within 10 kb of their TSS, we found evidence for a general repressive action of Klf9 on transcription of genes possessing Klf9 peaks (Fig. 3d). We calculated the ratio of the mean mRNA levels with dox to that with vehicle for all expressed genes in each of the two cell lines (“expression ratio”). An expression ratio of 1 indicates no change caused by dox treatment, >1 indicates an increased mRNA level, and <1 a decreased mRNA level. In comparing the expression ratio of genes with or without Klf9 peaks, we found no statistically significant difference in the parent cell line, but a statistically significant lower median expression ratio for genes containing Klf9 peaks in the HT22 [TR/TO-Klf9] cell line (Fig. 3d; parent line: *p* = .057; HT22 [TR/TO-Klf9]: *p* < .001; Mann–Whitney *U*-test). This supports that Klf9 exerts a general repressive action on transcription of genes with which it associates.

### Identification of consensus Sp/Klf motifs at regions of Klf9 ChSP-seq peaks

**Fig. 4:**
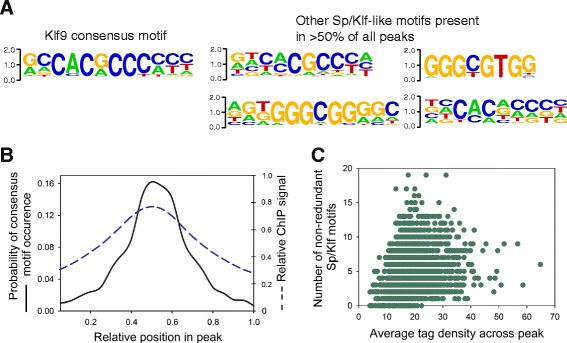
Sp/Klf motifs are enriched at Klf9 peaks, they correlate with peak features, and they are required for transcriptional repression by Klf9. **a** Position weight matrices showing the Klf9 consensus motif (*left*) and the four most commonly occurring Sp/Klf motifs that were partially redundant with the consensus motif (*right*). **b** Histogram showing the probability of the Klf9 consensus motif occurrence (solid) and average enrichment (number of mapped reads; dashed) across all Klf9 peaks. **c** Number of non-redundant Sp/Klf motifs correlates with the mean tag density across a peak (R = .245, *p* = .0000002)

We used the program HOMER to identify enriched DNA sequence motifs in Klf9 ChSP-seq peaks [30]. The most highly enriched sequence was a Sp/Klf motif (GCCACGCCCMCY) that was present in 75.6% of all peaks; hereafter we refer to this sequence as the ‘Klf9 consensus motif’ (Fig. 4a). We identified 18 additional motifs enriched at Klf9 peaks that are partially redundant with the Klf9 consensus motif. The top four most frequently observed Sp/Klf motifs (found in >50% of all peaks) are shown in Fig. 4a, and the remainder are listed in Additional file 9: Table S3. The Klf9 consensus motif, and the 18 additional motifs are hereafter collectively referred to as Sp/Klf motifs. At least one Sp/Klf motif was present in 98% of all Klf9 peaks, supporting that the presence of Sp/Klf motifs is important for targeting Klf9 in the genome. In addition, HOMER identified 18 significantly enriched motifs in Klf9 peaks other than the Sp/Klf motifs, the most significantly enriched of which (*p* < 1×10^−20^) matched previously reported binding sites for early growth response 2 (Egr2), ELK4, ETS transcription factor (Elk4), E2F transcription factor 1 (E2f1), Fos-like 2 (Fosl2), basic helix-loop-helix (bHLH), and regulatory factor X domain-containing 2 (Rfxdc2). A complete list of enriched motifs is given in Additional file 10: Table S4.

The Klf9 consensus motif tended to occur at or near the center of peaks, and the average density of mapped sequencing reads across the peaks closely matched the frequency of motif occurrence (Fig. 4b). The peaks contained between 0 and 19 Sp/Klf motifs (one outlier contained 48), with an average of 4.47/peak. There was a weak but statistically significant correlation between the number of non-redundant Sp/Klf motifs and average peak height (Fig. 4c; R = .245, *p* < .0001; Spearman Rank Order correlation).

We also used HOMER to analyze the promoters of the 21 genes that were induced by Klf9. Sequences of 1000 bp in length located upstream of the TSSs of these genes were downloaded from the UCSC genome browser. Scanning these sequences for the presence of Sp/Klf motifs found by *de novo* analysis of the Klf9 ChSP peaks found that only 4 of 21 (19%) contained one copy of the Klf9 consensus motif, while 17 of 21 (81%) had at least one Sp/Klf motif.

### Peak shape clustering reveals three separate categories of Klf9 peaks

**Fig. 5:**
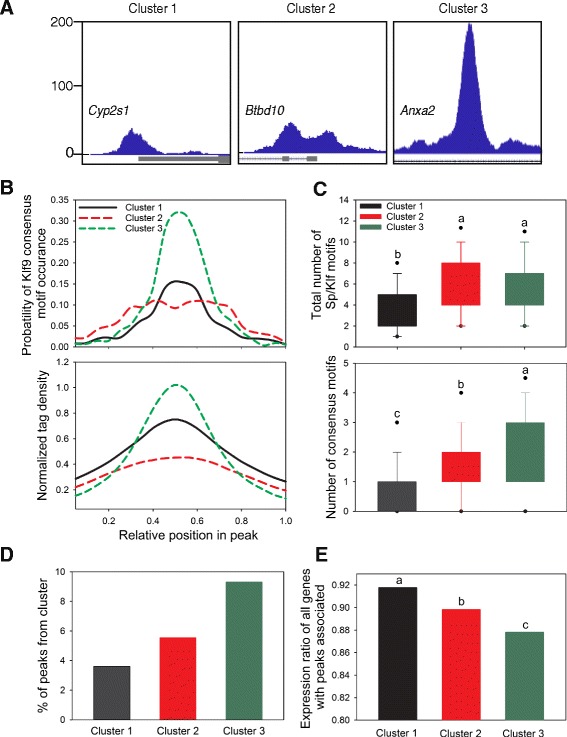
Klf9 peaks group into shape clusters that show different likelihood of association with repressed genes, and degree of transcriptional repression. **a** Klf9 peaks in HT22 cells can be grouped into three clusters based on shape and height characteristics. Example peaks from each cluster are shown. The graph height (200 pixels on UCSC genome browser) and viewing window size (2 kb) are held constant. **b** Distribution of the Klf9 consensus motif (top) and mapped sequencing read density (bottom) within peaks from each shape cluster. Sequencing read density closely tracks the probability of the occurrence of consensus motifs in all clusters. **c** Average number of Sp/Klf motifs and number of Klf9 consensus motifs present in peaks belonging to each cluster. Boxplots indicate 75% and 25% quantiles; dots indicate 5% and 95% quantiles. Means with the same letter are not significantly different (*p* < .05; Kruskal-Wallis non-parametric ANOVA). Top: peaks of clusters 2 and 3 contain more Sp/Klf-like motifs than those of cluster 1; bottom: peaks of cluster 3 have more copies of the Klf9 consensus motif than peaks of clusters 1 and 2. **d** Percentage of peaks of each cluster associated with repressed genes. **e** Median ratio of mRNA levels in HT22 [TR/TO-Klf9] cells treated +/− dox of genes associated with peaks from each cluster. Means with the same letter are not significantly different (*p* < .001; Kruskal-Wallis non-parametric ANOVA)

We analyzed Klf9 peaks using the program SIC-ChIP [31], which clusters peaks into subcategories based on five shape parameters (peak height, peak width at half-maximum height, peak area, number of local subpeaks, and shape index M (a measure of the peak’s topological complexity normalized to height) [32]. Boxplots of the distribution of each parameter in each cluster, and a scatterplot of how shape parameters correlate with each other and group peaks by cluster are shown in Additional file 11: Figure S7. By these criteria we divided the peaks into three categories, examples of which are shown in Fig. 5a. Peaks in Cluster 1 (2115 peaks) are of low average height and low complexity; peaks in Cluster 2 (812 peaks) are of low height but greater complexity (as measured by a higher M index and larger number of local subpeaks); peaks in Cluster 3 (549 peaks) are large and of low complexity.

The Klf9 consensus motif was highly enriched in peaks from all three categories. In genomic regions defined by Clusters 1 and 3, the motifs were located near the center of the peak, while in Cluster 2 the distribution was spread evenly across the peak (Fig. 5b, upper panel). The density of mapped reads (reflective of peak height) closely matched the distribution of Klf9 consensus motifs in all three clusters (Fig. 5b, lower panel). Sequencing read density (peak height) therefore correlates with the presence of Klf9 consensus motifs, even in wide peaks with multiple local maxima such as those seen in cluster 2. Clusters 2 and 3 had a larger number of Sp/Klf motifs per peak than Cluster 1 (Fig. 5c, upper panel; H _(2)_ = 528.802, *p* < .001; Kruskal-Wallis non-parametric ANOVA). There was no significant difference between Clusters 2 and 3 in average number of Sp/Klf motifs, but peaks from Cluster 3 contained more copies of the Klf9 consensus motif (Fig. 5c, lower panel; H _(2)_ = 245.055, *p* < .001; Kruskal-Wallis non-parametric ANOVA).

The proportion of peaks associated with Klf9-repressed genes differed among clusters, with Cluster 3 having the highest percentage, and Cluster 1 the lowest (Fig. 5d). This supports that large peaks with larger numbers of Sp/Klf and Klf9 consensus motifs are more likely to be associated with genes that are repressed by Klf9. In further support of this observation, the distribution of peaks from each cluster associated with repressed genes was nonrandom. Peaks from Cluster 1 accounted for 60.1% of all peaks, and 35% of repressed genes had at least one peak from Cluster 1 associated. In contrast, while peaks from Clusters 2 and 3 accounted for 23.1 and 15.6% of all peaks, respectively, 20.3 and 23% of all repressed genes had a peak from Clusters 2 or 3 associated (Additional file 12: Figure S8). Peaks from Clusters 2 and 3 are therefore more likely to be associated with repression by Klf9 than would be expected by chance (Chi-Square = 28.704 with 2° of freedom, *p* < .001).

We also calculated the expression ratio of genes with peaks from each cluster in vehicle vs. dox-treated HT22 [TR/TO-Klf9] cells. Genes with peaks from Cluster 2 show a lower expression ratio (indicating greater repression) than genes with peaks from Cluster 1, and genes with peaks from Cluster 3 have a lower expression ratio than from Cluster 2, supporting that Klf9 exerts a stronger repressive effect on transcription from peaks with either a greater number of consensus motifs or a higher ChSP signal (Fig. 5e; H _(2)_ = 45.181, *p* < .001; Kruskal-Wallis non-parametric ANOVA).

### Genomic regions where Klf9 associates support transcriptional repression by Klf9, and this requires intact Sp/Klf motifs

**Fig. 6:**
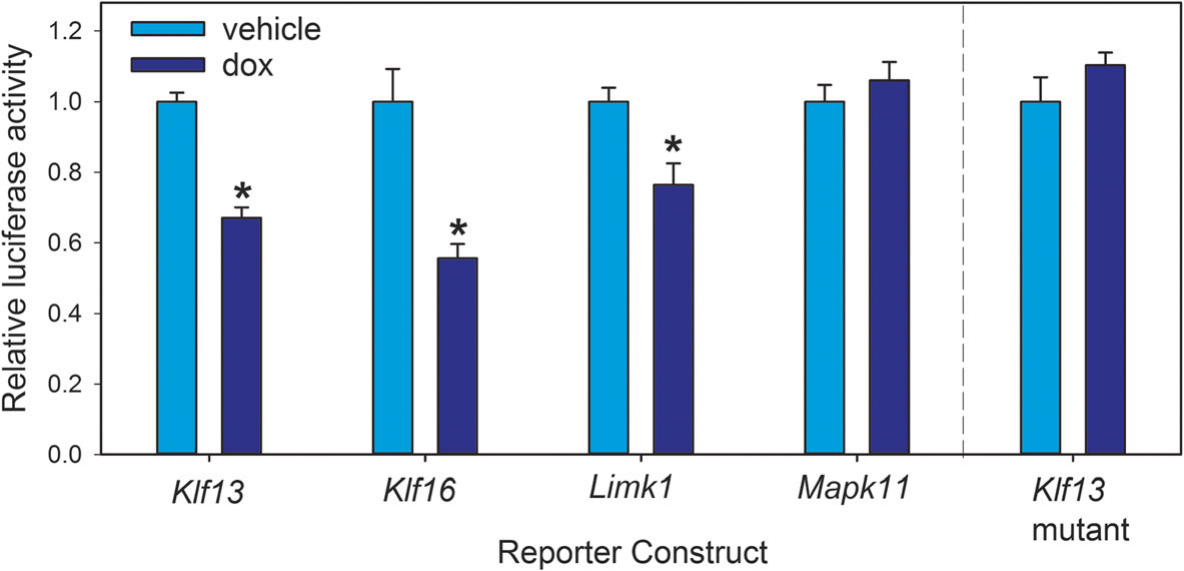
Klf9 represses transcription from synthetic promoters of Klf9 target genes, and Sp/Klf-like motifs are required for transcriptional repression by Klf9. We transfected HT22 [TR/TO-Klf9] cells with reporter constructs containing cloned DNA fragments corresponding to genomic regions with Klf9 peaks associated with the indicated genes (See Additional file 4: Figure S3 for genomic ranges). Forced expression of Klf9 repressed transcriptional activity from the *Klf13*, *Klf16* and *Limk1*, but not from the *Mapk11* promoter. Mutation of six Sp/Klf-like motifs in the *Klf13* promoter abrogated Klf9-dependent repression. The asterisks indicate a statistically significant difference from control by Student’s two-sample *t*-test (*p* < .01)

To determine if Klf9 can repress transcription of genes with which it associates in chromatin we transfected HT22 [TR/TO-Klf9] cells with pGL4.23 reporter constructs containing DNA fragments corresponding to genomic regions with Klf9 ChSP peaks: *Klf13* (439 bp; 6 Sp/Klf motifs, of which 3 were Klf9 consensus), *Klf16* (2192 bp; 17 Sp/Klf motifs, of which 6 were Klf9 consensus), *Limk1* (802 bp; 11 Sp/Klf motifs, of which 4 were Klf9 consensus) and *Mapk11* (886 bp; 6 Sp/Klf motifs, of which zero were Klf9 consensus) (see Additional file 4: Figure S3 for the ChSP peaks determined by PePr and the relative locations of the cloned DNA fragments, and Additional file 13: Table S5 for the DNA sequences and locations of Sp/Klf motifs within them). Treatment with dox for 24 h reduced luciferase activity from pGL4.23-Klf13 by 33%, pGL4.23-Klf16 by 45%, and pGL4.23-Limk1 by 19.5%; luciferase activity from the pGL4.23-Mapk11 vector was unaffected by dox treatment (Fig. 6; *Klf13*: t _(10)_ = 8.512, *p* < 10^−5^; *Klf16*: t _(10)_ = 4.430, *p* < .005; *Limk1*: t _(10)_ = 3.275, *p* < .01; *Mapk11*: t _(9)_ = 1.509, *p* = .166; *Klf13 mutant*: t _(10)_ = −1.331, *p* = .213; *n* = 6/treatment; Student’s two-sample *t*-test).

To determine if Sp/Klf motifs are required for repression by Klf9 we focused on the *Klf13* 5′ flanking region, which contains 6 Sp/Klf motifs (Additional file 13: Table S5). We used site-directed mutagenesis to convert these motifs to a series of 7 thymidines; the complete sequence of the *Klf13* DNA fragment and the location of the mutated nucleotides are given in Additional file 13: Table S5. Mutation of two of the 6 Sp/Klf motifs (sites 4 and 5), either individually or in combination, did not affect repression by Klf9 (data not shown). However, mutation of all six sites abolished Klf9-dependent transcriptional repression (Fig. 6).

### Forced expression of Klf9 promotes recruitment of Sin3a to some genomic regions with Klf9 peaks

Our RNA-seq experiment showed that Klf9 acts predominantly as a transcriptional repressor in HT22 cells. The N-terminus of Klf9 has a motif for interaction with the scaffolding repressor protein Sin3a [33] which recruits histone deacetylases to generate a compact chromatin structure and transcriptional repression. We therefore investigated whether Sin3a was recruited to Klf9 peaks by conducting ChIP assay for Sin3a on chromatin isolated from HT22 [TR/TO-Klf9] cells treated with vehicle or dox for 12 h. Treatment with dox increased the mean Klf9 ChIP signal at eight genomic regions corresponding to Klf9 ChSP peaks; this increase was statistically significant for six of the eight peaks (Additional file 14: Figure S9A). There were statistically significant increases in Sin3a ChIP signal following dox treatment at the *Klf16*, *Sin3a*, *Nr3c1* and *Limk1* genes, but it was unchanged at *Slc11a2*, *Klf13* and *Klf11*, and was reduced at *Mapk11* (Additional file 14: Figure S9B; *Slc11a2*: *t*_(7)_ = −4.628, *p* < .005; *Klf16*: *t*_(8)_ = −4.628, *p* < .005; *Klf13*: *t*_(10)_ = −2.829 *p* < .05; *Sin3a*: *t*_(9)_ = −2.138, *p* = .06; *Nr3c1*: *t*_(10)_ = −2.684, *p* < .05; *Limk1*: *t*_(8)_ = −2.334, *p* < .05; *Klf11*: *t*_(7)_ = −2.371, *p* < .05; *Mapk11*: *t*_(6)_ = −2.299, *p* = .06.; *n* = 6/treatment; Student’s two-sample *t*-test).

### Depletion of *Klf9* leads to dysregulation of Klf9 target genes

**Fig. 7:**
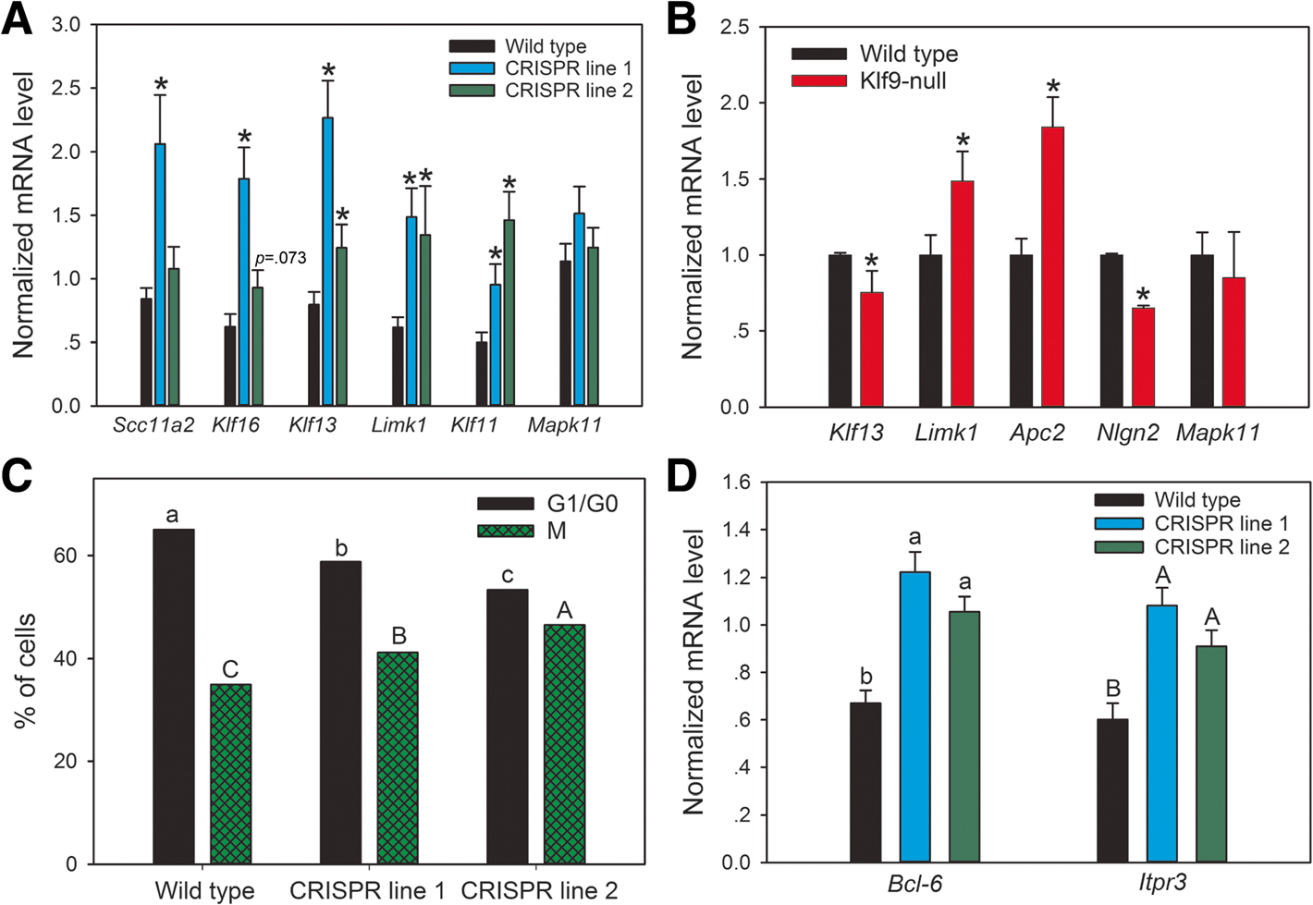
Klf9 target genes are dysregulated in *Klf9* HT22 depleted cells and in *Klf9* knockout mice, and depletion of Klf9 accelerates the cell cycle in HT22 cells. **a** Klf9 target genes are dysregulated in HT22 cells with Klf9 depleted by CRISPR/Cas9 genome editing. Asterisks indicate a statistically significant difference from the parent HT22 cell line (*p* < 0.05; ANOVA followed by Holm-Sidak post-hoc test). **b** Klf9 target genes are dysregulated in the hippocampus of postnatal day 7 *Klf9*-null mice. Asterisks indicate a statistically significant difference from wild-type mice by Student’s two-sample *t*-test (*p* < .05). **c** Cells with Klf9 depleted by CRISPR/Cas9 genome editing showed a higher percentage of cells in M phase (*gray bars*, *lowercase letters*) and a lower percentage in G1/G0 phase (*black bars*, *uppercase letters*). Means with the same letter are not significantly different (*p* < .05; ANOVA followed by Holm-Sidak post-hoc test). **d** The mRNA levels for two Klf9 target genes involved with cell cycle control are increased in Klf9 mutant HT22 cells. Means with the same letter are not significantly different (*p* < .05; ANOVA followed by Holm-Sidak post-hoc test)

To determine if loss of Klf9 alters the expression of Klf9 target genes we used CRISPR/Cas9 genome editing to generate Klf9 knockdown (CRISPR line 1) and knockout (CRISPR line 2) HT22 cell lines. A description of the mutations introduced into these cell lines is given in Additional file 15: Table S6. The CRISPR line 1 exhibited significantly higher mRNA levels compared to wild type for all Klf9-repressed genes analyzed except *Mapk11* (Fig. 7a; *Slc11a2*: *F*_(2,12)_ = 9.091, *p* < .005; *Klf13*: *F*_(2,12)_ = 14.267, *p* < .001; *Klf16*: *F*_(2,13)_ = 14.135, *p* < .001; *Limk1*: *F*_(2,13)_ = 5.875, *p* < .05; *Klf11*: *F*_(2,11)_ = 9.1, *p* < .01; *Mapk11*: *F*_(2,13)_ = .922, *p* = .422; *n* = 6/cell line; ANOVA). The CRISPR line 2 also had higher mean mRNA levels for all genes analyzed (again, except for *Mapk11*) and this was statistically significant for *Klf13*, *Limk1* and *Klf11*.

We also analyzed mRNA levels for a subset of Klf9 target genes identified in HT22 cells in the hippocampus of Klf9-null mice. *Klf9* mRNA in the mouse CNS is low at birth, then rises during the first 4 weeks of life, paralleling the postnatal rise in plasma TH concentration [34]. The mRNAs for *Klf13*, *Limk1*, *Apc2* and *Nlgn2* were dysregulated at PND7 in Klf9-null mice, although the direction of change differed among the genes (Fig. 7b; *Klf13*: *t*_(5)_ = −3.785, *p* < .01; *Limk1*, *t*_(5)_ = −2.763, *p* < .05; *Mapk11*: *t*_(4)_ = 2.777, *p* = .05; *n* = 4 animals/genotype; Student’s two-sample *t*-test). *Mapk11* mRNA was not different between wild type and Klf9-null mice. These expression differences disappeared by PND14 and remained unchanged at PND 60 (data not shown).

### Gene ontology analysis supports roles for Klf9 in neuronal morphology and function

We conducted gene ontology (GO) and pathway analysis using GeneCoDis on Klf9-repressed genes (Table 2) [35–37]. Four GO: PANTHER pathways were enriched among Klf9-repressed genes: “Cytoskeletal regulation by Rho GTPase”, “Inflammation mediated by chemokine and cytokine signaling pathway”, “Wnt signaling pathway”, and “B Cell Activation” (Table 2). We excluded the Klf9-induced genes from the pathway analysis because of the small number discovered and because we were able to validate only 3 of 6 tested.

**Table 2:** Genes repressed by forced Klf9 expression in HT22 [TR/TO-Klf9] cells and genes with Klf9 peaks associated in HT22 [BirA/FLBIO-Klf9] cells were subjected to pathway analysis using GeneCoDis

Panther ID	Pathway	Number of Klf9 target genes in pathway	Adjusted *p* value
All enriched pathways among genes repressed by Klf9			
Panther:P00016	Cytoskeletal regulation by Rho GTPase	3	0.034087
Panther:P00031	Inflammation mediated by chemokine and cytokine signaling pathway	5	0.041865
Panther:P00057	Wnt signaling pathway	5	0.041865
Panther:P00010	B cell activation	3	0.046343
The top ten most enriched pathways among genes with Klf9 peaks associated^a^			
Panther:P00006	Apoptosis signaling pathway	31	8.67E-11
Panther:P00034	Integrin signalling pathway	36	2.79E-10
Panther:P00031	Inflammation mediated by chemokine and cytokine signaling pathway	46	3.16E-10
Panther:P00016	Cytoskeletal regulation by Rho GTPase	23	1.28E-09
Panther:P00005	Angiogenesis	33	1.51E-09
Panther:P00057	Wnt signaling pathway	42	1.75E-08
Panther:P00056	VEGF signaling pathway	19	2.64E-08
Panther:P00047	PDGF signaling pathway	28	2.82E-08
Panther:P04393	Ras Pathway	20	7.85E-08
Panther:P00021	FGF signaling pathway	24	4.71E-07

Pathways are ordered from most- to least-enriched based on the false discovery rate (FDR)-adjusted *p* value

^**a**^ A complete list of enriched pathways is given in Additional file 16: Table S7

We then conducted pathway analysis on the set of all genes with Klf9 ChSP peaks. The most enriched PANTHER pathways among this set of genes were “Apoptosis”, “Integrin signaling pathway”, “Inflammation mediated by chemokine and cytokine signaling pathway” and “Cytoskeletal regulation by Rho-GTPase” (Table 2 and Additional file 16: Table S7). The overlap with the categories enriched among Klf9-repressed genes supports an important role for Klf9 in these pathways. We also conducted separate analyses on the genes with peaks of different shape clusters associated (see above). The top PANTHER pathways enriched in genes associated with Cluster 1 peaks were “Apoptosis”, “Wnt signaling pathway” and “Egf receptor signaling pathway”. In contrast, the top pathways enriched in the set of genes associated with Cluster 2 peaks were “Cytoskeletal regulation by Rho GTPase” and “Metabotropic glutamate receptor group II pathway”, while Cluster 3 peaks were associated with genes in the pathways “PDGF signaling”, “Fas signaling pathway” and “Cytoskeletal regulation by Rho GTPase” (Additional file 17: Table S8).

### Depletion of *Klf9* shortens cell cycle in HT22 cells and de-represses genes involved in cell proliferation

Previous work showed that Klf9 can reduce proliferation of different cell types [38–40]. One of the top GO: PANTHER pathways enriched among Klf9-repressed genes was ‘Wnt signaling’. The Wnt pathway has been shown to increase cell proliferation in diverse tissue types, including in neurons [41, 42]. We therefore looked at whether the cell cycle was altered in Klf9-deficient HT22 cells using flow cytometry. To facilitate our ability to observe differences in the cell cycle we cultured cells in reduced serum (2% vs. 10%; see Methods). We found that both CRISPR cell lines had a significantly higher proportion of cells in M phase (and a lower proportion in G1/G0) compared with the parent HT22 cell line (Fig. 7c; G1/G0 (lowercase): *F*_(2,8)_ = 56.27, *p* < .001; M (uppercase): *F*_(2,8)_ = 408.754, *p* < .001; *n* = 4/cell line; ANOVA). The mRNA levels for two confirmed Klf9 targets that are classified as Wnt-pathway related and are implicated in promoting mitosis, B cell CLL/Lymphoma 6 (*Bcl*-*6*) and inositol triphosphate receptor 3 (*Itpr3*), were significantly elevated in both CRISPR lines (Fig. 7d; *Bcl*-*6*: *F*_(2,9)_ = 19.797, *p* = 0.001; *Iprt3*: *F*_(2,9)_ = 13.346, *p* = 0.002; *n* = 6/cell line; ANOVA).

## Discussion

Here we report the first genome-wide analysis of Klf9 genomic targets in a mammalian neuronal cell type. Klf9 has been implicated in several aspects of neural development and regeneration, and is regulated by hormones [5, 7, 9, 10, 18], NFκB [7], oxidative stress [43], and electrical activity [16]. We therefore set out to identify its genomic targets in neurons to understand how it mediates transcriptional responses to these stimuli, and regulates neuronal differentiation, survival and plasticity. We identified Klf9-regulated genes and genomic regions where Klf9 associates in chromatin using the mouse hippocampus-derived neuronal cell line HT22. We show that Klf9 functions primarily as a transcriptional repressor, that it associates in chromatin predominantly near TSSs, and that it regulates transcription of genes involved in cytoskeletal remodeling, Wnt signaling and inflammation, among other pathways. Furthermore, by analyzing a subset of the genes and genomic regions identified in HT22 cells, we verified that these genes are *bona fide* Klf9 targets in mouse hippocampus in vivo, supporting that the HT22 cell line represents a useful model for mature hippocampal neurons, at least for the purpose of identifying candidate Klf9 target genes. Taken together, our findings represent an important advance in understanding the diverse developmental and physiological roles that Klf9 has in the mammalian central nervous system.

### Klf9 acts as a transcriptional repressor through association with proximal promoter regions

Findings from our RNA-seq experiment conducted in HT22 cells support that Klf9 acts predominately as a transcriptional repressor, with 10 times more genes repressed than induced. This is consistent with a recent study in glioblastoma cells that showed that Klf9 functions primarily as a transcriptional repressor [40]. Of the 10 Klf9-repressed genes that we attempted to validate by RTqPCR, we were able to confirm 9. However, of the 6 Klf9-induced genes tested by RTqPCR, we could validate only 3 (see Fig. 1e). Induction of these genes by Klf9 was transient, with all 3 genes returning to baseline by 24 h following dox treatment. By contrast, mRNA levels of 7 of the 9 validated repressed genes remained low at 24 h.

Using ChSP-seq we found that Klf9 associates predominantly with genomic regions within 1 kb of TSSs. Genes found to be repressed by Klf9 by RNA-seq were much more likely than induced genes to have Klf9 associated in chromatin (i.e., 60% of repressed genes had Klf9 associated, but only 5% of induced genes had Klf9 associated with their genomic regions). These findings support that Klf9 represses gene transcription by associating in chromatin at or near proximal promoters. In support of this, the presence of Klf9 at proximal promoter regions was associated with weak repression when averaged across all genes with peaks associated (see Fig. 3d). Because only one of the genes induced by Klf9 had a peak associated and the induced genes showed only transient induction, or failed to validate by RTqPCR, we conclude that they are either false positives or indirect targets of Klf9. Importantly, we found that of 10 peaks investigated, 9 had Klf9 associated with them in chromatin isolated from adult mouse hippocampus. This supports that HT22 cells can be used as a model to identify potential Klf9 genomic targets in adult mouse hippocampus, and to investigate Klf9 function in chromatin in mature neurons.

### Klf9 association in chromatin is strongly correlated with the presence of Sp/Klf motifs

The most enriched motif in Klf9 peaks was an Sp/Klf motif (GCCACGCCCMCY) that was present in 75% of all peaks; furthermore, 98% of all peaks had at least one Sp/Klf motif that was similar to the consensus motif. The consensus motif tended to occur near the center of peaks and its position correlated with the density of mapped reads, supporting that Klf9 binds directly to these DNA sequences.

Early studies on Klf9 function showed that it could act as a transcriptional activator or repressor depending on the number of Sp/Klf motifs present in the regulatory element [2, 3]. In CV-1 cells, Klf9 activated transcription from promoters containing multiple Sp/Klf motifs, but repressed transcription from promoters containing a single Sp/Klf motif. Most Klf9 peaks in HT22 cells (88%) had more than one Sp/Klf motif, but Klf9 acted almost exclusively as a repressor, suggesting that its regulatory activity is not necessarily related to the number of Sp/Klf motifs present. Other studies have shown that Klf9 can activate or repress the same promoter in the same cell type depending on the developmental stage [44]. The chromatin environment and the complement of interacting proteins may be more important than the promoter sequence for determining whether Klf9 acts as an activator or repressor.

An additional Sp/Klf-like motif different from the Klf9 consensus motif was present in 14.84% of peaks (Additional file 10: Table S4). This motif most closely matches the binding site for Sp1, which typically acts as a transcriptional activator [45]. Thus, the relative concentrations of Klf9, which likely acts as a repressor, and Sp1 in the cell may determine the transcriptional activity from these loci [46]. However, it is also possible that these are binding sites for other Sp/Klf proteins (including Klf9), which may act as repressors or activators of transcription.

Seventeen other enriched motifs similar to known TF response elements were identified by HOMER in Klf9 peaks (Additional file 10: Table S4). Several of these motifs are response elements for TFs important for neuronal structure and function. For example, two of the motifs in this list match binding sites for members of the immediate early gene families Egr and Fos (present in 28.55% and 21.21% of peaks respectively), which are involved in neural activity-dependent transcription [47]. Klf9 is also induced by neural activity [16], and thus may modulate the activity of these TFs, or vice versa, when recruited to common genomic regions..

Another enriched motif (present in 24.31% of peaks) was the E-box. The E-box is required for transcriptional activation by the core circadian transcription factors CLOCK and Bmal1, which form a regulatory feedback loop with Per and Cry proteins to orchestrate circadian rhythms in most tissues [48–51]. Several *Klf* genes have been found to exhibit circadian oscillations in their expression, including Klf9 [52]. Klf9 mediates circadian variation in cell proliferation in keratinocytes [38], but to our knowledge its role in circadian physiology in other tissues has not been investigated. One of the GO: PANTHER pathways enriched among genes with Klf9 peaks is “Circadian Clock System” (Additional file 16: Table S7). This suggests that Klf9 may co-regulate CLOCK/Bmal1 target genes, and thus modulate the core loop of the cellular circadian clock. While other Klfs have been shown to function as clock-output genes to orchestrate circadian physiology [53, 54], no Klfs have been shown to directly participate in the CLOCK/Per regulatory loop.

### Clustering analysis provides a basis for differentiating peaks associated with repressed genes

Different types of chromatin-associated proteins have different ChIP-seq profiles (e.g. broad regions for histones, discrete regions for TFs) and different genomic regions associated with the same factor may have different peak shapes [31]. We found that Klf9 peaks could be divided into three groups. Large, sharp peaks (Cluster 3) or broad, complex peaks (Cluster 2) were more likely to be associated with genes repressed by Klf9 than were smaller, less complex peaks (Cluster 1). In addition, the genes associated with Cluster 3 peaks were on average more strongly repressed after dox treatment on HT22 [TR/TO-Klf9] cells (see Fig. 5e).

The different peak characteristics could reflect at least three basic mechanisms: different numbers of Klf9 molecules recruited, perhaps related to the number of Sp/Klf motifs; differences in the chromatin environment (i.e., nucleosome density and histone modifications) that modulates accessibility of the locus; or local differences in the presence of protein interacting partners that may stabilize Klf9 association at the region. Peaks from Clusters 2 and 3 had more Sp/Klf motifs than peaks from Cluster 1 (Fig. 5c), which is consistent with recruitment of more Klf9 molecules as measured by height and area under the curve (Additional file 7: Figure S5C and D). Cluster 3 peaks had the same average number of Sp/Klf motifs as Cluster 2 peaks, but they had more copies of the Klf9 consensus motif (Fig. 5c); the high, sharp signal at these loci could reflect a higher affinity for Klf9 binding to the Klf9 consensus motif leading to more Klf9 association. Alternatively, or in addition, the chromatin environment at these regions may be more accessible, and/or there is greater recruitment of Klf9 interacting proteins that leads to increased crosslinking efficiency. Whether the peak characteristics result in differences in the kinetics and/or magnitude of transcriptional regulation of the associated genes requires investigation.

### Repression by Klf9 requires intact Sp/Klf motifs

To determine if the genomic regions where Klf9 associates are capable of supporting transcriptional repression, we isolated and subcloned several DNA fragments corresponding to peaks identified by ChSP-seq that were located in the 5’ upstream regions of Klf9-responsive genes. Forced expression of Klf9 repressed transcriptional activity supported by the *Klf13*, *Limk1* and *Klf16* upstream regions, but not by *Mapk11*. For the *Klf13* promoter we showed that Sp/Klf motifs are required for Klf9-dependent transrepression. Although the *Mapk11* region has five predicted Sp/Klf-like motifs, it lacks any copies of the Klf9 consensus motif. By contrast, the *Klf13*, *Limk1* and *Klf16* upstream regions that we investigated have 3, 4 and 6 Klf9 consensus motifs, respectively. Based on these findings we hypothesize that the Klf9 consensus motif is essential for mediating Klf9-dependent repression. The presence of multiple Sp/Klf motifs at most Klf9 peaks implies that such redundancy may be a feature of many, if not most, Klf9 genomic targets. These sites could also potentially act as binding sites for other Sp or Klf-like proteins, which suggests the potential for combinatorial regulation by a network of Klfs [55].

### Cellular pathways targeted by Klf9 in HT22 cells

To evaluate cellular pathways regulated by Klf9 in HT22 cells we analyzed GO: PANTHER enriched pathways among Klf9-repressed genes identified by RNA-seq, and also enriched pathways among genes with Klf9 peaks identified by ChSP-seq. The most enriched GO: PANTHER pathway among Klf9-repressed genes was “Cytoskeletal Regulation by Rho-GTPase”. This pathway was number 4 on the list of enriched pathways among Klf9 bound genes (see Table 2). Among the Klf9-regulated genes in this category are *Limk1*, which has been shown to promote neurite extension through inhibition of actin depolymerization [56], and *Mapk7* (ERK5), which positively regulates neurite extension and arborization in hippocampal neurons [57]. Several genes involved in actin polymerization, such as *Limk1*, were direct genomic targets of Klf9. The repression of positive regulators of actin polymerization such as *Limk1* is consistent with published findings showing that Klf9 represses dendritic and axonal remodeling and outgrowth in mature neurons [13, 14]. However, this does not explain how Klf9 promotes neurite elaboration and outgrowth in immature neurons [8, 12]. It may regulate a different set of genes, perhaps repressing repressors of growth, or it may upregulate rather than repress the same loci identified in this study at earlier developmental stages [44].

The second most enriched pathway for Klf9-repressed genes was “Inflammation mediated by chemokine and cytokine signaling pathway”, and this pathway was number 2 on the list of Klf9 bound genes. Several other Klfs have been implicated in immune and inflammatory responses [58]. For example, Klf4 promotes differentiation of monocytes and directly upregulates transcription of the pro-inflammatory cytokine interleukin-6 [59–62]. It also cooperates with the glucocorticoid receptor to induce expression of anti-inflammatory genes in keratinocytes [63]. Klf13 regulates expression of IL-4 in CD4 (+) T cells [64]. Klf10 has been found to regulate TGF-beta signaling in CD8 (+) T lymphocytes through modulating expression of TGF-beta receptor type II expression [65]. In contrast, Klf2 generally has anti-inflammatory actions [66]; for example, it inhibits inflammatory activation of monocytes through inhibition of the transcriptional activity of NF-κB [67] and blocks CD4 (+) T follicular helper cell differentiation [68]. However, Klf2 has also been shown to be required for activation of vascular endothelial cells in response to proinflammatory factors [69]. Klf6 has been shown to function as an accessory transcription factor for NF-kappa B [70]. To our knowledge, there have been no reports linking Klf9 to inflammatory signaling. However, *Klf9* is induced by glucocorticoids in mouse macrophages [71] and in neurons [7, 11, 18] via conserved glucocorticoid response elements [7, 18]. The repressive activity of Klf9 may serve as a counterbalance to pro-inflammatory Klfs such as Klf4, allowing for modulation of the immune response. It is also noteworthy that the Klf9 Synergy Module, which is an ultraconserved enhancer element located 4–6 kb upstream of the TSSs of tetrapod *Klf9* genes, contains a NFkB site to which P50 associates, and LPS can modulate Klf9 expression in HT22 cells [7].

The top enriched cellular pathway for genes with Klf9 ChSP peaks was apoptotic signaling. Interestingly, these genes are most strongly associated with peaks from Cluster 1; whereas, peaks from Clusters 2 and 3 are enriched in cytoskeletal and growth factor-related pathways, respectively (see Additional file 17: Table S8). Genes with peaks from Clusters 2 and 3 were more likely to be repressed by 8 h of dox treatment than genes from Cluster 1. If the kinetics and/or magnitude of transcriptional regulation varies among genes from the different peak clusters, with genes from Cluster 1 exhibiting slower kinetics than genes from Clusters 2 and 3, then one can propose the following model: Rapid induction of *Klf9* (e.g., in response to an acute stressor) suppresses morphological remodeling and cell proliferation by direct repression of a set of ‘first tier’ target genes (from Clusters 2 and 3). On the other hand, prolonged induction of *Klf9* in response to chronic stress may promote repression of anti-apoptotic genes and induction of pro-apoptotic genes (Cluster 1 peaks or ‘second tier’ targets), leading to cell death. This model might be supported by findings of a role for Klf9 in promoting cell death in response to chemotherapeutics and oxidative stress [43, 72].

### Depletion of Klf9 in HT22 cells leads to de-repression of Klf9 target genes

Knock-down or knock-out of *Klf9* in HT22 cells using CRISPR/Cas9 genome editing caused dysregulation of several Klf9 target genes. The genes *Klf13*, *Klf16* and *Limk1* were repressed by forced Klf9 expression, but were increased in Klf9 deficient HT22 cells, supporting a direct role for Klf9 in regulating transcription of these genes. The genes *Slc11a2* and *Klf11* were not repressed by forced Klf9 expression, but their mRNAs were increased following Klf9 depletion, and both genes had Klf9 peaks. *Mapk11* did not show any change in expression in CRISPR knockout cells despite its weak repression by forced Klf9 expression and the presence of a Klf9 peak at its proximal promoter region. The Klf9 peak at *Mapk11* was at the lower limit of detection by ChSP-seq (in fact, it was detected by the MACS algorithm, but not by PePr). ‘Weak’ peaks such as this may represent genes that are regulated under conditions of high Klf9 expression, but are not dependent on Klf9 for controlling baseline expression.

Despite the changes seen in HT22 cells, few differences in target gene mRNA level were seen in *Klf9* KO mice compared to age-matched wild-type animals, and what differences did exist tended to disappear after the first postnatal week (not shown). This suggests that there are compensatory mechanisms for regulating baseline mRNA levels in the absence of *Klf9*. The Klf family members 3, 5, 7, 10, 12, 13, 14 and 16 are all expressed in the adult mouse hippocampus, based on in situ hybridization data provided in the Allen Brain Atlas [73]. Some or all of these may contribute to compensatory regulation in the absence of *Klf9*. Functional compensation between Klfs has been demonstrated in embryonic stem cells, in which Klfs 2, 4 and 5 form a partially redundant network that maintains pluripotency. Knockout of all three is required to induce differentiation into fibroblasts [55]. *Klf13*, *14* and *16* are the Klf family members most similar to *Klf9* and are therefore the most likely candidates for compensating for loss of *Klf9* in the hippocampus [1]. In the mouse uterus Klf9 and Klf13 have partially overlapping functions such that Klf9 can partially compensate for the absence of *Klf13* [74]. We found that Klf9 associates with and represses transcription from the promoters of *Klf13* and *Klf16* in HT22 cells and associates with these genes in the mouse hippocampus in vivo. To our knowledge this is the first evidence of repression of direct cross-regulation between Klf9 and Klf13 and supports that these Klf proteins may regulate each other’s expression as well as activating or repressing shared targets. Investigation of the Klf13 and Klf16 cistromes in HT22 cells and the mouse hippocampus will be important to shed further light on the extent of cooperation or antagonism among Klf9, Klf13 and Klf16 in neurons.

### A role for Klf9 in modulation of the cell cycle in HT22 cells

Previous studies showed that Klf9 can suppress cell proliferation and/or promote apotosis [40, 72]. Consistent with these findings, we found that knockdown or knockout of Klf9 shortened the cell cycle in HT22 cells and increased expression of genes involved in cell proliferation. The Klf9-repressed genes *Bcl*-*6* and *Itpr3* are part of the Wnt signaling pathway. *Bcl*-*6* was shown to suppress apoptosis through induction of *cyclin d1*, and is sufficient to immortalize mouse embryonic fibroblasts [75]. In a human breast cancer cell line (MCF-7) knockdown of *Itpr3* caused cell cycle arrest, supporting that it is important for promoting tumor proliferation [76]. Furthermore, *Itpr3* can confer resistance to apoptosis [77]. The increase in expression of these two genes, and possibly other genes involved in cell proliferation and survival, may underlie the increase in cell proliferation that we observed after depletion of Klf9 in HT22 cells. This would be consistent with reports that Klf9 can act as a tumor suppressor and promote apoptosis in response to chemotherapeutics [39, 40, 72].

## Conclusions

We provide the first unbiased analysis of Klf9 genomic targets in mouse hippocampal neurons. We also provide strong evidence for a cross-regulatory network of Klfs that may be important for regulating the transcriptome. Our findings establish a foundation for understanding the molecular basis for Klf9’s effects on neuronal morphology and cell proliferation. Finally, we demonstrate the utility of HT22 cells for identifying genomic targets of TFs in the mouse hippocampus.

## Methods

### Plasmids

We obtained the HT22 cell line from Dr. David Schubert, the Salk Institute, La Jolla, CA. To engineer HT22 cells lines for controlled Klf9 expression we used the pRSV-BTEB plasmid (gift of Dr. Fuji-Kuriyama) as template to PCR-amplify the full-length Klf9 cDNA. We then directionally cloned the cDNA into the pCDNA4:TO (Invitrogen) and pEF1α-FLBIO (gift of Jianlong Wang; Kim et al., 2009) expression vectors at the XhoI/XbaI (pCDNA4:TO) or BamhI/XbaI (pEF1α-FLBIO) sites. To construct a vector to express the biotin ligase BirA we used the pEF1α-BirA plasmid (gift of Jianlong Wang) (Kim et al., 2009) as template to PCR-amplify a DNA fragment containing the EF1α promoter, the BirA coding sequence and the polyadenylation sequence. We subcloned this DNA fragment into the pSV40 zeocin plasmid (gift of Dr. Michael Uhler) at the NotI and NheI sites. Note that HT22 cells are resistant to G418 and the pEF1α-BirA plasmid has a neomycin cassette, so we needed to move the BirA sequence to a plasmid containing a different antibiotic resistance gene (zeocin). To express Klf9 in transient transfection assays we used pCS2-Klf9 [78].

We constructed a reporter plasmid (pGL4.23-3xBTE) containing three tandem repeats of the Basic Transcription Element (BTE) [2] by synthesizing complementary oligonucleotides and ligating the duplex oligonucleotide into pGL4.23 (Invitrogen, Carlsbad, CA) at the HindIII and NheI sites. We isolated the 5′ flanking regions of *Klf13* (439 bp), *Limk1* (800 bp), *Klf16* (2200 bp), and *Mapk11* (887 bp) by PCR using genomic DNA isolated from HT22 cells as template (the genomic DNA was isolated using the DNEasy DNA extraction kit (Qiagen, Hilden, Germany) according to manufacturer’s instructions) and subcloned the DNA into pGL4.23 at the SacI/HindIII, NheI/KpnI, NheI/HindIII, or SacI/XhoI sites to create pGL4.23 [*Klf13*] promoter, pGL4.23 [*Limk1*] promoter, pGL4.23 [*Klf16*] promoter and pGL4.23 [*Mapk11*] promoter, respectively.

Using the pGL4.23 [*Klf13*] promoter plasmid as template, we conducted site-directed mutagenesis of predicted Sp/Klf motifs using the Quikchange kit (Agilent Technologies, Santa Clara, CA). We converted seven nucleotides within each Klf9 consensus sequence (described below) to thymidines. The Klf13 5″ flanking region contains six predicted Sp/Klf motifs. We first generated two vectors with mutations in individual Sp/Klf motifs #1 or 2 (Additional file 13: Table S5), to create pGL4.23 [*Klf13*] promoter1mut and pGL4.23 [*Klf13*] promoter2mut), and one double mutant (Box #1 + 2; pGL4.23 [*Klf13*] promoter1 + 2mut). To generate a Klf13 promoter fragment with mutations in all six predicted Sp/Klf motifs we synthesized the entire 439 bp fragment corresponding to that in the pGL4.23 [*Klf13*] promoter with mutations in all six Sp/Klf motifs (Invitrogen) and subcloned this into pGL4.23 to create pGL4.23 [*Klf13*] promoter6mut. All oligonucleotides used for subcloning and site-directed mutagenesis are given in Additional file 18: Table S9.

### Generation and characterization of stable HT22 cell lines with doxycycline (dox)-inducible *Klf9*

We cultured HT22 cells (gift of Dr. David Schubert) in high-glucose DMEM (Invitrogen) supplemented with 10% fetal bovine serum (Hyclone Laboratories, Inc., Logan, UT), penicillin G (100 U/ml) and streptomycin sulfate (100 μg/ml) under a humidified atmosphere of 5% CO_2_ at 37 °C. This cell line was originally derived from mouse hippocampus using the Simian Virus 40 T antigen [19]. To generate stably transfected HT22 [TR/TO-*Klf9*] cell lines we seeded 5 × 10^6^ cells in 100 mm plates and transfected them with 5 μg each of pCDNA4:TO-Klf9 and pCDNA6:TR (Invitrogen) using Fugene6 (Invitrogen). Twenty four hr after transfection we changed to selective medium containing 100 μg/ml zeocin plus 5 μg/ml blasticidin (Research Products International, Mt. Prospect, IL). Louis, MO) and passaging with 0.25% trypsin (Gibco; Thermo-Fisher). We then seeded trypsinized cells in 6-well plates and expanded the clonal lines. We tested seven clonal HT22 [TR/TO-*Klf9*] lines for dox-inducible *Klf9* mRNA by culturing them in 6-well plates and treating with vehicle (0.1% DMSO) or 1 μg/ml dox (Sigma; all dox treatments were done at this concentration) for 8 h, after which cells were harvested for RNA isolation and analysis for *Klf9* mRNA levels by RTqPCR (described below). To test if the stable cell lines expressed functional Klf9 we seeded cells at 5 × 10^4^ per well in 24 well plates and co-transfected them with pGL4.23-3xBTE (200 μg) plus pRenilla (10 μg) (Promega Corporation, Madison, WI). We treated transfected cells with vehicle or dox for 8 h and harvested for dual luciferase assay (Promega). As a control, we co-transfected parent HT22 cells with pGL4.23-3xBTE plus a Klf9 expression vector (pCS2-Klf9) to independently investigate the action of Klf9 on this reporter.

For gene expression analysis we plated HT22 cells in either 6- or 12-well plates at densities of either 2.5 × 10^5^ or 1.25 × 10^5^, respectively, and began treatment with dox 24 h after plating. We then harvested cells and extracted RNA at different times after dox treatment for analysis by RTqPCR.

For luciferase assays, we cultured cells in 24-well plates at a density of 5 × 10^4^ cells/well. Twenty-four hr after plating we transfected with 200 ng/well of the luciferase vector and 10 ng/well pRenilla plasmid to monitor transfection efficiency, and 24 h later we treated cells with vehicle or dox for 8 h (pGL4.23-3xBTE vector) or 24 h (all other luciferase vectors). We harvested cells for luciferase assay using the Dual Luciferase Reporter Assay System (Promega) according to the manufacturer’s instructions. Firefly luciferase activity was quantified using a luminometer (Femtometer FB 12; Zylux Corp) and normalized to Renilla luciferase activity. All transfection reporter assays were repeated at least two times with 5–6 replicates/treatment.

### RNA extraction, reverse transcription and quantitative PCR

We extracted total RNA from HT22 cells or mouse brain (hippocampal region; see [18] for method) using the TRIzol reagent (Invitrogen) following the manufacturer’s instructions. We treated total RNA with DNase 1 (20U; Roche, Basel, Switzerland) to remove contaminating genomic DNA and conducted reverse transcription with 1 μg RNA using the High Capacity Reverse Transcription kit with ribonuclease inhibitor (Applied Biosystems, Life Technologies Corp, Foster City, CA). For RTqPCR we used Taqman assays for *Gapdh* and *Klf9* [7] and SYBR green assays for all other genes. All oligonucleotide primer sequences are given in Additional file 18: Table S9. We conducted RTqPCR using an ABI 7500 fast real-time PCR machine with Absolute qPCR low ROX mix (for Taqman assays) or Absolute qPCR SYBR low ROX mix (ABgene, Epsom, UK). We designed SYBR green assays using Integrated DNA Technology’s RealTime qPCR Assay tool; where possible we designed assays to span exon-exon boundaries. We used a relative quantitation method using serial dilutions of a cDNA pool to generate standard curves. We normalized all genes to the reference gene *Gapdh* whose mRNA was unaffected by treatments (data not shown.)

### Generation of stable HT22 cell lines that express biotinylated Klf9

We produced stably transfected HT22 cell lines that express biotinylated Klf9 by culturing cells in 100 mm plates and transfecting them with 5 μg each of pEF1α-BirA and pEF1α-FLBIO-Klf9 (HT22 [BirA/FLBIO-Klf9]). Twenty four hr after transfection we treated cells with 100 ug/ml zeocin plus 1 μg/ml puromycin. To make stably transfected HT22 cell lines that express only BirA (HT22 [BirA]) we transfected cells with 5 μg pEF1α-BirA. Twenty four hr after transfection we treated cells with 100 ug/ml zeocin. Following antibiotic selection for 5 days we isolated individual cells by trypsinization using cloning cylinders and subcultured them as described above.

To determine if the stably transfected cell lines expressed the FLBIO-Klf9 fusion protein and/or BirA-V5 we analyzed nuclear extracts by Western blotting. For nuclear extraction we cultured HT22 [BirA] or HT22 [BirA/FLBIO-Klf9] cells in 100-mm plates until they reached 95% confluency, collected cells with a cell scraper into hypotonic buffer (10 mM HEPES pH 7.9, 10 mM KCl, 1 mM DTT), allowed them to swell for 30 min, then lysed them with a motorized homogenizer and added 0.1 volume of sucrose restore buffer (50 mM HEPES pH7.9, 10 mM KCl, 1 mM DTT, 6.75% sucrose). We isolated nuclei by centrifugation at 4000 × g for 15 min, removed the supernatant (the cytoplasmic fraction), resuspended nuclei in nuclear extraction buffer (50 mM HEPES pH 7.9, .5 M KCl, 1 mM DTT) and incubated on ice for 40 min with vortexing every 10 min, then centrifuged for 1 h at 100,000 × *g*. We quantified the protein concentration of the extracts using the Pierce assay (Thermo Scientific). We then fractionated 40 μg of nuclear protein per lane of a 10% SDS-PAGE gel, transferred proteins to nitrocellulose membrane and blocked the membrane with SuperBlock (Thermo; following the manufacturer’s instructions; for BirA-V5), or with PBS containing 5% bovine serum albumin, 10% normal goat serum, 0.5% Triton-X (for FLBIO-Klf9). To detect BirA-V5 we incubated the membrane with V5 antibody (Millipore; 1:5000 dilution) overnight before washing and incubation with HRP-conjugated goat anti-rabbit secondary antibody for 1 h (Jackson ImmunoResearch Laboratories, Inc.); diluted 1:30,000). To detect FLBIO-Klf9 we incubated the membrane with Streptavidin-HRP (Thermo; diluted 1:1000) for 1 h. Immune or streptavidin-HRP complexes were revealed by chemiluminescence detection using Pierce ECL Substrate (ThermoFisher Scientific).

### RNA Sequencing

For RNA-seq analysis we treated the HT22 parent line or the HT22 [TR/TO-*Klf9*] line 2–1 with vehicle or dox for 8 h. We extracted total RNA using TRIzol reagent, then purified it using the QIAgen RNEasy kit. Library construction and next generation sequencing was done at the University of Michigan DNA Sequencing Core on twelve RNA samples (1 μg/sample) representing the four treatments (*n* = 3/treatment). The twelve samples were analyzed in two lanes using an Illumina 2000 HI-seq machine, which generated between 23,555,652 and 50,630,253 million reads per sample. The sequencing reads were de-multiplexed by the University of Michigan DNA Sequencing Core, evaluated and filtered using FastQC software (http://www.bioinformatics.babraham.ac.uk/projects/fastqc/) and aligned to the mouse genome (build mm8) using the program Bowtie [21]. We quantified mRNA levels using DESeq [22]. To calculate gene expression ratios, counts from all genes that were detected by RNA-seq in each cell line were average by treatment, and average counts from dox-treated cells were divided by average counts from vehicle-treated cells. Genes where no reads were mapped in one or both treatments were excluded from the analysis.

### Chromatin extraction and precipitation

Chromatin extraction and chromatin immunoprecipitation (ChIP) were done as described previously [5, 7, 18]. We grew HT22 cells in 100 mm plates, and for some experiments (e.g., preparation of samples for ChSP-seq libraries) we pooled cells from two plates to increase the amount of chromatin recovered. After washing with Dulbecco’s Phosphate Buffered Saline (DPBS), we treated cells with 1% formaldehyde for 10 min followed by treatment for 10 min with the bifunctional crosslinking agent dithiobis (succinimidyl propionate) (DSP; 200 nM; Thermo Scientific) before extracting chromatin. We sonicated the chromatin using an M220 Focused-Ultrasonicator (Covaris, Woburn, MA) for 20 min using a 2% duty factor, and checked that the DNA had been sheared to 500–600 bp by electrophoresis on a 1% Tris acetate EDTA agarose gel. We then flash-froze the chromatin with liquid nitrogen and stored it at − 80 °C until analysis. For each ChIP reaction we used 5 μg purified IgG of goat anti-mBTEB-C17, goat anti-mSin3a-K20, or normal goat IgG (Santa Cruz Biosciences, Santa Cruz, CA).

We conducted ChSP following the method of Ramadoss and colleagues [79]. We first washed MyOne T1 streptavidin-conjugated Dynabeads (Invitrogen; 50 ul/reaction) three times with phosphate buffered saline (PBS; pH 7.4), then mixed the beads with 50 μg of chromatin (5 μg of chromatin was reserved for input) and the total volume was brought to 1 ml with ChIP dilution buffer (.01% SDS, 1.1% Triton X-100, 1.2 mM EDTA, 16.7 mM Tris–HCl pH 8.1, 150 mM NaCl). The chromatin and beads were rocked overnight at 4 °C. The beads were then collected by magnet and the supernatant removed, then washed three times with 1 ml of 0.5× RIPA buffer (5 mM Tris-Cl pH 8.0, 0.5 mM EDTA, 0.25 mM EGTA, 70 mM NaCl, 0.5% Triton X-100, 0.05% sodium deoxycholate, 0.05% SDS). After the final wash, we resuspended the beads in decrosslinking buffer (50 mM Tris-Cl pH 8.0, 1 mM EDTA, 100 mM NaCl, 0.5% SDS) and removed crosslinks by incubation at 65 °C overnight (after this step the input samples were processed simultaneously with the ChSP samples). The DNA was then extracted with phenol:choloroform:isoamyl alcohol (Invitrogen) and precipitated with 0.3 M sodium acetate and 100% ethanol. We added 20 μg (1 μl) molecular biology-grade glycogen (Roche) before precipitation to visualize DNA pellets. After precipitation we resuspended the DNA in 25 μl nuclease-free water and analyzed by qPCR for the *Klf13* promoter and intronic regions. Samples were quantified as a percentage of corresponding input sample.

### Chromatin streptavidin precipitation sequencing

For ChSP sequencing (ChSP-seq) we prepared DNA precipitated from 150 μg of chromatin per sample as described above, and submitted ten samples to the University of Michigan DNA Sequencing Core for next generation sequencing: one input DNA sample (pooled from four input samples) from each of the two cell lines HT22 [BirA] and HT22 [BirA/FLBIO-Klf9], and four ChSP DNA samples from each cell line. We ran 5 samples per lane which generated between 18,818,940 and 27,816,127 million reads per sample. We filtered the sequenced reads using FastQC and aligned them to the mouse mm8 genome using Bowtie [21]. We then used the NCBI Genome Remapping Service to transfer peaks to the mm10 build. We identified peaks using MACS [25] and PePr software [24] and assigned peaks to genes using ChIP-enrich [28]. Additional analysis of the locations of peaks and mapping of regions of highest peak density was conducted using Cis-regulatory Element Annotation System software [29]. We clustered peaks by shape using the program SIC-ChIP [31]. Motif enrichment was analyzed using HOMER [30], and gene ontology analysis was done using GeneCoDis [35–37].

### Generation of HT22 Klf9-knockdown and knockout cells using CRISPR/Cas9 genome editing

We constructed a guide RNA (gRNA)/Cas9 expression plasmid (OriGene) containing the gRNA sequence 5′ ggggcgctccggaagccgag 3′ (this gRNA targets a sequence in the 5′ region of the *Klf9* gene). We transfected the parent HT22 cell line with this vector, which expresses enhanced green fluorescent protein (EGFP), and conducted fluorescence-assisted-cell sorting (FACS) at the University of Michigan Flow Cytometry core to isolate EGFP positive cells. We isolated clonal lines of the FACS-sorted cells as described above, extracted genomic DNA and screened for mutations by direct DNA sequencing of PCR-amplified DNA corresponding to *Klf9* exon 1 subcloned into the pGEM T-easy vector (Promega). We selected two Klf9-null lines (defined by the absence of a wild-type *Klf9* allele and the presence of mutations predicted to create nonsense or prematurely truncated proteins), cultured them simultaneously with the parent HT22 line in 6-well tissue culture plates, harvested cells, extracted RNA and analyzed gene expression by RTqPCR.

### Analysis of cell cycle by flow cytometry

Approximately 150,000 cells from the HT22 parent, CRISPR Line 1 and CRISPR Line 2 cell lines were plated in 10 cm plates in DMEM supplemented with 10% FBS. Sixteen hr after plating we changed the medium to DMEM containing 2% serum. After a further 72 h we trypsinized cells and pelleted them by centrifugation for 5 min at 200 × g. The cells were then washed by resuspending the pellet in DPBS containing 1% BSA and pelleting by centrifugation at 1000 rpm for 5 min. Cells were then fixed for 15 min at room temperature by re-suspending the pellet in 100 ul of 4% paraformaldehyde in DPBS. The fixed cells were then centrifuged at 1000 rpm for 5 min, then washed in DPBS containing 1% BSA and centrifuged again. Cells were permeabilized by resuspension in 100 μL of 1× Saponin buffer (Click-iT EdU Flow Cytometry Assay Kit, Invitrogen) containing 1% BSA. Cells were stained in darkness for 30 min at room temperature using FxCycle Violet (Thermo Fisher) diluted 1:1000 in DPBS, and analyzed using an Attune Cytometer (Applied Biosystems) at 405 nm wavelength. The relative proportion of cells in G1/G0 and M phases was determined using ModFit LT (Verity Software House, Topsham, ME).

### Animals

We purchased C57/BL6 mice from Jackson Laboratories (Sacramento, CA) and maintained them on a 12 L:12D photoperiod with food and water provided ad libitum. Animals were killed by rapid decapitation and a section of the brain that included the hippocampus was dissected and flash-frozen for subsequent chromatin or RNA extraction (see above). Mice null for Klf9 were bred from animals provided by Dr. Yoshiaki Fuji-Kuriyama and Dr. Frank Simmen [6]. All procedures involving animals were approved by the Institutional Animal Care and Use Committee of the University of Michigan.

### Statistical analysis

Derived (normalized) values from RTqPCR, ChIP, ChSP and dual luciferase assays were log_10_ transformed before analysis by one-way ANOVA followed by the Holm-Sidak multiple comparison test, or by unpaired Student’s *t*-test using SYSTAT (version 13; SPSS Inc., Chicago, IL). Data are reported as the mean ± standard error of the mean (SEM). When non-parametric tests were required, the Kruskal-Wallace one-way analysis of variance or Mann–Whitney *U*-Test were used. Results of the statistical analyses are reported in the figure legends.

## Additional files

Additional file 1: Figure S1. Forced expression of Klf9 by transient transfection of HT22 cells reduced activity of a synthetic promoter containing three copies of the basic transcription element (BTE). (TIF 3252 kb)

Additional file 2: Table S1. List of all genes that were up- or down-regulated after eight hours of doxycycline treatment of HT22 [TR/TO-Klf9] cells. (DOCX 46 kb)

Additional file 3: Figure S2. Klf9 associates in chromatin at the region of the Klf13 promoter in HT22 cells. (TIF 3325 kb)

Additional file 4: Figure S3. Comparison of computational approaches used to identify regions of Klf9 association in chromatin across the genome of HT22 cells. (TIF 29857 kb)

Additional file 5: Table S2. Genomic locations of all Klf9 ChSP peaks identified in HT22 [BirA/FLBIO-Klf9] cells based on coordinates of the mouse genome mm10 build. (XLSX 316 kb)

Additional file 6: Figure S4. Validation of regions of Klf9 association in chromatin in HT22 cells discovered by chromatin streptavidin precipitation sequencing, analyzed by targeted chromatin immunoprecipitation for Klf9. (TIF 3453 kb)

Additional file 7: Figure S5. Analysis of genomic regions in HT22 cells and mouse hippocampus that lacked Klf9 peaks by chromatin streptavidin precipitation (ChSP) sequencing. (TIF 4729 kb)

Additional file 8: Figure S6. Analysis of the distribution of mapped sequencing reads around transcription start sites (TSS) revealed a moderate bias towards regions immediately upstream of the TSSs. (TIF 13937 kb)

Additional file 9: Table S3. List of all Sp/Klf sequences identified as enriched above background in Klf9 ChSP peaks in HT22 [BirA/FLBIO-Klf9] cells. (DOCX 112 kb)

Additional file 10: Table S4. List of all DNA sequences found to be enriched above background at Klf9 ChSP peaks in HT22 [BirA/FLBIO-Klf9] cells. (DOCX 124 kb)

Additional file 11: Figure S7. Quantification of peak shape parameters from each cluster identified using the computer program SIC-ChIP. (TIF 23137 kb)

Additional file 12: Figure S8. A greater relative percentage of chromatin streptavidin precipitation (ChSP) sequencing peaks belonging to Clusters 2 and 3 are associated with genes repressed by Klf9 compared with peaks from Cluster 1. (TIF 3611 kb)

Additional file 13: Table S5. Subcloning of the 5′ upstream regions of *Klf13*, *Klf16*, *Limk1* and *Mapk11* into the pGL4.23 vector. (DOCX 15 kb)

Additional file 14: Figure S9. Validation of Klf9 association in chromatin in HT22 cells with the 5′ flanking regions of genes identified by chromatin streptavidin precipitation sequencing. (TIF 5685 kb)

Additional file 15: Table S6. Description of *Klf9* gene mutations introduced into HT22 cells by CRISPR/Cas9 genome editing. (DOCX 13 kb)

Additional file 16: Table S7. List of all GO: PANTHER pathways enriched in genes with associated Klf9 ChSP peaks. (DOCX 16 kb)

Additional file 17: Table S8. Genes with peaks from different clusters were subjected to pathway analysis using GeneCoDis. (DOCX 15 kb)

Additional file 18: Table S9. Oligonucleotides used for reverse transcriptase quantitative PCR (RTqPCR), chromatin immunoprecipitation assays, subcloning and site-directed mutagenesis. (DOCX 14 kb)

## Abbreviations

ANOVA: Analysis of Variance
BTE: Basic transcription element
ChIP: Chromatin immunoprecipitation
ChSP: Chromatin streptavidin precipitation
CNS: Central nervous system
Dox: Doxycycline
GO: Gene ontology
gRNA: Guide RNA
Klf: Krüppel-like factor
MACS: Model-based analysis of ChIP-seq
PANTHER: Protein Analysis Through Evolutionary Relationships
PePr: Peak Calling and Prioritization Pipeline
PND: Post-natal day
RTqPCR: Reverse transcription quantitative polymerase chain reaction
SIC-ChIP: Shape index clustering for ChIP-seq peaks
Sin3a: Swi-independent 3a
Sp: Specificity protein
TF: Transcription factor
TSS: Transcription Start Site
