# Supplementary figures and images for: The Krüppel-like factor 9 cistrome in mouse hippocampal neurons reveals predominant transcriptional repression via proximal promoter binding
Source: BMC Genomics. 2017 Apr 13;18:299. doi: 10.1186/s12864-017-3640-7 (PMC5390390; doi:10.1186/s12864-017-3640-7)

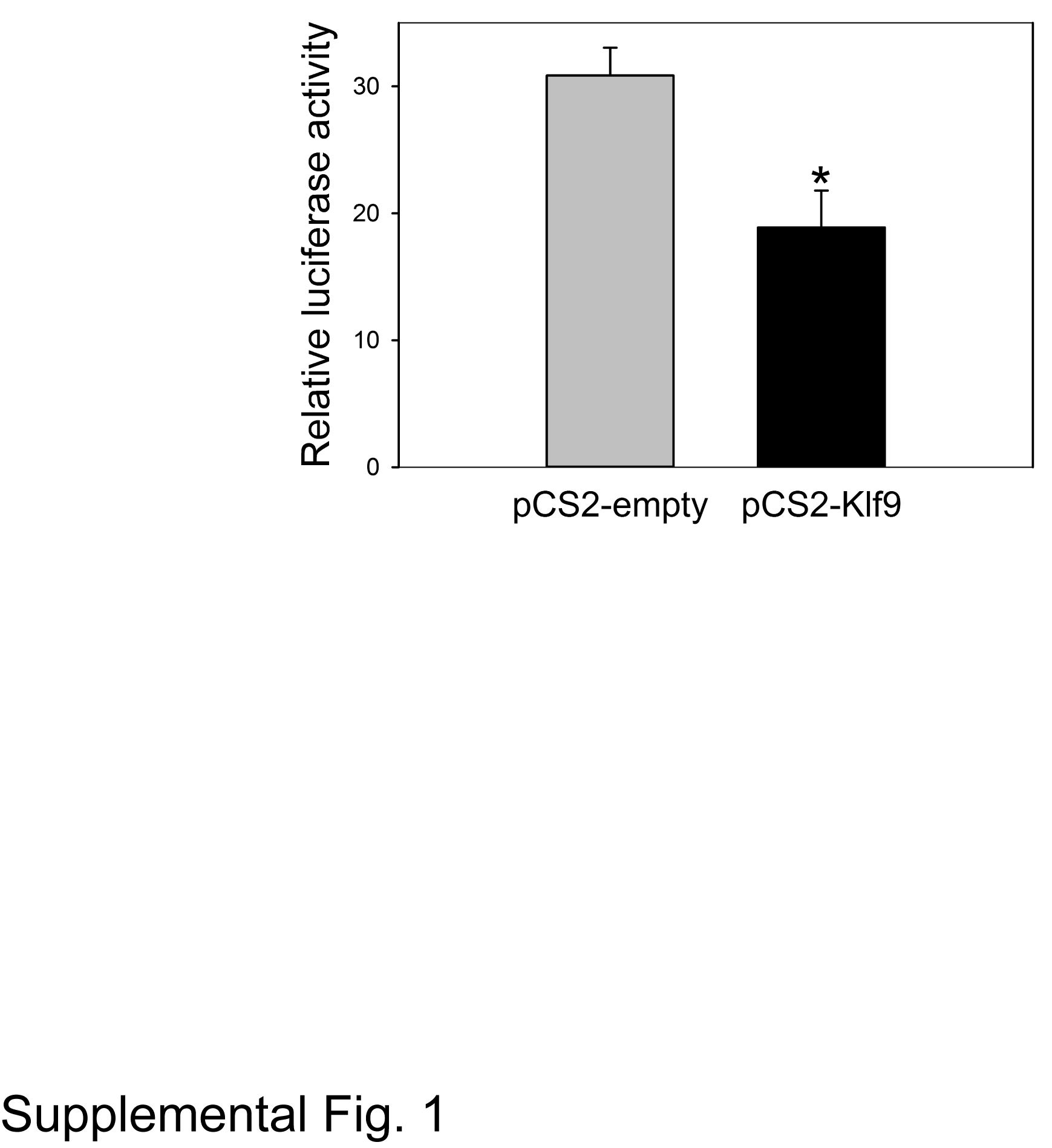

Supplement: Supplementary file 1 — Forced expression of Klf9 by transient transfection of HT22 cells reduced activity of a synthetic promoter containing three copies of the basic transcription element (BTE). (TIF 3252 kb) [file 12864_2017_3640_MOESM1_ESM.tif]

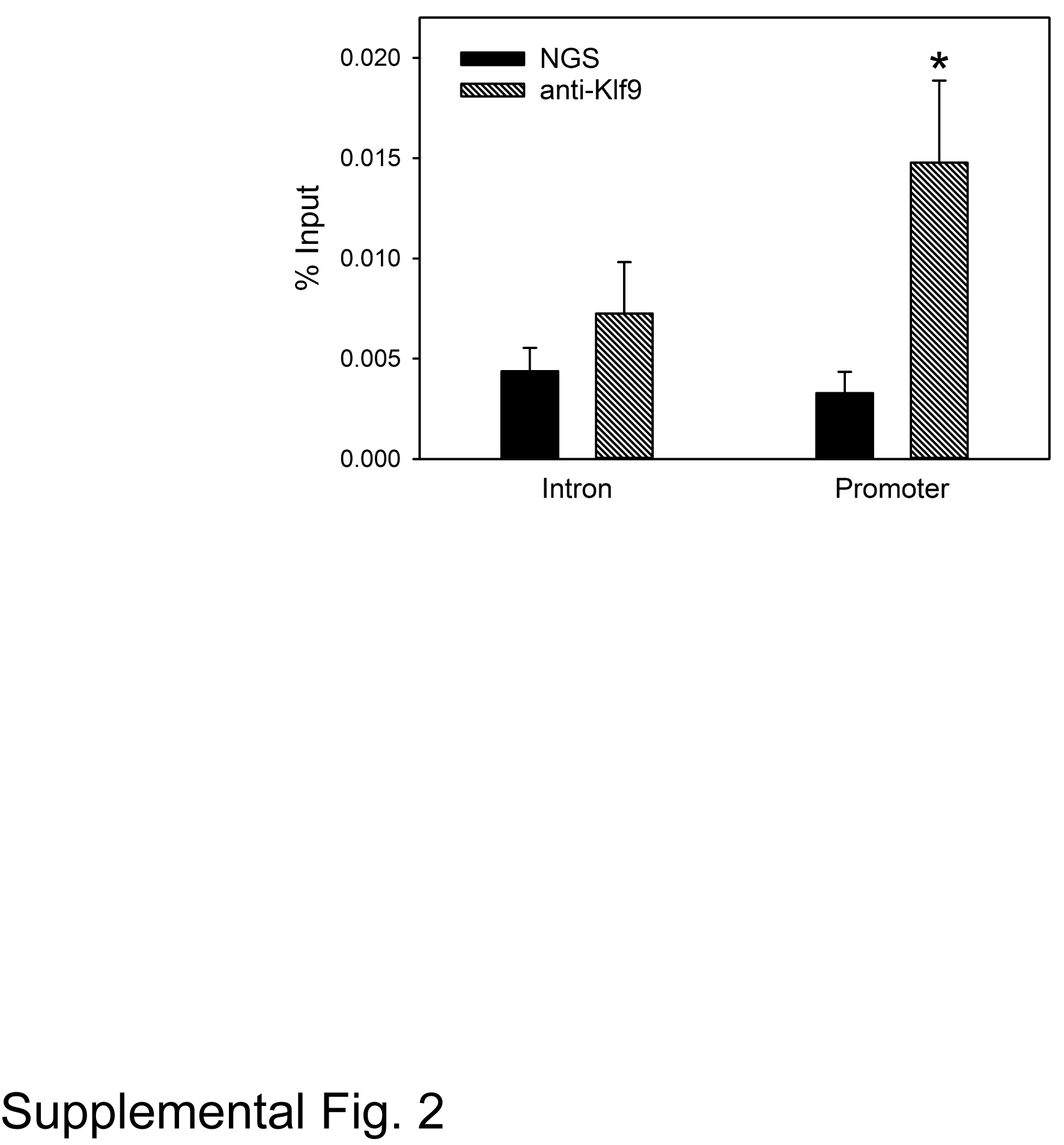

Supplement: Supplementary file 3 — Klf9 associates in chromatin at the region of the Klf13 promoter in HT22 cells. (TIF 3325 kb) [file 12864_2017_3640_MOESM3_ESM.tif]

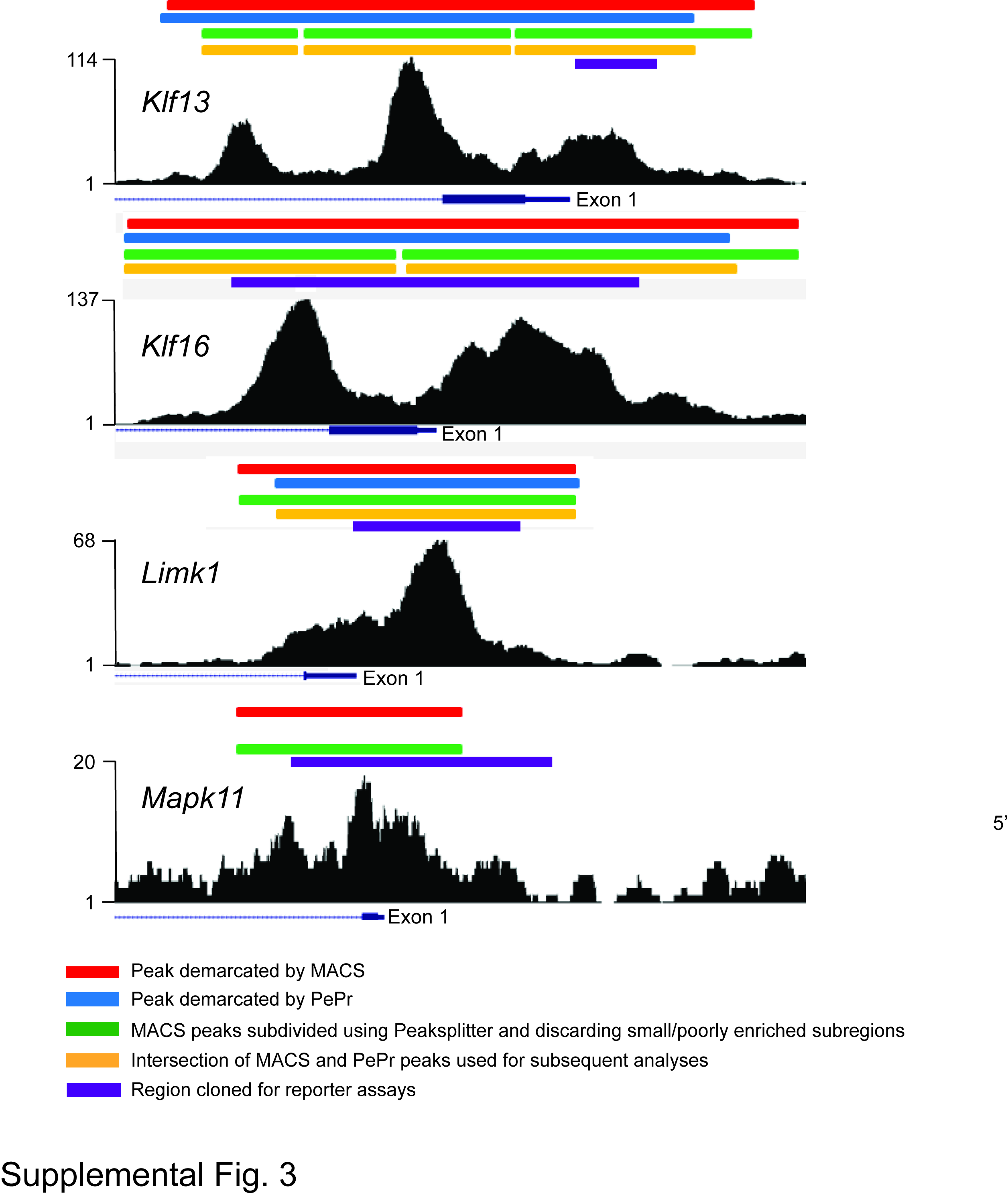

Supplement: Supplementary file 4 — Comparison of computational approaches used to identify regions of Klf9 association in chromatin across the genome of HT22 cells. (TIF 29857 kb) [file 12864_2017_3640_MOESM4_ESM.tif]

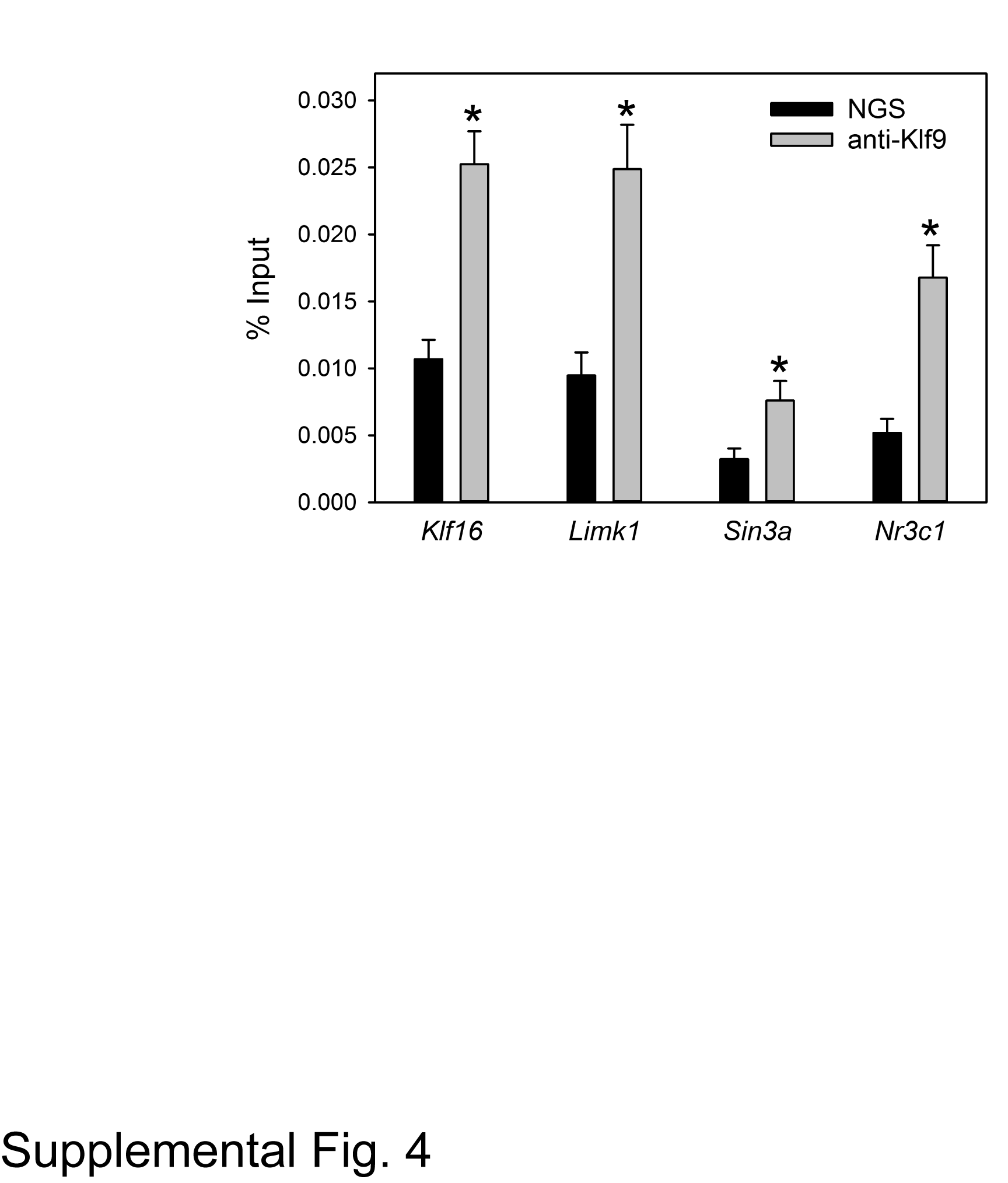

Supplement: Supplementary file 6 — Validation of regions of Klf9 association in chromatin in HT22 cells discovered by chromatin streptavidin precipitation sequencing, analyzed by targeted chromatin immunoprecipitation for Klf9. (TIF 3453 kb) [file 12864_2017_3640_MOESM6_ESM.tif]

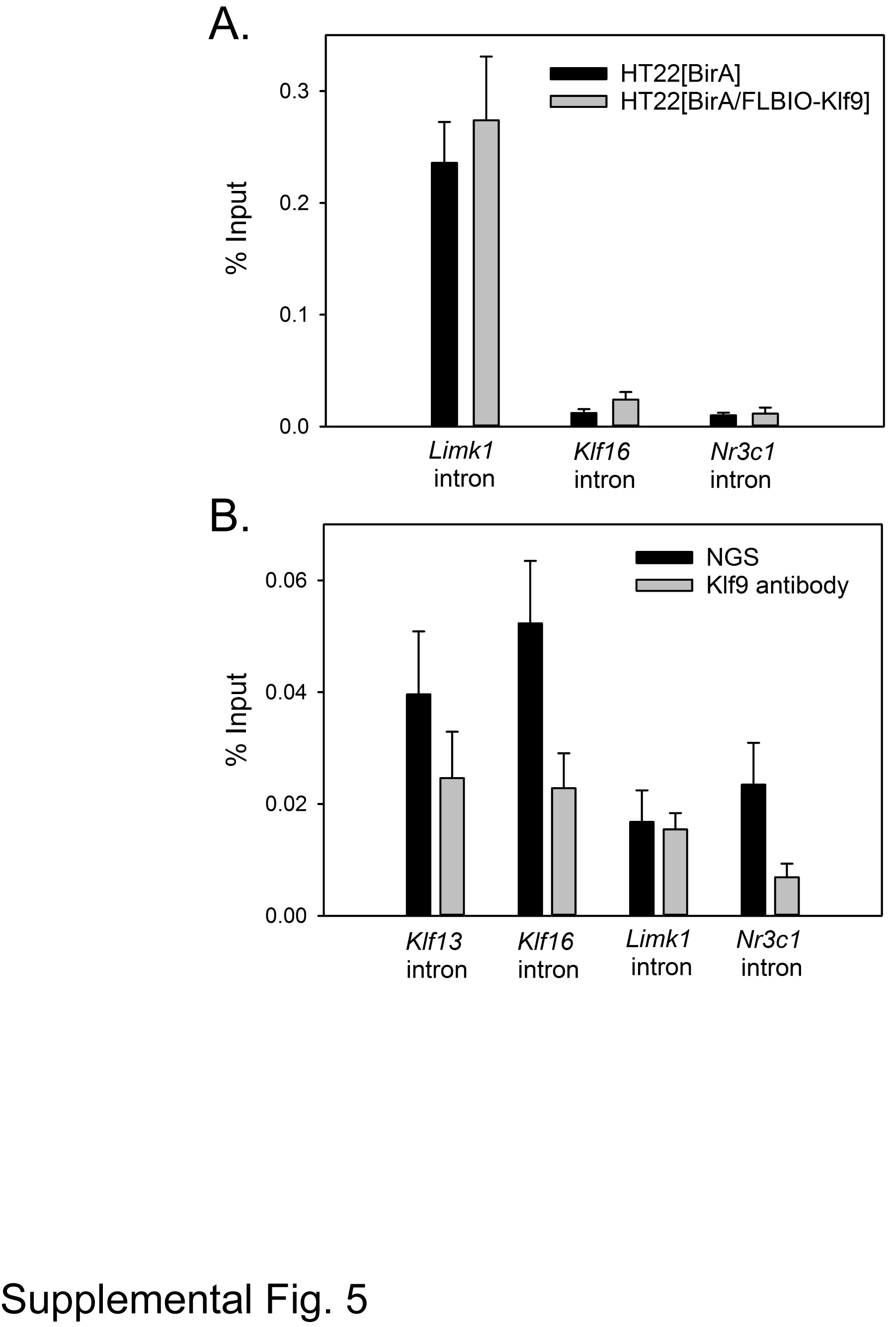

Supplement: Supplementary file 7 — Analysis of genomic regions in HT22 cells and mouse hippocampus that lacked Klf9 peaks by chromatin streptavidin precipitation (ChSP) sequencing. (TIF 4729 kb) [file 12864_2017_3640_MOESM7_ESM.tif]

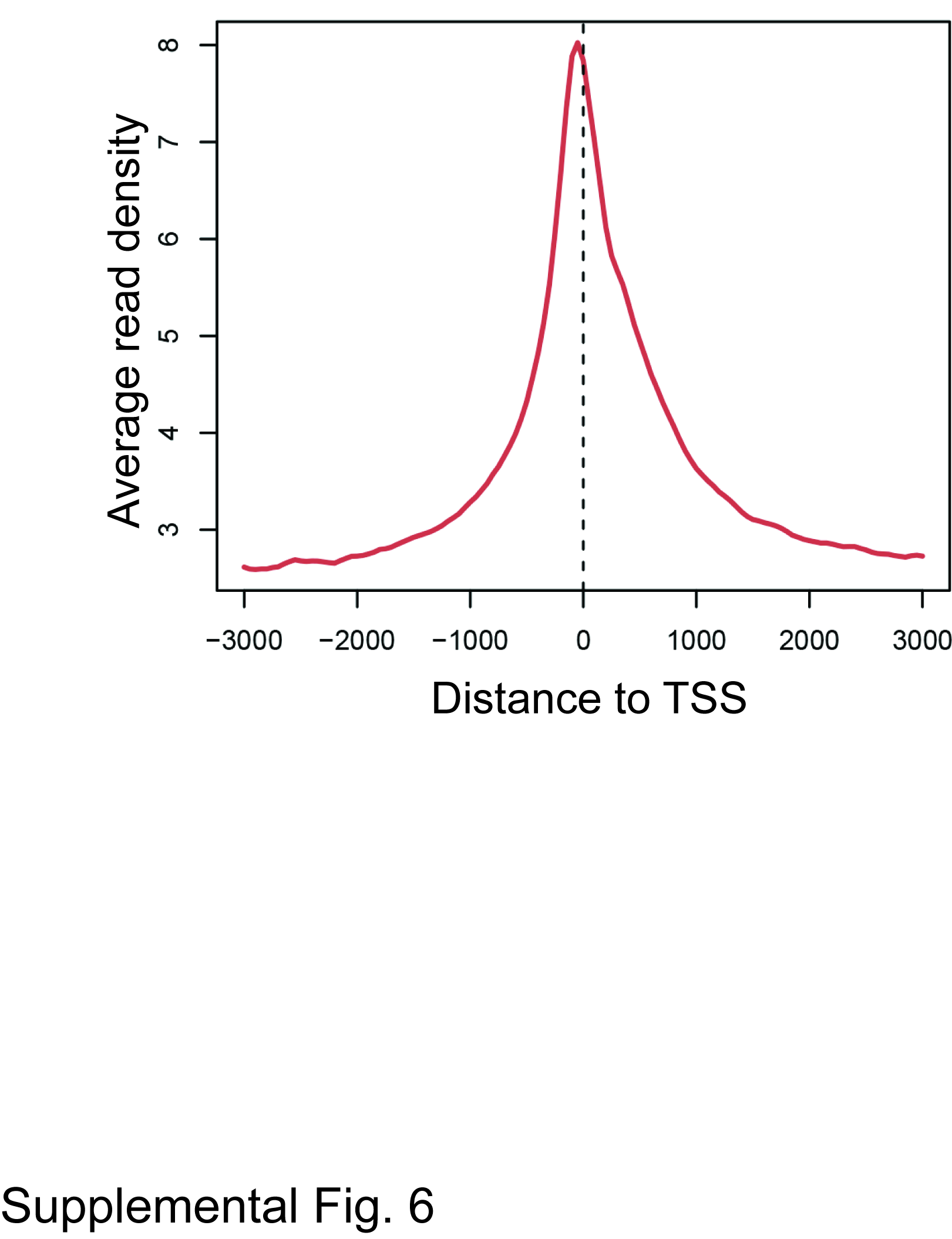

Supplement: Supplementary file 8 — Analysis of the distribution of mapped sequencing reads around transcription start sites (TSS) revealed a moderate bias towards regions immediately upstream of the TSSs. (TIF 13937 kb) [file 12864_2017_3640_MOESM8_ESM.tif]

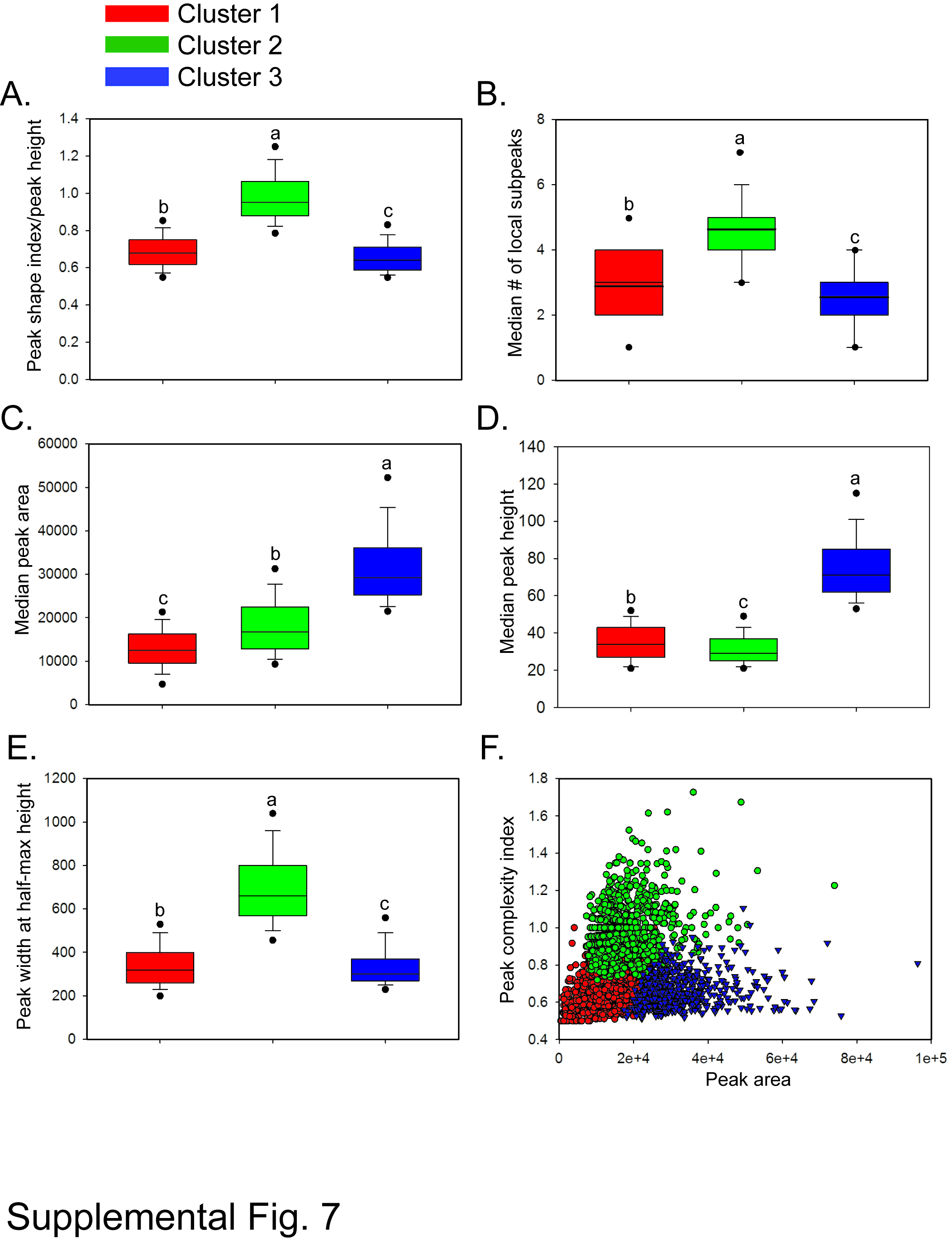

Supplement: Supplementary file 11 — Quantification of peak shape parameters from each cluster identified using the computer program SIC-ChIP. (TIF 23137 kb) [file 12864_2017_3640_MOESM11_ESM.tif]

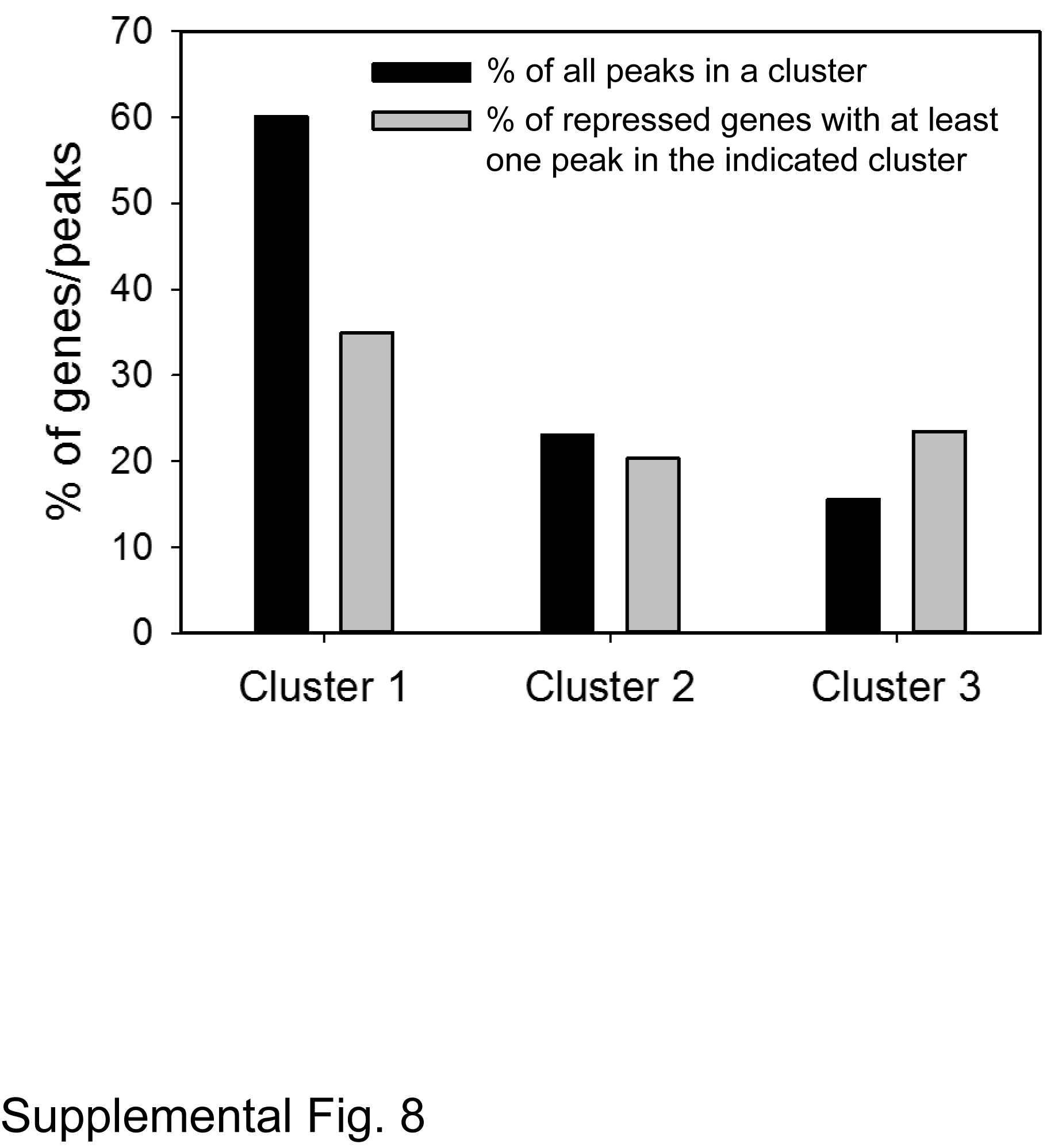

Supplement: Supplementary file 12 — A greater relative percentage of chromatin streptavidin precipitation (ChSP) sequencing peaks belonging to Clusters 2 and 3 are associated with genes repressed by Klf9 compared with peaks from Cluster 1. (TIF 3611 kb) [file 12864_2017_3640_MOESM12_ESM.tif]

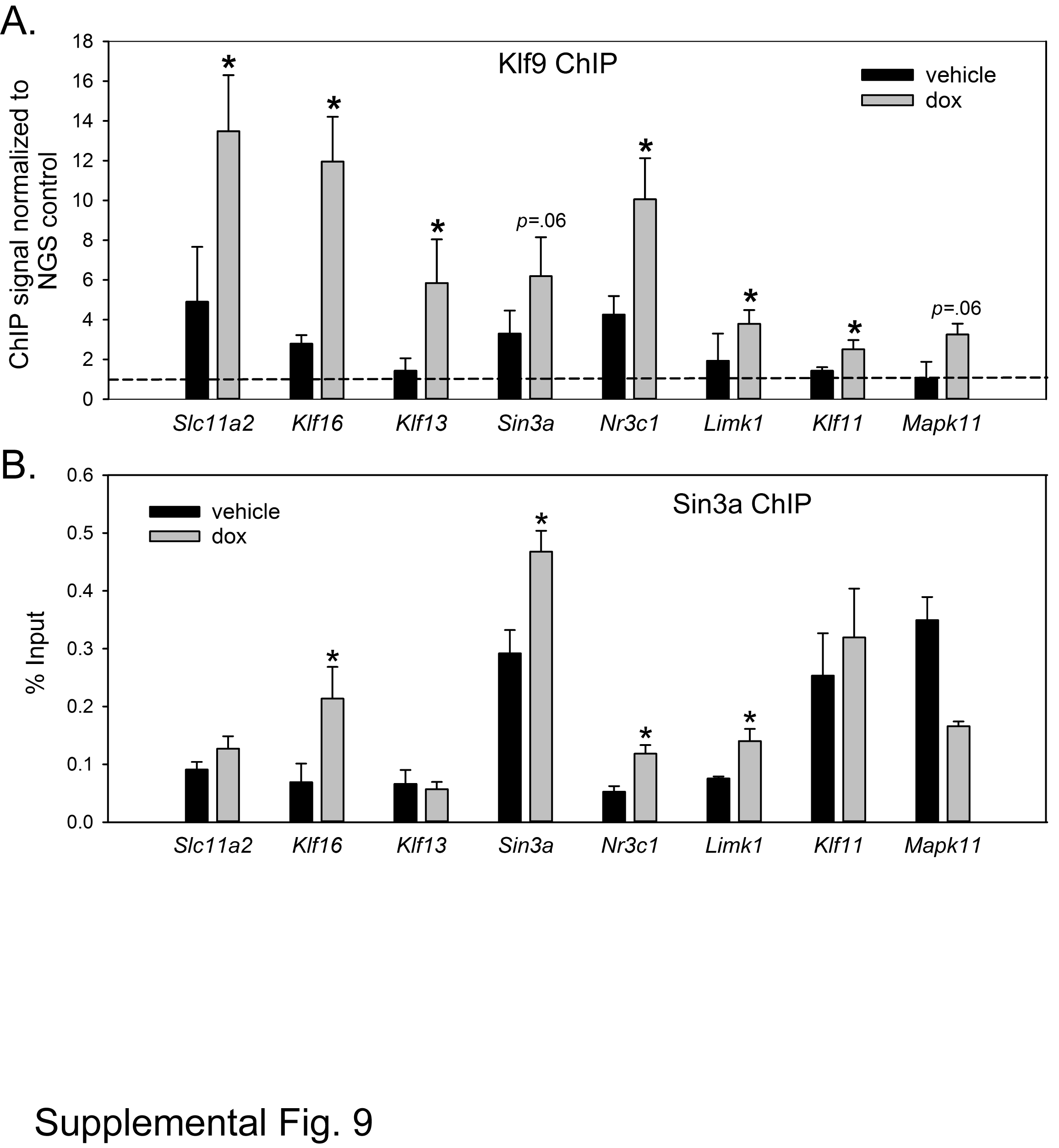

Supplement: Supplementary file 14 — Validation of Klf9 association in chromatin in HT22 cells with the 5′ flanking regions of genes identified by chromatin streptavidin precipitation sequencing. (TIF 5685 kb) [file 12864_2017_3640_MOESM14_ESM.tif]
