# Supplementary material for: The Krüppel-like factor 9 cistrome in mouse hippocampal neurons reveals predominant transcriptional repression via proximal promoter binding
Source: BMC Genomics. 2017 Apr 13;18:299. doi: 10.1186/s12864-017-3640-7 (PMC5390390; doi:10.1186/s12864-017-3640-7)
Supplement: Supplementary file 2 — List of all genes that were up- or down-regulated after eight hours of doxycycline treatment of HT22 [TR/TO-Klf9] cells. (DOCX 46 kb) [file 12864_2017_3640_MOESM2_ESM.docx]

**Supplemental Table 1:** List of all genes that were up- or down-regulated after eight hours of doxycycline treatment of HT22[TR/TO-Klf9] cells. The false discover rate (FDR)-adjusted *p* value cutoff was set to .005. The mRNA levels (counts) were evaluated by DESeq. Genes are ordered by log_2_ fold change (lowest to highest).

| Gene name | Counts (-Dox) | Counts (+Dox) | Fold change | Log_2_ Fold Change | *p* value | FDR-adjusted *p* value |
| --- | --- | --- | --- | --- | --- | --- |
| Klf13 | 582.35 | 268.62 | 0.46 | -1.12 | 8.09E-24 | 1.09E-19 |
| Rptoros | 89.31 | 43.32 | 0.48 | -1.04 | 3.38E-06 | 0.0005 |
| Gpr161 | 229.15 | 118.76 | 0.52 | -0.95 | 9.10E-07 | 0.000172 |
| Apc2 | 560.05 | 295.13 | 0.53 | -0.92 | 1.30E-16 | 6.99E-13 |
| Zfp704 | 229.9 | 123.87 | 0.54 | -0.89 | 3.87E-09 | 2.18E-06 |
| Arhgap39 | 256.48 | 139.04 | 0.54 | -0.88 | 1.06E-10 | 1.14E-07 |
| Armc7 | 321.41 | 175.72 | 0.55 | -0.87 | 9.70E-11 | 1.14E-07 |
| Mex3a | 816.98 | 452.47 | 0.55 | -0.85 | 2.40E-18 | 2.16E-14 |
| Wdfy2 | 252.68 | 140.48 | 0.56 | -0.85 | 1.23E-06 | 0.000214 |
| Nlgn2 | 1179.72 | 672.25 | 0.57 | -0.81 | 5.92E-18 | 4.00E-14 |
| 1700109K24Rik | 511.67 | 295.81 | 0.58 | -0.79 | 4.98E-05 | 0.003861 |
| Sft2d1 | 421.9 | 247.85 | 0.59 | -0.77 | 9.39E-09 | 4.62E-06 |
| Arap1 | 209.16 | 123.07 | 0.59 | -0.77 | 7.90E-07 | 0.000152 |
| Btbd9 | 169.23 | 99.39 | 0.59 | -0.77 | 5.10E-06 | 0.000684 |
| Plxna3 | 964.79 | 570.55 | 0.59 | -0.76 | 2.80E-16 | 1.26E-12 |
| Kcnh2 | 236.83 | 139.63 | 0.59 | -0.76 | 9.57E-07 | 0.000178 |
| Vav2 | 542.08 | 322.05 | 0.59 | -0.75 | 1.68E-12 | 3.24E-09 |
| Nadk2 | 323.48 | 192.45 | 0.59 | -0.75 | 6.18E-09 | 3.33E-06 |
| Lzts3 | 553.16 | 334.05 | 0.6 | -0.73 | 5.16E-11 | 6.63E-08 |
| Plxnd1 | 501.53 | 305.3 | 0.61 | -0.72 | 6.55E-09 | 3.46E-06 |
| Clip2 | 1243.77 | 758.98 | 0.61 | -0.71 | 5.24E-16 | 1.77E-12 |
| Lonrf1 | 398.49 | 243 | 0.61 | -0.71 | 3.49E-07 | 8.19E-05 |
| Zfp382 | 284.69 | 175.59 | 0.62 | -0.7 | 1.89E-07 | 5.00E-05 |
| Cul7 | 963.06 | 597.06 | 0.62 | -0.69 | 2.61E-08 | 1.07E-05 |
| Fchsd1 | 228.15 | 141.73 | 0.62 | -0.69 | 4.84E-06 | 0.000677 |
| Arhgap33 | 289.8 | 179.34 | 0.62 | -0.69 | 4.44E-05 | 0.003552 |
| Ttc28 | 784.61 | 490.42 | 0.63 | -0.68 | 5.38E-12 | 9.07E-09 |
| Gpsm1 | 529.14 | 330.54 | 0.62 | -0.68 | 9.58E-09 | 4.62E-06 |
| Armcx2 | 1453.15 | 915.63 | 0.63 | -0.67 | 2.30E-15 | 5.64E-12 |
| Klf16 | 743.47 | 465.7 | 0.63 | -0.67 | 7.79E-12 | 1.17E-08 |
| Rufy3 | 551.43 | 346.72 | 0.63 | -0.67 | 4.48E-10 | 3.53E-07 |
| Slc22a4 | 177.77 | 111.72 | 0.63 | -0.67 | 1.86E-05 | 0.001918 |
| Sgsm2 | 858.39 | 543.25 | 0.63 | -0.66 | 6.73E-12 | 1.07E-08 |
| Prune2 | 1318.1 | 835.09 | 0.63 | -0.66 | 9.96E-10 | 6.56E-07 |
| 4933428G20Rik | 166.66 | 105.67 | 0.63 | -0.66 | 6.14E-05 | 0.004479 |
| Nyap1 | 255.38 | 163.19 | 0.64 | -0.65 | 6.10E-06 | 0.000787 |
| Ctdspl | 733.62 | 470.93 | 0.64 | -0.64 | 8.74E-11 | 1.07E-07 |
| Prmt2 | 697.81 | 446.88 | 0.64 | -0.64 | 2.16E-10 | 1.88E-07 |
| Zbtb12 | 440.94 | 283.46 | 0.64 | -0.64 | 2.28E-08 | 9.48E-06 |
| Mdn1 | 2924.59 | 1907.07 | 0.65 | -0.62 | 3.32E-12 | 5.97E-09 |
| 2310003H01Rik | 781.94 | 508.14 | 0.65 | -0.62 | 1.05E-05 | 0.001245 |
| Six5 | 289.85 | 188.7 | 0.65 | -0.62 | 1.23E-05 | 0.001401 |
| Hhipl1 | 185.23 | 120.63 | 0.65 | -0.62 | 5.66E-05 | 0.004266 |
| Limk1 | 869.59 | 564.76 | 0.65 | -0.62 | 5.83E-05 | 0.004319 |
| Micall1 | 1524.69 | 997.53 | 0.65 | -0.61 | 1.84E-13 | 4.14E-10 |
| Nagk | 273.1 | 178.72 | 0.65 | -0.61 | 1.42E-05 | 0.00155 |
| Tiam1 | 841.49 | 554.16 | 0.66 | -0.6 | 2.26E-10 | 1.91E-07 |
| Rapgef3 | 292.14 | 193 | 0.66 | -0.6 | 3.21E-06 | 0.000483 |
| Ubr4 | 6234.97 | 4120.79 | 0.66 | -0.6 | 2.72E-05 | 0.002521 |
| Plekhg2 | 824.4 | 548.12 | 0.66 | -0.59 | 9.86E-10 | 6.56E-07 |
| Kmt2d | 4234.24 | 2815.2 | 0.66 | -0.59 | 2.94E-08 | 1.18E-05 |
| Cdh24 | 468.74 | 310.97 | 0.66 | -0.59 | 2.13E-07 | 5.31E-05 |
| Syne1 | 939.64 | 624.41 | 0.66 | -0.59 | 1.25E-05 | 0.001421 |
| Kmt2a | 3226.22 | 2157.98 | 0.67 | -0.58 | 7.39E-10 | 5.54E-07 |
| Ankrd52 | 2896.22 | 1933.77 | 0.67 | -0.58 | 1.61E-07 | 4.56E-05 |
| Setd1b | 2097.16 | 1417.02 | 0.68 | -0.57 | 5.65E-13 | 1.17E-09 |
| Mapk11 | 362.26 | 243.56 | 0.67 | -0.57 | 2.41E-06 | 0.000384 |
| Ccdc120 | 276.27 | 185.63 | 0.67 | -0.57 | 2.91E-05 | 0.002646 |
| Slc27a1 | 431.33 | 290.24 | 0.67 | -0.57 | 7.09E-05 | 0.004953 |
| Cand2 | 844.89 | 572.34 | 0.68 | -0.56 | 2.23E-09 | 1.40E-06 |
| Tead4 | 718.96 | 487.62 | 0.68 | -0.56 | 3.22E-09 | 1.85E-06 |
| Fam13b | 1765.62 | 1198.29 | 0.68 | -0.56 | 6.81E-07 | 0.000141 |
| Arhgap23 | 1128.48 | 769.23 | 0.68 | -0.55 | 4.58E-10 | 3.53E-07 |
| Klc2 | 830.87 | 565.79 | 0.68 | -0.55 | 8.82E-09 | 4.49E-06 |
| Slc35e3 | 639.62 | 437.28 | 0.68 | -0.55 | 1.07E-08 | 4.97E-06 |
| Map4k3 | 727.89 | 498.57 | 0.68 | -0.55 | 1.46E-08 | 6.13E-06 |
| Usp28 | 545.88 | 373.07 | 0.68 | -0.55 | 2.37E-07 | 5.87E-05 |
| D11Wsu47e | 453.13 | 308.83 | 0.68 | -0.55 | 6.54E-07 | 0.000137 |
| Aff1 | 1827.98 | 1245.77 | 0.68 | -0.55 | 1.91E-06 | 0.000316 |
| Plcd1 | 328.15 | 223.6 | 0.68 | -0.55 | 2.34E-05 | 0.002277 |
| Dclk2 | 636.63 | 436.72 | 0.69 | -0.54 | 1.38E-08 | 5.92E-06 |
| Phf13 | 707.16 | 487.13 | 0.69 | -0.54 | 5.13E-07 | 0.00011 |
| Fdps | 274.91 | 189.58 | 0.69 | -0.54 | 7.07E-05 | 0.004953 |
| Pak4 | 779.66 | 539.42 | 0.69 | -0.53 | 6.98E-07 | 0.000143 |
| Arl10 | 295.46 | 205.25 | 0.69 | -0.53 | 4.21E-05 | 0.003398 |
| Gigyf1 | 1283.82 | 897.85 | 0.7 | -0.52 | 2.44E-09 | 1.47E-06 |
| Sept5 | 1054.54 | 737.57 | 0.7 | -0.52 | 3.54E-08 | 1.33E-05 |
| Pdia5 | 2791.31 | 1943.49 | 0.7 | -0.52 | 5.77E-06 | 0.000756 |
| Rhbdf2 | 358.85 | 249.88 | 0.7 | -0.52 | 1.59E-05 | 0.00167 |
| Tbc1d22a | 628.91 | 442 | 0.7 | -0.51 | 5.68E-07 | 0.000121 |
| Ppp1r13b | 435.72 | 305.63 | 0.7 | -0.51 | 1.30E-05 | 0.001453 |
| Pou6f1 | 310.16 | 217.17 | 0.7 | -0.51 | 5.26E-05 | 0.003998 |
| D630045J12Rik | 800.71 | 566.58 | 0.71 | -0.5 | 6.17E-08 | 2.03E-05 |
| Tbc1d24 | 787.23 | 557.19 | 0.71 | -0.5 | 6.96E-08 | 2.24E-05 |
| Fbxl19 | 865.74 | 613.89 | 0.71 | -0.5 | 2.47E-07 | 6.04E-05 |
| Bcl9 | 573.22 | 404.79 | 0.71 | -0.5 | 1.06E-06 | 0.000195 |
| Map1b | 8197.68 | 5805.43 | 0.71 | -0.5 | 1.32E-06 | 0.000228 |
| Acvr1b | 448.82 | 317.95 | 0.71 | -0.5 | 2.45E-05 | 0.002347 |
| Cchcr1 | 1105.58 | 785.5 | 0.71 | -0.49 | 3.37E-08 | 1.33E-05 |
| Ptprf | 3296.48 | 2344.73 | 0.71 | -0.49 | 9.25E-08 | 2.90E-05 |
| Trio | 3147.72 | 2236.05 | 0.71 | -0.49 | 8.48E-06 | 0.001051 |
| Mroh1 | 743.98 | 530.56 | 0.71 | -0.49 | 1.51E-05 | 0.001595 |
| Zhx3 | 348.91 | 247.65 | 0.71 | -0.49 | 3.62E-05 | 0.002998 |
| Pvrl1 | 615 | 441.23 | 0.72 | -0.48 | 4.04E-06 | 0.000584 |
| Ttc3 | 2951.62 | 2129.44 | 0.72 | -0.47 | 2.76E-10 | 2.25E-07 |
| Tsc1 | 964.34 | 695.16 | 0.72 | -0.47 | 1.37E-07 | 4.11E-05 |
| 9930021J03Rik | 912.98 | 657.64 | 0.72 | -0.47 | 1.63E-07 | 4.58E-05 |
| Tns3 | 2235.36 | 1626.02 | 0.73 | -0.46 | 8.70E-10 | 6.34E-07 |
| Nav2 | 2142.37 | 1552.91 | 0.72 | -0.46 | 1.93E-09 | 1.24E-06 |
| Hivep1 | 1104.92 | 802.8 | 0.73 | -0.46 | 9.35E-08 | 2.90E-05 |
| Dysf | 718.29 | 521.76 | 0.73 | -0.46 | 7.12E-06 | 0.000898 |
| Ahdc1 | 749.58 | 544.69 | 0.73 | -0.46 | 1.22E-05 | 0.001401 |
| Arid3a | 531.99 | 387.6 | 0.73 | -0.46 | 1.26E-05 | 0.001421 |
| Rab11b | 486.44 | 354.65 | 0.73 | -0.46 | 1.48E-05 | 0.001593 |
| Nuak1 | 380.42 | 276.13 | 0.73 | -0.46 | 6.38E-05 | 0.0046 |
| Zfp516 | 1068.67 | 783.39 | 0.73 | -0.45 | 2.70E-07 | 6.45E-05 |
| Myo1d | 660.09 | 482.97 | 0.73 | -0.45 | 2.92E-05 | 0.002648 |
| Smurf1 | 1956.61 | 1446.77 | 0.74 | -0.44 | 3.56E-08 | 1.33E-05 |
| Pogk | 996.68 | 736.45 | 0.74 | -0.44 | 1.39E-06 | 0.000238 |
| Tyro3 | 919.17 | 678.93 | 0.74 | -0.44 | 2.54E-06 | 0.000399 |
| Cdc42ep4 | 659.55 | 486.38 | 0.74 | -0.44 | 8.34E-06 | 0.001042 |
| Exo5 | 700.07 | 516.02 | 0.74 | -0.44 | 1.10E-05 | 0.001283 |
| Ccdc136 | 563.42 | 414.67 | 0.74 | -0.44 | 1.30E-05 | 0.001453 |
| Dpy19l3 | 581.09 | 428.23 | 0.74 | -0.44 | 1.54E-05 | 0.001624 |
| Hspg2 | 10365.48 | 7620.63 | 0.74 | -0.44 | 2.45E-05 | 0.002347 |
| Camk2g | 1718.65 | 1276.45 | 0.74 | -0.43 | 1.69E-07 | 4.61E-05 |
| Fnip2 | 1461.01 | 1082.95 | 0.74 | -0.43 | 7.17E-07 | 0.000143 |
| Cspg4 | 4394.02 | 3252.35 | 0.74 | -0.43 | 9.18E-07 | 0.000172 |
| Golga4 | 2370.98 | 1756.86 | 0.74 | -0.43 | 6.36E-06 | 0.000817 |
| Dock6 | 823.14 | 612.7 | 0.74 | -0.43 | 8.51E-06 | 0.001051 |
| Cmtm3 | 768.58 | 569.26 | 0.74 | -0.43 | 1.07E-05 | 0.001254 |
| Pcnxl3 | 2076.16 | 1547.64 | 0.75 | -0.42 | 3.49E-08 | 1.33E-05 |
| Rab11fip5 | 1889.88 | 1411.81 | 0.75 | -0.42 | 2.06E-07 | 5.19E-05 |
| Arhgap32 | 1321.88 | 988.54 | 0.75 | -0.42 | 7.62E-07 | 0.000148 |
| Zfp568 | 909.9 | 678.75 | 0.75 | -0.42 | 2.42E-06 | 0.000384 |
| 4930402H24Rik | 832.01 | 623.33 | 0.75 | -0.42 | 1.25E-05 | 0.001419 |
| Il17ra | 649.76 | 484.36 | 0.75 | -0.42 | 2.45E-05 | 0.002347 |
| Rnf24 | 639.47 | 477.47 | 0.75 | -0.42 | 3.31E-05 | 0.002879 |
| S1pr2 | 534.44 | 399.84 | 0.75 | -0.42 | 4.86E-05 | 0.003817 |
| Ppp4r1l-ps | 512.17 | 383.54 | 0.75 | -0.42 | 6.66E-05 | 0.004727 |
| Trrap | 5032.89 | 3781.28 | 0.75 | -0.41 | 4.51E-09 | 2.48E-06 |
| Cic | 3293.37 | 2479.23 | 0.75 | -0.41 | 5.49E-08 | 1.98E-05 |
| Spaca6 | 2058.63 | 1551.43 | 0.75 | -0.41 | 1.57E-07 | 4.50E-05 |
| Plxna1 | 5069.23 | 3807.86 | 0.75 | -0.41 | 2.02E-07 | 5.19E-05 |
| Slc4a2 | 1385.38 | 1040.08 | 0.75 | -0.41 | 1.05E-06 | 0.000194 |
| Mex3d | 993.97 | 748.45 | 0.75 | -0.41 | 4.86E-06 | 0.000677 |
| Gtf2ird1 | 724.79 | 545.43 | 0.75 | -0.41 | 2.82E-05 | 0.002584 |
| Sh3pxd2b | 2125.81 | 1608.7 | 0.76 | -0.4 | 2.00E-07 | 5.19E-05 |
| Megf8 | 2698.24 | 2045.88 | 0.76 | -0.4 | 2.85E-07 | 6.76E-05 |
| Arhgef40 | 1930.56 | 1464.74 | 0.76 | -0.4 | 4.15E-07 | 9.41E-05 |
| Tmem63b | 1472.54 | 1118.99 | 0.76 | -0.4 | 2.29E-06 | 0.000373 |
| Gfod1 | 1054.68 | 800.11 | 0.76 | -0.4 | 4.62E-06 | 0.000659 |
| Fnbp1 | 1109.76 | 840.81 | 0.76 | -0.4 | 5.17E-06 | 0.000691 |
| Bahd1 | 914.83 | 693.49 | 0.76 | -0.4 | 8.66E-06 | 0.001062 |
| Anks1 | 832.68 | 632.74 | 0.76 | -0.4 | 1.91E-05 | 0.001941 |
| Gmip | 804.84 | 610.99 | 0.76 | -0.4 | 4.00E-05 | 0.003283 |
| Arhgef25 | 753.93 | 571.52 | 0.76 | -0.4 | 5.84E-05 | 0.004319 |
| Heatr5a | 1699.76 | 1292.05 | 0.76 | -0.4 | 6.46E-05 | 0.004646 |
| Rai1 | 2575.88 | 1969.95 | 0.76 | -0.39 | 1.75E-07 | 4.73E-05 |
| Cdr2l | 2663.14 | 2039.03 | 0.77 | -0.39 | 1.87E-07 | 4.98E-05 |
| Myo1b | 1380.72 | 1050.1 | 0.76 | -0.39 | 1.12E-06 | 0.000198 |
| Dip2a | 1328.93 | 1011.68 | 0.76 | -0.39 | 2.16E-06 | 0.000353 |
| Map3k4 | 1260.02 | 963.79 | 0.76 | -0.39 | 5.00E-06 | 0.000684 |
| Csk | 1124.07 | 855.32 | 0.76 | -0.39 | 1.41E-05 | 0.00155 |
| Kdm2b | 862.06 | 656.98 | 0.76 | -0.39 | 2.04E-05 | 0.002057 |
| Samd4b | 2274.28 | 1736.48 | 0.76 | -0.39 | 5.84E-05 | 0.004319 |
| Agrn | 2210.99 | 1693.98 | 0.77 | -0.38 | 4.29E-07 | 9.65E-05 |
| Ehbp1l1 | 2545.01 | 1961.43 | 0.77 | -0.38 | 4.62E-07 | 0.000101 |
| Clcf1 | 1715.31 | 1321.67 | 0.77 | -0.38 | 1.74E-06 | 0.000289 |
| Diap1 | 1376.8 | 1056.28 | 0.77 | -0.38 | 2.67E-06 | 0.000417 |
| Ptgfrn | 1339.71 | 1028.53 | 0.77 | -0.38 | 6.06E-06 | 0.000786 |
| Tns1 | 1124.28 | 861.36 | 0.77 | -0.38 | 1.42E-05 | 0.00155 |
| Ttbk2 | 1026.26 | 790.68 | 0.77 | -0.38 | 1.51E-05 | 0.001595 |
| Pkd1 | 3665.83 | 2821.96 | 0.77 | -0.38 | 2.83E-05 | 0.002584 |
| Wdr91 | 861.39 | 662.73 | 0.77 | -0.38 | 3.19E-05 | 0.002805 |
| Ttyh3 | 994.87 | 764.22 | 0.77 | -0.38 | 3.30E-05 | 0.002879 |
| Usp42 | 771.58 | 593.71 | 0.77 | -0.38 | 6.10E-05 | 0.004463 |
| Uba6 | 1341.38 | 1029.92 | 0.77 | -0.38 | 6.30E-05 | 0.004555 |
| Nfe2l1 | 4769.02 | 3685.87 | 0.77 | -0.37 | 1.96E-07 | 5.13E-05 |
| Ilf3 | 2137.27 | 1650.23 | 0.77 | -0.37 | 1.70E-06 | 0.000286 |
| Prr12 | 1535.64 | 1186.24 | 0.77 | -0.37 | 8.77E-06 | 0.001071 |
| Hip1 | 2007.71 | 1557.28 | 0.78 | -0.37 | 5.18E-05 | 0.003957 |
| Tmem184b | 2386.03 | 1859.37 | 0.78 | -0.36 | 4.86E-06 | 0.000677 |
| C77080 | 1687.38 | 1316.12 | 0.78 | -0.36 | 1.43E-05 | 0.001551 |
| Ralgapa2 | 1025.39 | 797.72 | 0.78 | -0.36 | 2.64E-05 | 0.00247 |
| Mkl2 | 1195.26 | 930.95 | 0.78 | -0.36 | 2.63E-05 | 0.00247 |
| Smarcc1 | 2567.54 | 2012.4 | 0.78 | -0.35 | 2.01E-06 | 0.00033 |
| Fnbp1l | 2482.54 | 1944.63 | 0.78 | -0.35 | 3.15E-06 | 0.000482 |
| Slc39a6 | 2310.57 | 1813.73 | 0.78 | -0.35 | 6.71E-06 | 0.000854 |
| Elf4 | 1946.41 | 1528.85 | 0.79 | -0.35 | 9.27E-06 | 0.001122 |
| Gdi1 | 1429.07 | 1117.77 | 0.78 | -0.35 | 3.53E-05 | 0.002943 |
| Ptprs | 5608.26 | 4418.13 | 0.79 | -0.34 | 7.12E-07 | 0.000143 |
| Ep400 | 4324.76 | 3406.28 | 0.79 | -0.34 | 8.96E-07 | 0.00017 |
| Sh3pxd2a | 4879.82 | 3866.05 | 0.79 | -0.34 | 5.73E-06 | 0.000755 |
| Spen | 2085.93 | 1644.27 | 0.79 | -0.34 | 5.81E-06 | 0.000757 |
| Tnks1bp1 | 2924.67 | 2316.75 | 0.79 | -0.34 | 6.50E-06 | 0.000831 |
| Scrib | 2862.22 | 2266.46 | 0.79 | -0.34 | 9.10E-06 | 0.001106 |
| Tet3 | 2318.83 | 1835.88 | 0.79 | -0.34 | 2.11E-05 | 0.002109 |
| Smg6 | 1397.98 | 1101.28 | 0.79 | -0.34 | 3.05E-05 | 0.00274 |
| Slc9a6 | 1111.85 | 875.46 | 0.79 | -0.34 | 4.20E-05 | 0.003398 |
| Epn2 | 1290.34 | 1020.65 | 0.79 | -0.34 | 4.68E-05 | 0.003704 |
| Mboat7 | 1352.47 | 1067.86 | 0.79 | -0.34 | 4.97E-05 | 0.003861 |
| Myo10 | 3227.01 | 2559.76 | 0.79 | -0.33 | 2.70E-06 | 0.000418 |
| Mrps6 | 3986.43 | 3173.69 | 0.8 | -0.33 | 3.44E-06 | 0.0005 |
| Sgpl1 | 2358.48 | 1872.76 | 0.79 | -0.33 | 1.46E-05 | 0.001572 |
| Uhrf1bp1 | 1828.19 | 1451.84 | 0.79 | -0.33 | 1.69E-05 | 0.00176 |
| Lphn1 | 1922.75 | 1533.32 | 0.8 | -0.33 | 2.70E-05 | 0.002521 |
| Chd3 | 2086.5 | 1663 | 0.8 | -0.33 | 2.72E-05 | 0.002521 |
| Rab11fip3 | 1943.49 | 1547.46 | 0.8 | -0.33 | 2.76E-05 | 0.00254 |
| Pcnx | 1625.56 | 1290.32 | 0.79 | -0.33 | 3.29E-05 | 0.002878 |
| Zfp609 | 1535.44 | 1219.77 | 0.79 | -0.33 | 3.35E-05 | 0.002893 |
| Kalrn | 1368.59 | 1085.68 | 0.79 | -0.33 | 4.89E-05 | 0.003821 |
| Gpi1 | 1375.02 | 1093.14 | 0.8 | -0.33 | 5.82E-05 | 0.004319 |
| Kdm3b | 1314.47 | 1044.47 | 0.79 | -0.33 | 6.23E-05 | 0.004532 |
| Git1 | 2076.15 | 1655.35 | 0.8 | -0.33 | 6.68E-05 | 0.004728 |
| Zmiz1 | 2612.87 | 2097.01 | 0.8 | -0.32 | 2.57E-05 | 0.002425 |
| Dlg5 | 2279.59 | 1824.22 | 0.8 | -0.32 | 3.08E-05 | 0.002751 |
| Foxj3 | 1636.93 | 1308.69 | 0.8 | -0.32 | 4.87E-05 | 0.003817 |
| Itpr3 | 4260.49 | 3428.16 | 0.8 | -0.31 | 8.53E-06 | 0.001051 |
| Prrc2b | 5819.24 | 4691.89 | 0.81 | -0.31 | 1.06E-05 | 0.001245 |
| Inppl1 | 2626 | 2121.24 | 0.81 | -0.31 | 5.12E-05 | 0.003923 |
| Arhgef17 | 2000.88 | 1617.35 | 0.81 | -0.31 | 5.70E-05 | 0.004281 |
| Flii | 2515.62 | 2030.03 | 0.81 | -0.31 | 6.10E-05 | 0.004463 |
| Sept8 | 4123.37 | 3359.59 | 0.81 | -0.3 | 5.06E-05 | 0.003891 |
| Wasf2 | 1970.51 | 1599.98 | 0.81 | -0.3 | 6.90E-05 | 0.004868 |
| Numa1 | 3741.98 | 3051.79 | 0.82 | -0.29 | 3.33E-05 | 0.002889 |
| Srebf2 | 4618.89 | 3776.26 | 0.82 | -0.29 | 4.58E-05 | 0.003652 |
| Ncapg | 2319.03 | 2895.29 | 1.25 | 0.32 | 2.84E-05 | 0.002584 |
| Mrpl15 | 973.97 | 1226.22 | 1.26 | 0.33 | 6.56E-05 | 0.004683 |
| Cdc20b | 897.09 | 1154.09 | 1.29 | 0.36 | 3.40E-05 | 0.002902 |
| Gpx8 | 883.46 | 1142.56 | 1.29 | 0.37 | 2.36E-05 | 0.002292 |
| Ostm1 | 680.79 | 885.23 | 1.3 | 0.38 | 5.21E-05 | 0.00397 |
| Kdelc1 | 833.95 | 1089.48 | 1.31 | 0.39 | 1.36E-05 | 0.001508 |
| Ndufaf4 | 474.8 | 623.84 | 1.31 | 0.39 | 5.83E-05 | 0.004319 |
| Pnrc2 | 657.33 | 870.02 | 1.32 | 0.4 | 1.19E-05 | 0.001373 |
| Spc25 | 609.05 | 811.79 | 1.33 | 0.41 | 1.01E-05 | 0.001211 |
| Cetn2 | 543.73 | 723.3 | 1.33 | 0.41 | 2.01E-05 | 0.002044 |
| Tex9 | 510.19 | 677.91 | 1.33 | 0.41 | 3.74E-05 | 0.003088 |
| Frk | 377.63 | 508.17 | 1.35 | 0.43 | 4.21E-05 | 0.003398 |
| 1810043G02Rik | 331.27 | 456.46 | 1.38 | 0.46 | 1.70E-05 | 0.001767 |
| 4930430F08Rik | 329.75 | 455.45 | 1.38 | 0.47 | 2.37E-05 | 0.002296 |
| 2610301B20Rik | 259.96 | 361.71 | 1.39 | 0.48 | 5.71E-05 | 0.004281 |
| Tctn2 | 295.57 | 414.24 | 1.4 | 0.49 | 3.77E-05 | 0.003098 |
| Arxes2 | 349.13 | 495.6 | 1.42 | 0.51 | 3.39E-06 | 0.0005 |
| Mitd1 | 321.81 | 459.74 | 1.43 | 0.51 | 4.99E-06 | 0.000684 |
| Rilpl2 | 291.08 | 413.73 | 1.42 | 0.51 | 7.82E-06 | 0.000981 |
| Zfp930 | 438.22 | 629.71 | 1.44 | 0.52 | 6.12E-07 | 0.000129 |
| Cdkn3 | 192.32 | 283.17 | 1.47 | 0.56 | 4.05E-05 | 0.003313 |
