# Supplementary material for: The Krüppel-like factor 9 cistrome in mouse hippocampal neurons reveals predominant transcriptional repression via proximal promoter binding
Source: BMC Genomics. 2017 Apr 13;18:299. doi: 10.1186/s12864-017-3640-7 (PMC5390390; doi:10.1186/s12864-017-3640-7)
Supplement: Supplementary file 9 — List of all Sp/Klf sequences identified as enriched above background in Klf9 ChSP peaks in HT22 [BirA/FLBIO-Klf9] cells. (DOCX 112 kb) [file 12864_2017_3640_MOESM9_ESM.docx]

| **Supplemental Table 3:** List of all Sp/Klf sequences identified as enriched above background in Klf9 ChSP peaks in HT22[BirA/FLBIO-Klf9] cells. The sequences are displayed as position weight matrices showing relative frequency of each nucleotide at each position. | | | |
| --- | --- | --- | --- |
| Motif | Enrichment *p* value | % of peaks containing motif | % of background sequences containing motif |
| 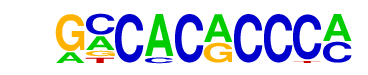 | 1*10^-537^ | 70.37 | 29.68 |
| 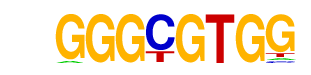 | 1*10^-353^ | 69.71 | 36.26 |
| 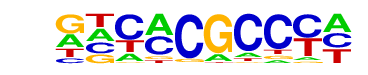 | 1*10^-334^ | 74.92 | 42.39 |
| 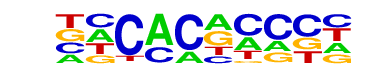 | 1*10^-294^ | 63.11 | 32.80 |
| 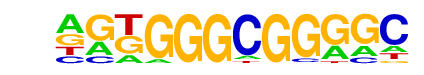 | 1*10^-151^ | 61.29 | 39.31 |
| 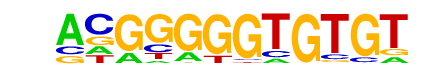 | 1*10^-107^ | 19.73 | 7.94 |
| 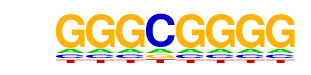 | 1*10^-84^ | 54.43 | 38.09 |
| 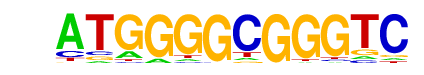 | 1*10^-71^ | 22 | 11.35 |
| 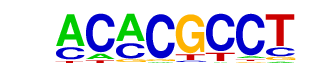 | 1*10^-51^ | 47.68 | 35.14 |
| 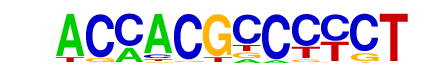 | 1*10^-43^ | 6.18 | 2.04 |
| 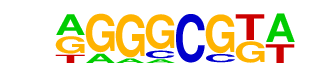 | 1*10^-43^ | 44.63 | 33.30 |
| 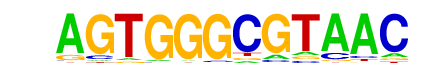 | 1*10^-35^ | 5.10 | 1.71 |
| 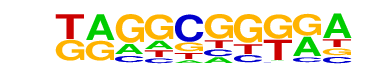 | 1*10^-31^ | 29.92 | 21.42 |
| 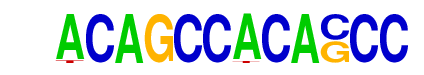 | 1*10-^18^ | 4.9 | 2.28 |
| 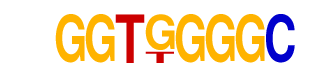 | 1*10^-18^ | 20.98 | 15.28 |
| 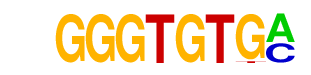 | 1*10^-18^ | 14.52 | 9.82 |
| 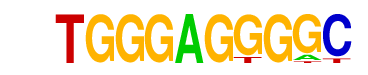 | 1*10^-16^ | 8.31 | 5 |
| 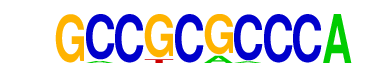 | 1*10^-8^ | 3.76 | 2.17 |
