# Supplementary material for: The Krüppel-like factor 9 cistrome in mouse hippocampal neurons reveals predominant transcriptional repression via proximal promoter binding
Source: BMC Genomics. 2017 Apr 13;18:299. doi: 10.1186/s12864-017-3640-7 (PMC5390390; doi:10.1186/s12864-017-3640-7)
Supplement: Supplementary file 10 — List of all DNA sequences found to be enriched above background at Klf9 ChSP peaks in HT22 [BirA/FLBIO-Klf9] cells. (DOCX 124 kb) [file 12864_2017_3640_MOESM10_ESM.docx]

| **Supplemental Table 4:** List of all DNA sequences found to be enriched above background at Klf9 ChSP peaks in HT22[BirA/FLBIO-Klf9] cells. The sequences are displayed as position weight matrices showing the relative frequency of each nucleotide at each position. | | | | |
| --- | --- | --- | --- | --- |
| HOMER motif | *p* value | % of peaks with motif | % of background sequences with motif | Closest match identified by HOMER |
| 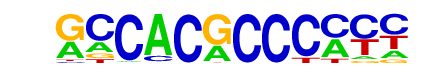^*^ | 1*10­^-702^ | 75.6 | 29.2 | Klf5 |
| 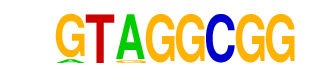 | 1*10^-61^ | 28.55 | 17.19 | Egr2 |
| 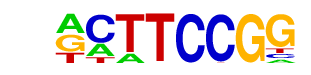 | 1*10^-43^ | 31.28 | 21.21 | Elk4 |
| 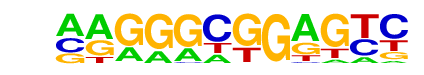 | 1*10^-34^ | 14.84 | 8.56 | Sp1 |
| 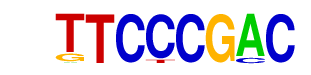 | 1*10^-31^ | 28.15 | 19.83 | E2F1 |
| 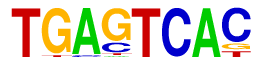 | 1*10^-28^ | 21.21 | 14.18 | Fosl1 |
| 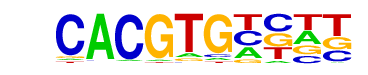 | 1*10^-28^ | 24.31 | 16.94 | CLOCK/Bmal1  (E-box) |
| 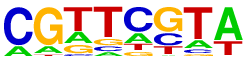 | 1*10^-22^ | 46.43 | 38.26 | Rfxdc2 |
| 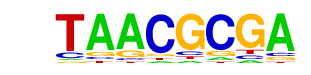 | 1*10^-20^ | 47 | 39.26 | Zbtb33 |
| 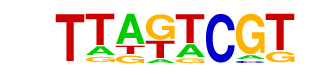 | 1*10^-18^ | 25.05 | 19.03 | P0031.1_IRC900814_1 |
| 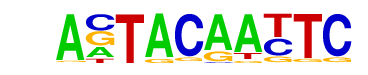 | 1*10^-17^ | 6.43 | 3.49 | GFY |
| 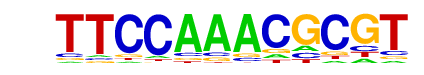 | 1*10^-15^ | 14.09 | 9.76 | NFATC2 |
| 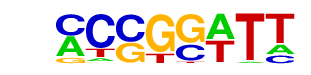 | 1*10^-15^ | 27.36 | 21.61 | Pitx1 |
| 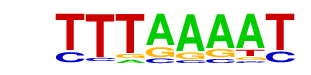 | 1*10^-14^ | 40.88 | 34.45 | CHR |
| 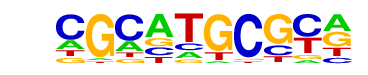 | 1*10^-14^ | 16.91 | 12.41 | NRF1 |
| 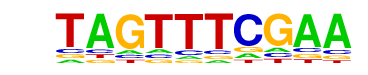 | 1*10^-13^ | 30.83 | 25.24 | Irf4 |
| 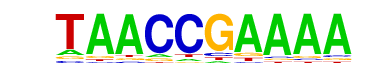 | 1*10^-12^ | 18.76 | 14.26 | TRP(MYB) |
| 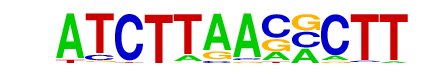 | 1*10^-12^ | 1.31 | 0.35 | TBR2 |
| 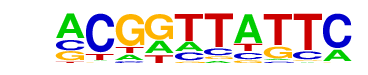 | 1*10^-12^ | 2.45 | 1.03 | MEF2a |

*Klf9 consensus motif
