# Supplementary material for: The Krüppel-like factor 9 cistrome in mouse hippocampal neurons reveals predominant transcriptional repression via proximal promoter binding
Source: BMC Genomics. 2017 Apr 13;18:299. doi: 10.1186/s12864-017-3640-7 (PMC5390390; doi:10.1186/s12864-017-3640-7)
Supplement: Supplementary file 13 — Subcloning of the 5′ upstream regions of Klf13, Klf16, Limk1 and Mapk11 into the pGL4.23 vector. (DOCX 15 kb) [file 12864_2017_3640_MOESM13_ESM.docx]

**Supplemental Table 5:** The 5’ upstream regions of *Klf13*, *Klf16*, *Limk1* and *Mapk11* were subcloned into the pGL4.23 vector. Sequences of the DNA fragments are given in the 5’ to 3’ direction. DNA sequences corresponding to Sp/Klf motifs identified by HOMER are in ALL CAPS; sequences matching the Klf9 consensus motif are in ALL CAPS UNDERLINED; nucleotides mutated in the *Klf13* promoter (the *Klf13*^Sp/Klf^ mutant) are **bold and in larger type.** Sites in the *Klf13* promoter are numbered 1-6 (in parentheses).

*Klf13*

caaaagctctggcatggaaagggccggcggggttcccgctgcgggaggcacgcgccccagccctgcgccgctacacagcggcccgccaccctcggacgtcatgcgtctgcgcgccgcgcgcccggctccgatt**TCCGCCCC**(1)tcctcttgcaggcccaggG**GCACGCCC**ACT(2)ggcgcgcgcaggtcccgcggccagtagaaccctctcgaggaccggattggctgcataaagtactcaagagccaatggagttcgcgtctaccactgGC**CACGCCC**CTC(3)ctgaggcgctccaggcTC**CCCGCCC**CCT(4)tcgtgcgcgctgctccctgcggccgctgccgcctgtaacctgcgccgccaggatgtggctgggggctgacgtcgggtccagatgtggccccgGC**CCCGCCC**AC(5)ccccggggccgg**TCCGCCC**ACA(6)ccgagccg

*Klf16*

tccctgtcccagtctcaaagggccagtgagggagggacgaaacaaccagaactccagtgtccctagcttcgcatagatgaccgagccagaccttcttattgcagccctgttcacttttgccataccggttatccacaggcctagtgattctcgagtcccggtgcgcaaagacgaactctacaaactcgatcagctaggcagggtagtgggtaccctacgctctcaagttcgacctccgggatattttgtccccaagccaagaatggcattggggacccagagatggggagtcaggattggtgggttattgccctgcttcaaggccaggaaggAGGGGGAGGGTCgcgcacgtggccCGGGGGCGGGGCagcgcgcttccgcacccccaccctggccacgtgcggatcgcactccccaccccatcccccgacacacacaccgcggacaggggcgcgtcccagtgcctgaatgtggagtgggaggagggacgagttggggaggggagagttcccggcgactgcgcaccgggcacagcgtggtgggcgcgccgggaagaggatggagcgcgcgcccggAGGAGGCGTGGCgcgcactgggggtAGGAGGCGGAGCatccgaggctggggtggggtgTGTGGGCGTGTCgcgggggTGGGCGTAACtctcaaagccttgggaggtgacgatccgggaagggcactggtgagggtagtgcgtaccaggccaggggaacgatttccctccgtttggcttgttcttacgtggagagggcacaatccagggatttggggaccccgggtgctctgctggtacacgaggggagggggggtttgaagtgcgggaggaccggcagctggcagtgtgaGCTCCGCCCAtcagcggtggcgcgcgactctctggaggaccgagggcgcggcaggAGGGGGCGTGTCcgcggtggggAGAGGGCGTGGCtactgagccccgagggggcgtcctccaggaggggcaggctgcggccgcccccgcgggctccggtgcgtcagggagcgtcaggcGGGGCGGGGCGGGACtgagcgcgggcggcggcggcgcgccggggcctcctcctcccttccctcctccgcctcccgc|actctagcagccgctgccggccggacgggcgtaggtcccaccgcctccgcttctctcccgccgccgattccgtgtctccgcgtccttgcccggcatgtcggcggccgtggcgtgtgtggattactttgccgccgacgtgctcatggccatctcctcgggagccgtggtgcatcgcggacgacccggccccgagggcgcgggccccgctgccggtctggatgtgcgcgccacgcgccgcgaggccacgccgccggggacccctggggcaccgccaccgccagctacggctccggggccgggaggcgccaccgctgcGCCCCACCtgctggccgccagcatcctggccgatctgaGAGGCGGGCCggtagtcgccaccgcagccagcaccgcggggggcacctctccggtctcctcctcctcagccgcttcgtctccctcttcggGCCGCGCCCCCGgcgccgccaaaagccaccgctgtcccttccatggctgcgccaaagcctattacaagtcttcgcacctcaagtcacacctgcggactcacacag|gtaagcccctcgtattttacacaggtgggatgcagggagtgcattagagaggtctcggggaccccgttcccccatctcacttggaaccccgaaactgcgtggcgcgtgGGGGGCGGGGAggggggcgcgcccaggcttcagcggcccgccggccgGGAGGGCGGGGAcgtGGTGGGTGGGGCgcgcgcgGGGAGGCGTGGCggccacgcggtcgggcggcgcgcggagggcggccctggtggccaaatctcccgcgattgcaggttgttcgggatctaccccggcatggtgggtccaccacgcgcgatcacagtcctcagccccctactccttcgttttcgcttggtattcttgtcctgtttttctgcgcttcatggtctcgcccttggtggtcagaacatccggttccgtagttctttctcggccctcccccagcctcagtttccttatctgtgaagagggtcggtagatccgggttacggccttctgggtagcagcgcggattccctccgaagggcagcgaagactctcgcgcagggtt

*Limk1*

tctgcatggctccacccgtcattggtaattaactttcagtggcttggaccagagaagcttcaggaagattgcatagagagagaggtgccttctcaagagatgcccggtgggcggctccattcaagctcacaacccagccacggggcacccaagtggagctgatgatgaatgaaaggtagagaaggggggtggagtcaGGGGCGGGTCttgctctGGGCGTGGtctggcaggctctgtggtcccagctgtgccgccgcgggtgaaactgccttcgtagacccgcacggcttccgccgaagaggaggagccacgcagaggcagcttcggccttcagcgccacggaagacccacccttccgctgttcacagactccgccgaattttccccCGAGGGCGTGGCtgttattaAGGGGGCGTGCCctctctcgaggaggcgcagtcccaagcagggacaattctgccaatgccgcagctggcagtcccctaaactggcctggAGAGGGCGTGGCtgcagctgagaagacttggtcaccgctccGGGGCGGGATtgttgcttGATGGGCGTGGTtatcgccgtGAAGGGCGGAGCcgcccaatgacagttccaacaggcagagccggcaagcctcttctcaccctgcaGAGGGCGGAtctgtctccgaggggcgaggctgcagctgcggaggcggagccgcgcaggggcagttccagccggccgcgtcgcgcgcgcGCCCCGCCCCTGccgcagctcgcaggcccgccccGGCCCGCCCCTCcgcgcttctcaggcagcggcaacgcg

*Mapk11*

tcctcaccctggccacaaggcgccttcctcagtggtgccctgagttggcgccgtggcgctggttttttaacctagagcctttacttccccctccccccaccactaatgttttttgttgttgttctgaaagtattatcaccacgtgctcaagctcacttttgacaatttcccttcatttctgaggaaggggactggcctgggacccggggaagggcagagggggtaggtaatatgcagaagatgaaggtggaggagtacaagcaagaagtttcatcgtcacccccatgggagggaagcaggctttttcctttgtcacggtctccgaggggcaggaagctgtgcgtctgtctccgggaaccctcaacctccacccaccccaccccacccccgttTCCCCGCCCTCCggtctccaggctggggaaatccgccgggcctcgGCCCCGCCCCgccGTCCCGCCCTgctgcgccgccgccccccagcccccaccccgctttgctcagcggtgctGGGCGTGGggcgcgggccgggtgctgcgcgcggggatccggggcgctcgctccagctgcttctgtggatatgtcgggtccgcgcgcgggattctaccggcaagagctgaacaaaacagtatgggaggtgccgcagcggctgcagggcctacgcccggtgggctccggcgcctacggctcagtctggtaggggccggcaaggacctggtgggagcTGGGCGGGAAagcgtgtgcgaccctccagcgcgacgtggggttcccggcgtggggaggggcctcgccctgccTCCGCCCCcggtctgcacgcttccgatgaatgaatgggggggaggggacacggaggaaactttcctactagattccactcgctggcaggtag
