# Supplementary material for: The Krüppel-like factor 9 cistrome in mouse hippocampal neurons reveals predominant transcriptional repression via proximal promoter binding
Source: BMC Genomics. 2017 Apr 13;18:299. doi: 10.1186/s12864-017-3640-7 (PMC5390390; doi:10.1186/s12864-017-3640-7)
Supplement: Supplementary file 15 — Description of Klf9 gene mutations introduced into HT22 cells by CRISPR/Cas9 genome editing. (DOCX 13 kb) [file 12864_2017_3640_MOESM15_ESM.docx]

**Supplemental Table 6:** We introduced mutations into the *Klf9* gene in HT22 cells by CRISPR/Cas9 genome editing. We targeted a sequence 82-103 bp downstream of the translation start site which is underlined in the “Wild-type sequence” indicated below; numbers on either side of the sequence indicate the distance in bp from the translation start site. We derived two subclonal lines (designated HT22 Klf9-CRISPR Line 1 and Line 2) with all three *Klf9* alleles deleted (see main text). We verified mutation by amplifying the *Klf9* gene from purified genomic DNA by PCR, subcloning the resulting fragments into pGEM T-easy, transforming *E. coli* with the cloned fragments and sequencing plasmid DNA. Sequencing results from the subcloned PCR fragments are shown below; dashes indicate deleted regions. DEL1 and DEL2 (present in both lines) causes a frameshift; DEL3 causes a substitution but no frameshift; DEL4 contains a large upstream deletion that eliminates the translation start site.

Wild-type sequence

(52)atctccaaccgcgccgccgtgccggagcacgggggcgctccggaagccgagcggctgcgactacctgagcgcgaggtgac(192)

Line 1 Colony 1 (DEL1)

(52)ATCTCCAACCGCGCCGCCGTGCCGG----------------------CGAGCGGCTGCGACTACCTGAGCGCGAGGTGAC(192)

Line 1 Colony 2 (DEL3)

(52)ATCTCCAACCGCGCCGCCGTGCCGGAGCACGGGGGCGCTCCGCG---CGAGCGGCTGCGACTACCTGAGCGCGAGGTGAC(192)

Line 1 Colony 3 (DEL2)

(52)ATCTCCAACCGCGCCGCC-------------------------------------------TACCTGAGCGCGAGGTGAC(192)

Line 1 Colony 4 (DEL1)

(52)ATCTCCAACCGCGCCGCCGTGCCGG----------------------CGAGCGGCTGCGACTACCTGAGCGCGAGGTGAC(192)

Line 1 Colony 5 (DEL1)

(52)ATCTCCAACCGCGCCGCCGTGCCGG----------------------CGAGCGGCTGCGACTACCTGAGCGCGAGGTGAC(192)

Line 1 Colony 6 (DEL2)

(52)ATCTCCAACCGCGCCGCC-------------------------------------------TACCTGAGCGCGAGGTGAC(192)

Line 1 Colony 7

(52)ATCTCCAACCGCGCCGCCGTGCCGGAGCACGGGGGCGCTCCGCG---CGAGCGGCTGCGACTACCTGAGCGCGAGGTGAC(192)

Line 1 Colony 8 (DEL1)

(52)ATCTCCAACCGCGCCGCCGTGCCGG----------------------CGAGCGGCTGCGACTACCTGAGCGCGAGGTGAC(192)

Line 1 Colony 10 (DEL1)

(52)ATCTCCAACCGCGCCGCCGTGCCGG----------------------CGAGCGGCTGCGACTACCTGAGCGCGAGGTGAC(192)

Line 2 Colony 1 (DEL2)

(52)ATCTCCAACCGCGCCGCC-------------------------------------------TACCTGAGCGCGAGGTGAC(192)

Line 2 Colony 2 (DEL1)

(52) ATNTCNNATCGNACCGCCNTGCCGNNG----------------------AGCGGATGCGATTACATNANCTCAAGGTGAC(192)

Line 2 Colony 3 (DEL1)

(52) ATNTCCAACCGTGNCGNNGTGCTGN----------------------CGAGCGGCTGCGACTACCTGAGCGCGAGGTGAC(192)

Line 2 Colony 4 (DEL1)

(52) ATNTCCAACCGTGCCGCNGTGCTGN----------------------CGAGCGGCTGCGACTACCTGAGCGCGAGGTGAC(192)

Line 2 Colony 5 (DEL1)

(52) ATCTCCAACCGCGCCGCCGTGCCGG----------------------CGAGCGGCTGCGACTACCTGAGCGCGAGGTGAC(192)

Line 2 Colony 7 (DEL4)

(52) -------ACCGCGCTCGTCGT-CCGAGGCCAGGGGGCGCTCCGCG---CGAGCGGCTGCGACTACCTGAGCGCGAGGTGAC(192)

Line 2 Colony 8 (DEL2)

(52) ATCTCCAACCGCGCCGCC-------------------------------------------TACCTGAGCNCGAGGTGAC(192)

Line 2 Colony 9 (DEL2)

(52) ATCTCCAACCGCGCCGCC-------------------------------------------TACCTGAGCGCGAGGTGAC(192)

Line 2 Colony 10 (DEL2)

(52) ATCTCCAACCGCGCCGCC-------------------------------------------TACCTGAGCGCGAGGTGAC(192)
