# Supplementary material for: The Krüppel-like factor 9 cistrome in mouse hippocampal neurons reveals predominant transcriptional repression via proximal promoter binding
Source: BMC Genomics. 2017 Apr 13;18:299. doi: 10.1186/s12864-017-3640-7 (PMC5390390; doi:10.1186/s12864-017-3640-7)
Supplement: Supplementary file 16 — List of all GO: PANTHER pathways enriched in genes with associated Klf9 ChSP peaks. (DOCX 16 kb) [file 12864_2017_3640_MOESM16_ESM.docx]

**Supplemental Table 7:** List of all GO:PANTHER pathways enriched in genes with associated Klf9 ChSP peaks. Pathways are ordered from the most to the least enriched based on the false discovery rate (FDR)-adjusted *p* value.

| Panther ID | Pathway Description | Number of Klf9 genomic targets in pathway | FDR-adjusted *p* value |
| --- | --- | --- | --- |
| Panther:P00006 | Apoptosis signaling pathway | 31 | 8.67E-11 |
| Panther:P00034 | Integrin signalling pathway | 36 | 2.79E-10 |
| Panther:P00031 | Inflammation mediated by chemokine and cytokine signaling pathway | 46 | 3.16E-10 |
| Panther:P00016 | Cytoskeletal regulation by Rho GTPase | 23 | 1.28E-09 |
| Panther:P00005 | Angiogenesis | 33 | 1.51E-09 |
| Panther:P00057 | Wnt signaling pathway | 42 | 1.75E-08 |
| Panther:P00056 | VEGF signaling pathway | 19 | 2.64E-08 |
| Panther:P00047 | PDGF signaling pathway | 28 | 2.82E-08 |
| Panther:P04393 | Ras Pathway | 20 | 7.85E-08 |
| Panther:P00021 | FGF signaling pathway | 24 | 4.71E-07 |
| Panther:P00059 | p53 pathway | 19 | 5.39E-07 |
| Panther:P00018 | EGF receptor signaling pathway | 24 | 7.78E-07 |
| Panther:P00049 | Parkinson disease | 21 | 1.01E-06 |
| Panther:P00019 | Endothelin signaling pathway | 19 | 1.02E-06 |
| Panther:P04398 | p53 pathway feedback loops 2 | 14 | 1.24E-06 |
| Panther:P00036 | Interleukin signaling pathway | 20 | 2.40E-06 |
| Panther:P00046 | Oxidative stress response | 14 | 3.13E-06 |
| Panther:P00048 | PI3 kinase pathway | 14 | 3.13E-06 |
| Panther:P00053 | T cell activation | 17 | 3.44E-06 |
| Panther:P00030 | Hypoxia response via HIF activation | 9 | 2.22E-05 |
| Panther:P00020 | FAS signaling pathway | 10 | 2.54E-05 |
| Panther:P00029 | Huntington disease | 22 | 7.04E-05 |
| Panther:P00033 | Insulin/IGF pathway-protein kinase B signaling cascade | 9 | 0.000192 |
| Panther:P00009 | Axon guidance mediated by netrin | 8 | 0.000292 |
| Panther:P00042 | Muscarinic acetylcholine receptor 1 and 3 signaling pathway | 13 | 0.000302 |
| Panther:P04391 | Oxytocin receptor mediated signaling pathway | 13 | 0.000302 |
| Panther:P00060 | Ubiquitin proteasome pathway | 11 | 0.000469 |
| Panther:P04394 | Thyrotropin-releasing hormone receptor signaling pathway | 13 | 0.000535 |
| Panther:P00003 | Alzheimer disease-amyloid secretase pathway | 13 | 0.000634 |
| Panther:P00040 | Metabotropic glutamate receptor group II pathway | 11 | 0.000691 |
| Panther:P00052 | TGF-beta signaling pathway | 16 | 0.000967 |
| Panther:P05731 | GABA-B receptor II signaling | 9 | 0.001307 |
| Panther:P04374 | 5HT2 type receptor mediated signaling pathway | 13 | 0.00169 |
| Panther:P04373 | 5HT1 type receptor mediated signaling pathway | 10 | 0.001719 |
| Panther:P04385 | Histamine H1 receptor mediated signaling pathway | 10 | 0.001719 |
| Panther:P04378 | Beta2 adrenergic receptor signaling pathway | 9 | 0.003732 |
| Panther:P04377 | Beta1 adrenergic receptor signaling pathway | 9 | 0.003732 |
| Panther:P04397 | p53 pathway by glucose deprivation | 6 | 0.004729 |
| Panther:P02738 | De novo purine biosynthesis | 7 | 0.004816 |
| Panther:P00002 | Alpha adrenergic receptor signaling pathway | 5 | 0.008127 |
| Panther:P00027 | Heterotrimeric G-protein signaling pathway-Gq alpha and Go alpha mediated pathway | 16 | 0.008158 |
| Panther:P00015 | Circadian clock system | 4 | 0.008407 |
| Panther:P00043 | Muscarinic acetylcholine receptor 2 and 4 signaling pathway | 10 | 0.010262 |
| Panther:P00010 | B cell activation | 10 | 0.010262 |
| Panther:P04396 | Vitamin D metabolism and pathway | 4 | 0.012293 |
| Panther:P04386 | Histamine H2 receptor mediated signaling pathway | 6 | 0.012381 |
| Panther:P00044 | Nicotinic acetylcholine receptor signaling pathway | 11 | 0.014006 |
| Panther:P02762 | Pentose phosphate pathway | 2 | 0.014067 |
| Panther:P04380 | Cortocotropin releasing factor receptor signaling pathway | 7 | 0.014688 |
| Panther:P04392 | P53 pathway feedback loops 1 | 3 | 0.016557 |
| Panther:P00007 | Axon guidance mediated by semaphorins | 5 | 0.01658 |
| Panther:P00026 | Heterotrimeric G-protein signaling pathway-Gi alpha and Gs alpha mediated pathway | 18 | 0.018464 |
| Panther:P04372 | 5-Hydroxytryptamine degredation | 5 | 0.020319 |
| Panther:P00032 | Insulin/IGF pathway-mitogen activated protein kinase kinase/MAP kinase cascade | 6 | 0.020567 |
| Panther:P00004 | Alzheimer disease-presenilin pathway | 15 | 0.021028 |
| Panther:P00039 | Metabotropic glutamate receptor group III pathway | 10 | 0.021273 |
| Panther:P00041 | Metabotropic glutamate receptor group I pathway | 5 | 0.024059 |
| Panther:P04379 | Beta3 adrenergic receptor signaling pathway | 6 | 0.028093 |
| Panther:P05730 | Endogenous cannabinoid signaling | 5 | 0.029085 |
| Panther:P02776 | Serine glycine biosynthesis | 2 | 0.031379 |
| Panther:P00014 | Cholesterol biosynthesis | 3 | 0.048888 |
| Panther:P00035 | Interferon-gamma signaling pathway | 5 | 0.049698 |
