# Supplementary material for: The Krüppel-like factor 9 cistrome in mouse hippocampal neurons reveals predominant transcriptional repression via proximal promoter binding
Source: BMC Genomics. 2017 Apr 13;18:299. doi: 10.1186/s12864-017-3640-7 (PMC5390390; doi:10.1186/s12864-017-3640-7)
Supplement: Supplementary file 17 — Genes with peaks from different clusters were subjected to pathway analysis using GeneCoDis. (DOCX 15 kb) [file 12864_2017_3640_MOESM17_ESM.docx]

**Supplemental Table 8:** Genes with peaks from different clusters were subjected to pathway analysis using GeneCoDis. Pathways are listed in order of the most- to least-enriched based on the false discovery rate (FDR)-adjusted *p* value.

|  | |  |  | |  | |  |
| --- | --- | --- | --- | --- | --- | --- | --- |
| Cluster 1 |  | | |  | |  | |
| Panther ID | Pathway Description | | | Number of Klf9 genomic targets in pathway | | FDR-adjusted *p* value | |
| Panther:P00006 | Apoptosis signaling pathway | | | 19 | | 8.48E-07 | |
| Panther:P00057 | Wnt signaling pathway | | | 26 | | 1.17E-05 | |
| Panther:P00018 | EGF receptor signaling pathway | | | 14 | | 0.001042 | |
| Panther:P00034 | Integrin signalling pathway | | | 17 | | 0.00085 | |
| Panther:P04391 | Oxytocin receptor mediated signaling pathway | | | 10 | | 0.000698 | |
| Panther:P04394 | Thyrotropin-releasing hormone receptor signaling pathway | | | 10 | | 0.000985 | |
| Panther:P00049 | Parkinson disease | | | 12 | | 0.000878 | |
| Panther:P00021 | FGF signaling pathway | | | 13 | | 0.001149 | |
| Panther:P00009 | Axon guidance mediated by netrin | | | 6 | | 0.001358 | |
| Panther:P04385 | Histamine H1 receptor mediated signaling pathway | | | 8 | | 0.00175 | |
|  |  | | |  | |  | |
| Cluster 2 |  | | |  | |  | |
| Panther ID | Pathway Description | | | Number of Klf9 genomic targets in pathway | | FDR-adjusted *p* value | |
| Panther:P00016 | Cytoskeletal regulation by Rho GTPase | | | 10 | | 1.86E-05 | |
| Panther:P00040 | Metabotropic glutamate receptor group II pathway | | | 7 | | 4.22E-04 | |
| Panther:P04396 | Vitamin D metabolism and pathway | | | 4 | | 0.000649 | |
| Panther:P04378 | Beta2 adrenergic receptor signaling pathway | | | 6 | | 0.000848 | |
| Panther:P04377 | Beta1 adrenergic receptor signaling pathway | | | 6 | | 0.000848 | |
| Panther:P04373 | 5HT1 type receptor mediated signaling pathway | | | 6 | | 0.001129 | |
| Panther:P00039 | Metabotropic glutamate receptor group III pathway | | | 7 | | 0.001094 | |
| Panther:P00031 | Inflammation mediated by chemokine and cytokine signaling pathway | | | 13 | | 0.00101 | |
| Panther:P05731 | GABA-B receptor II signaling | | | 5 | | 0.002382 | |
| Panther:P00043 | Muscarinic acetylcholine receptor 2 and 4 signaling pathway | | | 6 | | 0.002771 | |
|  |  | | |  | |  | |
| Cluster 3 |  | | |  | |  | |
| Panther ID | Pathway Description | | | Number of Klf9 genomic targets in pathway | | FDR-adjusted *p* value | |
| Panther:P00047 | PDGF signaling pathway | | | 11 | | 5.14E-06 | |
| Panther:P00020 | FAS signaling pathway | | | 5 | | 3.34E-04 | |
| Panther:P00016 | Cytoskeletal regulation by Rho GTPase | | | 7 | | 0.000272 | |
| Panther:P00005 | Angiogenesis | | | 9 | | 0.000357 | |
| Panther:P00034 | Integrin signalling pathway | | | 9 | | 0.000571 | |
| Panther:P00029 | Huntington disease | | | 8 | | 0.000643 | |
| Panther:P00006 | Apoptosis signaling pathway | | | 7 | | 0.0016 | |
| Panther:P00036 | Interleukin signaling pathway | | | 6 | | 0.0025 | |
| Panther:P00056 | VEGF signaling pathway | | | 5 | | 0.002819 | |
| Panther:P00003 | Alzheimer disease-amyloid secretase pathway | | | 5 | | 0.003633 | |
