# Supplementary material for: The Krüppel-like factor 9 cistrome in mouse hippocampal neurons reveals predominant transcriptional repression via proximal promoter binding
Source: BMC Genomics. 2017 Apr 13;18:299. doi: 10.1186/s12864-017-3640-7 (PMC5390390; doi:10.1186/s12864-017-3640-7)
Supplement: Supplementary file 18 — Oligonucleotides used for reverse transcriptase quantitative PCR (RTqPCR), chromatin immunoprecipitation assays, subcloning and site-directed mutagenesis. (DOCX 14 kb) [file 12864_2017_3640_MOESM18_ESM.docx]

**Supplemental Table 9:** Oligonucleotides used for reverse transcriptase quantitative PCR (RTqPCR), chromatin immunoprecipitation assays, subcloning and site-directed mutagenesis.

**For RTqPCR**

Taqman assays

*Klf9* mRNA

Taqman probe: 5' 6FAM-AAAGTCTATGGAAAATCC 3'

Forward: 5’ GCACAAGTGCCCCTACAGT 3’

Reverse: 5' TGTATGCACTCTGTAATGGGCTTT 3'

*Gapdh* mRNA

Taqman probe: 5’ 6FAM-AAG GTC GGT GTG AAC G 3’

Forward: 5’ TCG TCC CGT AGA CAA AAT GGT 3’

Reverse: 5’ CGC CCA ATA CGG CCA AA 3’

Sybr green assays

**For RT-qPCR**

| Gene | Forward | Reverse |
| --- | --- | --- |
| *Klf13* mRNA | CTACAGCCGCTCCGATG | CTGTTTTGCTGTGGACTTCTC |
| *Apc2* mRNA | CTGCGTAATCTGTCATGGAGG | AGCACTGAGCACACTCTTTAG |
| *Smurf1* mRNA | TCACAGTTCCGTTCTACAAGC | CTCCACGCAGAAGGTATGATC |
| *Nlgn2* mRNA | TCTCCAAGAACGATGTCATGC | TCCACACTACCTCTTCAAAGC |
| *Nyap1* mRNA | CTGAACCCCGCAAGTCTG | TCAAGCCTCAAGGAGACCTAG |
| *Limk1* mRNA | ATCACAGAGTACATCAAGGC | GTTCATCGAATGGAGGTAGGC |
| *Mapk11* mRNA | CCCATGAAATTGAGCAGTGAG | GGTCCCAGGTTAAGTGTCAG |
| *Pou6f1* mRNA | GTGAGGGTCTGGTTCTGTAAT | AGTGACTTCGGCTGGTTG |
| *Klf16* mRNA | CTGTCCCTTCCATGGCTG | ATCAGAACTGGCGAACTTC |
| *Slc11a2* mRNA | GGGTTGGCAGTGTTTGATTG | CTGGGCTGTTAGTCATCTGG |
| *Klf11* mRNA | CAGGTAGACTTTTCCCGAAGG | TTCTTGTCACAGCCGTCC |

**For ChIP-qPCR**

| Locus | Forward | Reverse |
| --- | --- | --- |
| *Slc11a2* peak | GACATCCTCTGTGCCATC | GGGCTGTCCAGTTCTCTTGT |
| *Klf16* peak | GTACGCACTACCCTCACCAG | GGTGGGCGTAACTCTCAAAG |
| *Sin3a* peak | CGCCTCCTTAGCAGTGAAG | ACCGTTCCCAGTCTACAAGG |
| *Nr3c1* peak | CGAGGTGAAGGGAGAAATGT | CGGCCTTATCTGCTAGAAGTG |
| *Limk1* peak | CAGAATTGTCCCTGCTTGG | AGGGCGTGGCTGTTATTAAG |
| *Mapk11* peak | GGGTGGAGGTTGAGGGTT | TGCAGAAGATGAAGGTGGAG |
| *Klf11* peak | GCGTGCTTGGAGGAGATATT | CCCACAGTCCTACCAGAGGT |
| *Klf13* peak | TCAGAGTCGGCCTGTCTTAG | GAACTGCGACCACAACTTG |
| *Klf13* intron | GTGCCTTATTCTTATTTGCTATCCC | GATTCTGACTCCACCCTGAAC |
| *Klf16* intron | ACTAAACTCCACCCCACAAC | TCTTTCAAACACTCCCTCGC |
| *Nr3c1* intron | AAATACCACTCACCGAAGCC | GCTTTTGGGTATGCTGTTGAC |
| *Limk1* intron | GAGGACAAAGGGAACACAGG | GGTTACAGAGAAAGATCCCAGC |

**Primers for amplification of genomic regions for cloning into pGL4.23 vector**

| *Mapk11* promoter | ATAGAGCTCTCCTCACCCTGGCCACAAG | ATACTCGAGCCTGCCAGCGAGTGGAATCT |
| --- | --- | --- |
| *Limk1* Promoter | ATAGCTAGCGCGTTGCCGCTGCCTGAGAA | ATAGGTACCTCTGCATGGCTCCACCCGTCA |
| *Klf16* promoter | ATAGCTAGCCCTGTCCCAGTCTCAAAG | ATTAAGCTTAACCCTGCGCGAGAGTCTTC |
| *Klf13* promoter | AAGAGCTCCAAAAGCTCTGGCATGGAAAGGG | ATAAGCTTCGGCTCGGTGTGGGCGGACCGGCC |

**Primers for cloning *Klf9* cDNA into pCDNA4:TO and pEF1α-FLBIO vectors**

FLBIO

Forward: 5’ AAATGATCATCTCCGCGGCCGCCTAC 3’

Reverse: 5’ GCTCTAGATCACAAGGGGCTGGCAA 3’

TO

Forward: 5’ AACTCGAGATGTCCGCGGCCGCCTA 3’

Reverse: 5’ GCTCTAGATCACAAGGGGCTGGCAA 3’

**Primers for amplifying pEF1α-BirAV5-pABGH cassette**

Forward: 5’ ATAGCGGCCGCGCTCCGGTGCCCGTC 3’

Reverse: 5’ ATAGCTAGCGAGCCCACCGCATCCCC 3’

**Oligonucleotides used to make pGL4.23-3xBTE vector**

5’ CTAGAGAAGGAGGCGTGGCCAGAAGGAGGCGTGGCCAGAAGGAGGCGTGGCC 3’

5’ AGCTGGCCACGCCTCCTTCTGGCCACGCCTCCTTCTGGCCACGCCTCCTTCT 3’

**Oligonucleotides for site-directed mutagenesis of *Klf13* promoter**

GC-box 1

5’ CTCCTGAGGCGCTCCAGGCTCTTTTTTTCCTTCGTGCGCGCTGCTCCCT 3’

5’ AGGGAGCACGCGCACGAAGGAAAAAAAGAGCCTGGAGCGCCTCAGGAG 3’

GC-box 2

5’ GATGTGGCCCCGGCTTTTTTTACCCCCGGGGCCGGTCCGCCCATATC 3’

5’ TGGGCGGACCGGCCCCGGGGGTAAAAAAAGCCGGGGCCACATC 3’

**Primers to verify mutation in Klf9-CRISPR knockout cells**

5’ GACACGTTTGCAGTCGAATAAAC 3’

5’ GGGCTTTAAGATGGGAGGATTT 3’

**gRNA sequences for CRISPR-Cas9 genome editing**

Round 1: 5’ GGGGCGCTCCGGAAGCCGAG 3’

5’ GCCGGAGCACGGGGGCGCTC 3’
